# A Role for Janus Cyclohexyl Ring Motifs in Medicinal Chemistry. Incorporation Into G‐Quadruplex Ligands and Anti‐*Trypanosoma brucei* Activity

**DOI:** 10.1002/cbic.70588

**Published:** 2026-10-30

**Authors:** Josephine M. Stewart, Mario Kehler, Vytautas Kuodis, Gary N. Parkinson, Wenqian Chen, Terry K. Smith, Zoë A. E. Waller, David O’Hagan

**Affiliations:** ^1^ EaStChem School of Chemistry BSRC University of St Andrews St Andrews UK; ^2^ UCL School of Pharmacy London UK

**Keywords:** African sleeping sickness, antitrypanosome, G‐quadruplex, organofluorine

## Abstract

The naphthalene diimide (NDI) ring system is recognized as a privileged motif for binding to G‐quadruplex DNA. Accordingly, compounds with peripheral groups attached to the NDI core are being explored as antiparasitic and anticancer candidates. In this study, we have synthesized and explored a series of NDIs with peripheral all‐*cis* ring fluorinated cyclohexyl rings to assess their relative stabilization potential for G‐quadruplex and double‐stranded DNA. This was explored through FRET melting experiments and measuring changes in melting temperatures (Δ*T*_m_). Compounds were also assayed against *Trypanosoma brucei* to assess their in vitro potency (EC_50_) against the causative parasite of human African trypanosomiasis or African Sleeping Sickness. The study revealed significant differences in activity when changing the nature of fluorine substitutions on the cyclohexane ring and also the length and nature (ether (O) or methylene (CH_2_)) of the linker to the core aromatic motif. The most active compound of the series was ether **12a** which displayed nanomolar activity (EC_50_ = 40 nM) against *T. brucei* although with a modest selectivity (14‐fold) for the parasite over *HeLa* (human model) cells. The study reveals good prospects for exploring fluorinated Janus cyclohexanes in a medicinal chemistry context.

## Graphical abstract

Naphthalene diimide (NDI) cores with peripheral fluorocyclohexyl ring systems are explored binding to G‐quadruplex DNA and were also assayed against the *Trypanosoma brucei* parasite. The most active compound of the series displayed nanomolar activity and suggested electrostatic interactions between the polarized cyclohexane and G‐Quad DNA.

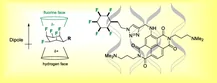

## 1 Introduction

Trypanosomatids are single‐cell kinetoplastid protozoan parasites, which can cause serious diseases in humans [1, 2]. *Trypanosoma brucei*, the causative agent of human African trypanosomiasis (HAT), is transmitted by the bite of an infected tsetse fly, whereupon the parasite multiples within the blood, reaching multiple organs but eventually crossing the blood brain barrier as a stage 2 infection, which is fatal without adequate treatments [3]. There are two forms of HAT. The first is found in Western and Central Africa caused by *T. brucei gambiense*, leading to a chronic disease state, typically with years between infection and death [4]. The second, in Eastern and Southern Africa caused by *T. brucei rhodesiense*, progresses within weeks to months to a chronic state and is often fatal [5].

The drugs used to treat these conditions are suboptimal at present [6, 7]. The arsenic‐containing therapy melarsoprol **1** in Figure 1 has been at the forefront of treatments for decades; however, drug resistance is a growing problem, particularly since the early 2000s [8]. A combination therapy of eflornithine **2** and nifurtimox **3** (NECT) [9, 10] is used to treat severe cases and requires intensive dosing and intravenous administration. Clinics are generally directed to the oral drugs, fexinidazole **4** and pentamidine **5** [11]; however, patients suffer from side effects, and fexinidazole **4** is ineffective against chronic cases of HAT (brain infection). Promising new therapies are emerging, and most notably, acoziborole **6**, an orally available, single dose, boron‐containing drug developed by the Drugs for Neglected Diseases initiative [12, 13]. It was recently (2026) given the green light by the European Medicine Agency (EMA) for rollout in regions where HAT is endemic and it appears to have significant advantages over the historical treatments. The introduction of acoziborole is encouraging in the fight to eradicate HAT; however, there remains a need for new therapies in this space.

**FIGURE 1:**
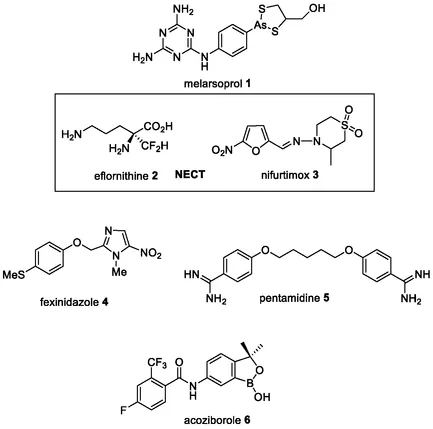
Current small molecule therapies for HAT.

In this study, we focus on G‐quadruplex‐DNA ligands as potential therapies against *T. brucei* [14]. Bioinformatic studies have revealed a high prevalence of putative G‐quadruplex‐forming sequences (PQS) within a number of trypanosomatid species, including *T. brucei* [15]. Over 29,000 PQS are estimated within the *T. brucei* genome. Their frequency of roughly 0.81 PQS per kb in the *T. brucei* genome is comparable to that in the human genome (0.9 PQS/kb) [14]. For this reason, G‐quadruplex ligands have been explored as both candidate antiparasite and also anticancer therapies [16].

There are already characterized G‐quadruplex‐forming sequences from *T. brucei* described in the literature, a sequence actually identical to the sequence from the human telomere (GGGTTA)_4_ [14] and a sequence from the promoter region of the *EBR1* gene [17]. This presents an opportunity to use G‐quadruplex ligands as a therapy against parasite infection. Currently, several structural series of G‐quadruplex ligands have been reported to display antitrypanosomal activity such as phenanthrolines, e.g., **7** [18], naphthalene diimides (NDI), e.g., **8** [19], and quinazolines, e.g., **9** [20], as shown in Figure 2.

**FIGURE 2:**
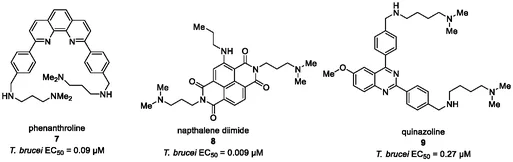
Examples of G‐quadruplex ligands with sub‐μM/nM anti‐*T brucei* activity [14, 15, 16].

Tangentially, we have developed an interest in partial fluorination of cyclohexanes as candidate motifs in medicinal chemistry, particularly all‐*syn* arrangements of the fluorines around the cyclohexane ring [21, 22]. When fluorines are placed on one face of the cyclohexane and hydrogens on the other in a chair conformation, as shown in Figure 3, this imparts an unusually high polarity to an otherwise aliphatic molecule [23, 24].

**FIGURE 3:**
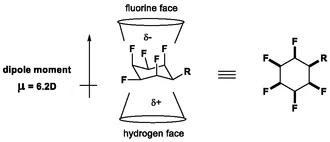
Janus‐face cyclohexyl motif displays a high molecular dipole and facial polarity.

In recent studies, we have introduced this ring motif into candidate COVID‐19 protease inhibitor skeletons [25] and also into peptides when attached as a side chain in an amino acid analogue [26]. In each case, the studies indicated properties of increased polarity (Lower Log*P*—the partition coefficient between octonal and water) relative to lesser fluorinated analogues. Given that these cyclohexane motifs become polar, the prospect of placing them at peripheral locations on a core motif that binds G‐quadruplex‐DNA emerged in order to assess if their polarity may enhance ligand affinity. To this end, we have selected the naphthalene diimides (NDI) core due to its ability to stabilize G‐quadruplex motifs [19, 27]. In particular, the hydrogen face of the fluorinated cyclohexane ring is electropositive and it was anticipated to have an affinity for the phosphate anion backbone, helping secure the ligand in the G‐quadruplex. The concept follows ligands developed by Morales et al. [19, 28, 29], who placed glucose motifs at a peripheral location, to explore polar contacts (hydrogen bonds) with the DNA phosphate backbone. Compounds, such as **10** (Figure 4), displayed good G‐quadruplex affinity and also excellent whole‐cell activity (EC_50_ 0.017 μM) against *T. brucei* with significant accumulation of the bioactive detected in the nucleus and kinetoplast of the parasites [30].

**FIGURE 4:**
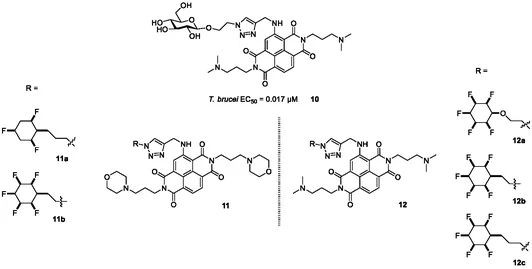
Carbohydrate–NDI conjugate **10** and lead targets **11** and **12** [19, 30].

## 2 Results and Discussion

In this study, we introduce fluorocyclohexyl rings as alternative candidate polar motifs to carbohydrates [21, 22] with an aim to help secure the NDI‐scaffolded ligands to the G‐quadruplex. Candidate structures **11a–b** and **12a–c** are shown in Figure 4. Propargylamines **21** and **22** were prepared as previously described [29, 30] by the route shown in Scheme 1. This involved treating dianhydride **13** with 1,3‐dibromo‐5,5‐dimethylhydantoin (DBH) [31] in concentrated sulfuric acid. The desired arylbromide **14** was generated but also with dibrominated product **15**. The mixture was not easily purified and was taken directly to the next step, involving microwave‐assisted diimide formation with either amines **16** or **17** in acetic acid. Products **18** and **19** could then be partially purified by chromatography, and they were then treated with propargylamine **20** in a S_N_Ar reaction. The resultant propargylamine products **21** and **22** were purified by HPLC. These were then treated with azides **23–** **26** in copper(I)‐catalyzed azide–alkyne cycloaddition (CuAAC) “click” reactions [32, 33, 34] to generate final products **11a–b** and **12a–c**.

**SCHEME 1:**
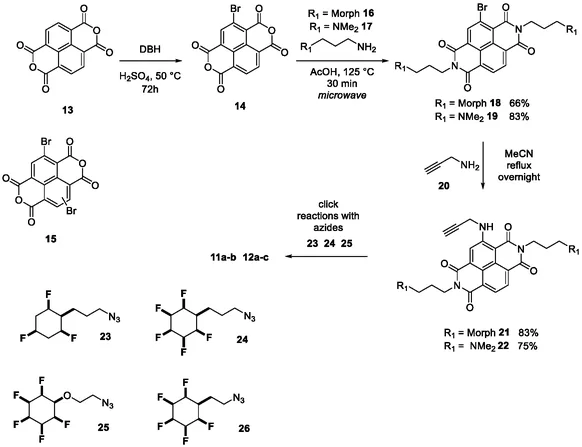
Synthetic routes for the synthesis of **11a–b** and **12a–c.**

Azides **23–25** are novel building blocks and were prepared as shown in Schemes 2 and 3. In each case, a key reaction involved fluoroaryl hydrogenations using the Rh‐CAAN N‐heterocyclic catalyst **33** adapting a protocol described by Glorius et al. [35, 36, 37].

**SCHEME 2:**
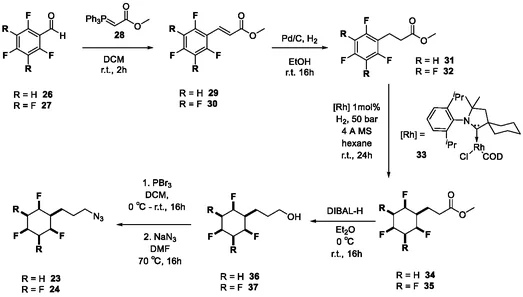
Synthetic route to azides **23** and **24**.

**SCHEME 3:**
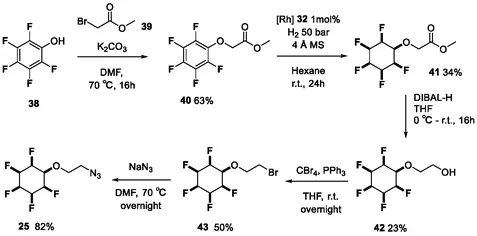
Synthesis route to azide **25**.

In the case of azides **23** and **24**, the route started with the appropriate fluoroaryl‐benzaldehyde **26** or **27**, as shown in Scheme 2.

Horner–Wadsworth–Emmons [38] reactions of benzaldehydes **26** and **27** with phosphonium ylid **28** generated conjugated esters **29** and **30**, respectively. A controlled hydrogenation with palladium on carbon, to remove the double bond, allowed saturated esters **31** and **32** to be prepared. These were each subjected to aryl hydrogenations using Rh catalyst **33** [35, 36, 37]. The corresponding cyclohexanes **34** and **35** were generated in each case, with the all‐*syn* stereochemistry as expected. Reduction of these esters with DIBAL generated alcohols **36** and **37**, which could be converted to the desired azides **23** and **24** through Appel bromination [39] and then azide displacement protocols.

Azide **25** was prepared as shown in Scheme 3 starting by introducing the ether linkage in a reaction of pentafluorophenol **38** with methyl bromoacetate **39**. From there, similar functional group transformations (ester to azide) as described above allowed the preparation of azide **25**. Azide **26** shown in Scheme 1 was also used in this study, and it was prepared as previously described [25].

Compounds **11a–b** and **12a–c** were all assessed for their ability to stabilize G‐quadruplex‐DNA, and they were assayed against the human telomeric (hTeloG) quadruplex, with the sequence 5′ ‐FAM‐[GGG‐TTA‐GGG‐TTA‐GGG‐TTA‐GGG]‐TAMRA‐3′ [40, 41]. Biphasic melting behavior was observed for hTeloG in the presence of several of the ligands, giving rise to two distinguishable melting transitions, denoted *T*_m_1 and *T*_m_2 for the lower and higher temperature transitions, respectively. Where biphasic melting was observed, Δ*T*_m_2 was used as the principal measure of ligand‐induced G4 stabilization because it represents the higher‐temperature transition and showed the most pronounced ligand‐dependent change across the compound series. The results obtained at 20 μM ligand concentration are shown in Table 1, and a wider range of concentration‐dependent data (ligand concentrations between 1 and 50 μM) are provided in Tables S1–S5.

**TABLE 1:** Change in melting temperatures of hTeloG and dsDNA upon addition of compounds **11a–b** and **12a–c** at 20 μM ligand concentration, measured using a FRET melting assay. Measurements were performed using 0.2 μM DNA in 10 mM sodium cacodylate buffer containing 10 mM KCl at pH 7.4. SI values represent the selectivity for hTeloG relative to dsDNA.

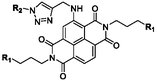			hTeloG		dsDNA	SI
Entry	R_1_	R_2_	Δ*T*_m_1, °C	Δ*T*_m_2, °C	Δ*T*_m_, °C	
**11a**	Morph	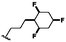	N/A	0.5 ± 0.7	0.5 ± 0.7	1.0
**11b**	Morph	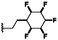	N/A	5.0 ± 0.0	2.0 ± 1.4	2.5
**12a**	─NMe_2_	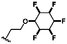	−7.0 ± 1.4	34.0 ± 0.0	4.0 ± 0.0	8.6
**12b**	─NMe_2_	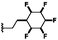	−5.0 ± 2.8	23.5 ± 2.1	4.5 ± 0.7	5.2
**12c**	─NMe_2_	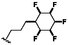	−3.5 ± 0.7	24.5 ± 0.7	6.0 ± 0.0	4.1

Two clear trends emerge: First, the dimethylamino NDIs **12a–c** have significantly higher Δ*T*_m_2 (23.5–34.0 °C) over their morpholino counterparts **11a–b** (0.5–5 °C). When comparing the matched pair **11b** and **12b**, a significant increase of Δ*T*_m_2 of 18.5 °C is observed by changing the terminal amine group. Second, the matched pairs **12b** and **12c** behave similarly despite **12b** having a two‐carbon linker and **12c** a three‐carbon linker to the cyclohexane. On the other hand, **11a** gives negligible stabilization relative to **11b**, which shows a significant increase of 5.0 °C in Δ*T*_m_2. By contrasting these outcomes, we attribute the better performance of **11b** to the increased number of fluorines (three versus five) relative to **11a**.

The ether linkage to the cyclohexane in **12a** further increases Δ*T*_m_2. When comparing **12a** (ether link) to matched pair **12c** (CH_2_ link), there is a clear increase in stabilization by 9.5 °C.

The lower stabilizations of the morpholino derivatives are likely attributed to the difference in p*K*_a_ between morpholine (p*K*_a_ = 8.4) and dimethylamine (p*K*_a_ = 10.8), where the increased basicity will result in easier protonation and a localization of positive charge [42]. This is exemplified when comparing **12b** (dimethylamine) and **11b** (morpholine), where the terminal dimethylamine substituents increase Δ*T*_m_2 by a further 18.5 °C.

The trifluorocyclohexane **11a** showed essentially no stabilization of the G‐quadruplex, with a Δ*T*_m_2 of 0.5 ± 0.7 °C. It is likely that the fluorine atoms sit equatorial in solution in **11a**, leading to a weak dipole, which would be less likely to interact with polar groups such as the phosphate backbone. This contrasts with the pentafluorinated rings in all other cases, which will always have at least two fluorines in axial orientations and inducing polarity. Introduction of the ether linkage in **12a** results in a significant increase in Δ*T*_m_2 compared to the carbon link in **12c**. The oxygen directly attached to the ring increases the molecular dipole of the ring and would support a more stabilizing interaction with the quadruplex.

Interestingly, the three dimethylamino compounds **12a–c** also show a second melting transition Δ*T*_m_1 at a lower temperature than the DNA alone (Table 1). This indicates that the compounds in some respect cause a destabilizing interaction by shifting the equilibrium to a less stable sub‐population. hTeloG has the possibility to form both parallel and antiparallel structures [43]. Of these, only the parallel form can provide the surface for π‐π stacking interactions with the NDI core [44, 45]. With this information, Δ*T*_m_1 may be due to the antiparallel form of the hTeloG quadruplex, whereas Δ*T*_m_2 may arise from a stabilization of the parallel form. It could be that the stronger stabilizers of the parallel form are more likely to destabilize the antiparallel form, consistent with only the dimethylamino ligands showing this second melting transition. Furthermore, the parallel form of hTeloG has been shown to be more biologically relevant [46]. This ability to selectively stabilize one form is of interest in the design of any potential drug molecules.

Compounds **11a–b** and **12a–c** were also tested against clearly defined double‐stranded DNA (dsDNA) using the sequence TATAGCTATA_6_TATAGCTATA [47] in order to explore any selectivity for G4‐quadruplex DNA over dsDNA. The results are also shown in Table 1 along with their respective selectivity (SI) for hTeloG. The results obtained at 20 μM ligand concentration are shown in Table 1, and a wider range of concentration‐dependent data (ligand concentrations between 1 and 50 μM) are provided in Tables S1–S5. Again, the dimethylamino compounds **12a–c** showed a higher stabilization of dsDNA relative to their morpholino counterparts **11a–b**. Indeed, a direct comparison of **12b** (dimethylamino) and **11b** (morpholine) shows a moderate increase in Δ*T*_m_ from 2.0 to 4.5 °C. All the compounds except **11a** showed some selectivity for hTeloG over dsDNA, with **12a** the best performer.

Following these in vitro studies, the compounds were then assessed for their antiparasitic efficacies in whole‐cell assays [48, 49]. In particular, assays were conducted against *T. brucei* and a selectivity screen against *HeLa* cells was conducted to differentiate between parasite and host toxicity. The outcomes are shown in Table 2.

**TABLE 2:** EC_50_ values measured for *T. brucei* and *HeLa* cell lines for the G4 ligands.

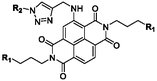			*T. brucei* EC_50_, μM	*HeLa* EC_50_, μM	SI
Entry	R_1_	R_2_			
**11a**	Morph	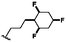	5.8 ± 0.52	>25	>4
**11b**	Morph	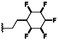	0.74 ± 0.1	17.8 ± 1.44	24
**12a**	─NMe_2_	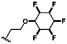	0.04 ± 0.01	0.54 ± 0.15	14
**12b**	─NMe_2_	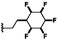	0.20 ± 0.01	0.78 ± 0.15	3.9
**12c**	─NMe_2_	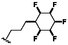	0.35 ± 0.01	0.73 ± 0.08	2.9

Some compounds had low micromolar activity, with ether **12a** showing the best trypanocidal activity. Although there is not always a direct correlation between Δ*T*_*m*_ and whole cell assays, there is again a tendency for the dimethylamines **12a–c** to perform better relative to their morpholino counterparts **11a–b**. Notably, the trifluorocyclohexane **11a** has the lowest efficacy of the series, almost an order of magnitude less potent than the next active compound. The ability here of this ring to adopt a (trifluoro) tri‐equatorial conformation reduces the dipole, and this will lower polarity, consistent with a poorer performance. In terms of selectivity for *T. brucei* parasites over the human *HeLa* cells, the morpholino series displayed slightly higher selectivity indexes, with **11b** the most selective of the series.

## 3 Conclusions

The synthesis of a series of privileged NDI structures bearing peripheral selectively fluorinated and polarized cyclohexane ring structures was prepared. The series was then explored for their ability to stabilize human telomeric G‐quadruplex DNA and also to act as antiparasitic agents against *T*. *brucei*. The most active compound of the series was **12a** (EC_50_ of 40 nM), which has nanomolar activity but with a modest selectivity (14‐fold) for *T*. *brucei* over *HeLa* cells. This outcome can be favorably compared in its activity against *T. brucei* to NDI compound **10** (EC_50_ of 17 nM) in Figure 4 previously reported by Morales et al. [19]. In that case, a glucose ring is located in place of the pentafluorocyclohexyl rings explored here. To a first approximation, these motifs share the common feature of a six‐membered ring with a similar shape and size profile. The Janus cyclohexanes do not, however, have the hydrogen bonding capability of a carbohydrate, although they have a larger molecular dipole and could form better electrostatic interactions with polar substituents. It remains to be determined how these ring systems interact with G‐quadruplex DNA. The polar–electrostatic aspect underlying the performance of the Janus rings is reinforced by the observation that compound **11a**, with only three fluorines, was a significantly poorer ligand and bioactive than all of the others studied. This is consistent with the expectation that this particular variant will adopt a less polar tri‐equatorial rather than triaxial conformation of the C─F bonds, not available to the other candidates, and display less attractive properties such as lower polarity and higher lipophilicity [50].

## Funding

This work was supported by the Biotechnology and Biological Sciences Research Council.

## Conflicts of Interest

The authors declare no conflicts of interest.

## Supporting information

Supplementary Material

## Data Availability

The data that support the findings of this study are available in the Supporting Information of this article.
