# Supplementary material for: A Role for Janus Cyclohexyl Ring Motifs in Medicinal Chemistry. Incorporation Into G‐Quadruplex Ligands and Anti‐Trypanosoma brucei Activity
Source: Chembiochem. 2026 Oct 30;27(21):e70588. doi: 10.1002/cbic.70588 (PMC13720963; doi:10.1002/cbic.70588)
Supplement: Supplementary file 1 [file CBIC-27-e70588-s001.pdf]

Supplementary Information

A role for Janus cyclohexyl ring motifs in medicinal chemistry. Incorporation into G-Quadruplex ligands and anti-*Trypanosoma brucei* activity

Josephine A. Stewart,[a] Mario Kehler,[a] Vytautas Kuodis,[a] Gary N. Parkinson,[b] Wenqian Chen,[b] Terry K. Smith[a]* Zoë A. E. Waller[b]* and David O'Hagan.[a]*

[a]EaStChem, School of Chemistry, BSRC, University of St Andrews, North Haugh, St Andrews, KY16 9ST, UK.
[b]UCL School of Pharmacy, 29-39 Brunswick Square, London WC1N 1AX, UK.

TABLE OF CONTENTS

**General experimental for synthesis**		**Page 2**
**(General procedure A)**	**Aryl hydrogenation of fluorinated arenes**	**Page 3**
**(General procedure B)**	**Reduction of methyl esters to alcohols with DIBAL-H**	
**(General procedure C)**	**Copper-catalysed Huisgen 1,3-dipolar cycloaddition "click"**	
**General experimental for the synthetic chemistry**		**Page 2**
**Synthesis and characterisation of prepared compounds**		**Page 4**
**NMR Spectra of final products**		**Page 20**
**Oligonucleotides**		**Page 27**
**FRET melting Assays**		**Page 27**
**Anti-parasite assays (*T. brucei*)**		**Page 38**
**References for Supplementary Information**		**Page 39**

General experimental for the synthetic chemistry

All reactions carried out under air in standard laboratory glassware unless otherwise stated. Commercially available solvents were purchased from Arcos, Alfa Aesar, Fisher Scientific, Fluorochem, Sigma Aldrich and TCI and used as received unless otherwise stated. Dry solvents (toluene, THF, hexane, diethyl ether and DCM) were dispensed from an MBraun SPS-800 solvent system. Commercially available reagents were purchased from Sigma Aldrich, BLD Pharm, Fluorochem, Fisher Scientific and Apollo Scientific. Room temperature refers to the temperature range of 15-25 °C. Under reduced pressure refers to use of a rotary evaporator with membrane pump at 30-50 mbar. Microwave reactions were performed in a Biotage Initiator + microwave reactor.

Flash column chromatography was performed with Sigma-Aldrich silica gel, 60 Å pore size and 230-400 mesh, 40-63 μm particle size under 5 psi compressed air. Columns performed both manually and on a Biotage Selekt 2 system with a UV/vis detector (200-400 nm), cartridge sizes from 10 g to 100 g. Analytical TLC was performed on pre-coated aluminium plates (Kieselgel 60 F254 silica) and visualisation achieved with UV light (254 nm) and/or staining with aqueous $KMnO_4$ solution and heating.

Semi-prep and analytical HPLC purifications and analyses were performed using a Shimadzu Prominence (SIL-20A HT autosampler, CL-20AT ternary pump, DGU-20A3R solvent degasser, SPD 20A UV detector and CBM-20A controller module) equipped with a Synergi 4 μM Polar-RP 80 Å LC column (250 x 10 mm) or Kinetix 2.6 μM C18 100 Å LC column (150 x 4.6 mm). Prep-HPLC purifications were performed using a Shimadzu Prominence (SIL-20A HT autosampler, two LC-20AR pumps and an SPD-M20A diode array detector) equipped with a Synergi 4 μm Polar-RP 80 Å LC column (250 x 21.2 mm). Chiral HPLC analysis was obtained on a Shimadzu HPLC comprising of a DGU-20A5R degassing unit, LC-20AD liquid chromatography pump, SIL-20AHT autosampler, SPD-20A UV/Vis detector and a CTO-20A column oven. Separation was achieved using a Daicel Chiralcel OD-H column.

Samples were freeze dried from frozen solutions in water in a Christ Alpha 1-2 LO Plus freeze drier.

NMR spectra were recorded on Bruker AVIII 500, AVIII-HD 500, AVII 400, AV 400 and Neo 400 spectrometers. NMR analyses were carried out at room temperature in indicated deuterated solvents. Chemical shift data are reported as δ in units of ppm relative to the respective deuterated solvent. Coupling constants *J* are reported in Hz. ^{1}H spectra recorded at

400 and 500 MHz, $^{13}C\{^{1}H\}$ recorded at 101, 126 and 176 MHz and $^{19}F\{^{1}H\}$ spectra were recorded at 377 and 470 MHz. Structural assignments made with additional information from COSY, ^{1}H-^{13}C HSQC, ^{1}H-^{13}C and ^{1}H-^{19}F HMBC spectra, reported when relevant. Multiplicities are denoted as s (singlet), br s (broad singlet), d (doublet), t (triplet), q (quartet) and m (multiplet). Ar denotes an aromatic, 4ry denotes quaternary in ^{13}C NMR.
High resolution mass spectra were recorded on a Thermo Scientific Exactive Orbitrap mass spectrometer. Spectra were acquired in electrospray ionisation (ESI) mode.

(General procedure A) Aryl hydrogenation of fluorinated arenes

Following a literature procedure,[1] fluoroarene (1 eq), activated 4 Å molecular sieves (10 by wt.) and the catalyst (1 mol%) were suspended in hexane (40 V) and placed inside an autoclave. The autoclave was pressurised with H_2 to 50 bar and stirred at room temperature for 24-120 h. The resulting mixture was filtered, and the solvent was removed to afford the product which was purified by silica gel column chromatography (60% dichloromethane in hexane) to afford the cyclohexane product.

(General procedure B) Reduction of methyl esters to alcohols with DIBAL-H

Following a literature procedure,[1] the ester (1 eq) was dissolved in dry THF (50 V) at 0 °C, DIBAL-H (4 eq) was added dropwise and solution was allowed to warm to room temperature and stir overnight. The reaction mixture was diluted with diethyl ether, cooled to 0 °C and the reaction was then quenched by the slow addition of water (2 mL), aqueous NaOH (15% w/v, 2mL) and water (2 mL). The mixture was warmed to room temperature and stirred for 15 min before $MgSO_4$ was added, the resulting suspension was stirred for a further 15 min. The suspension was filtered to remove aluminium salts and the filtrate was concentrated under reduced pressure to afford the alcohol product.

(General procedure C) Copper-catalysed Huisgen 1,3-dipolar cycloaddition "click"

Copper (II) sulfate pentahydrate (15 mol%), sodium ascorbate (1 eq), and the alkyne (1 eq) were dissolved in water (1 mL) and stirred. To this was added a solution of azide (1.5 eq) in *tert*-butanol (1 mL). The reaction mixture was stirred at room temperature overnight. Solvent was removed and the product was purified by HPLC.

Synthesis and Characterisation of prepared compounds

2-Bromo-1,4,5,8-naphthalenetetracarboxylic acid dianhydride (14)

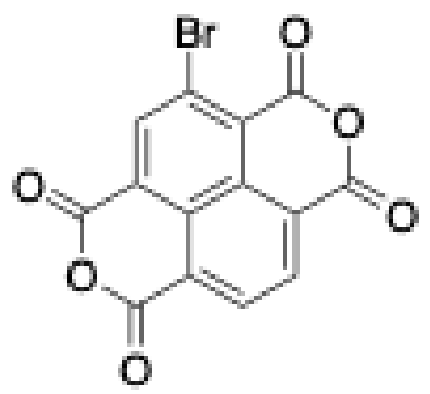

1,4,5,8-Naphthalenetetracarboyclic acid diahydride **13** (5.00 g, 18.6 mmol) and DBDMH (5.32 g, 18.6 mmol) were suspended in concentrated H_2SO_4 (50 mL) and the flask was sealed with a screw top. The reaction mixture was heated to 80 °C with stirring for 72 h. The mixture was cooled to room temperature before pouring over ice (300 mL). The resulting precipitate was filtered and washed with water to afford the crude product. This was subsequently recrystallised from DMF to afford a mixture of products (monobromo **14** 40%, giving a 30% yield of **14**).

^{1}H NMR (DMSO, 400 MHz) δ 8.70 (s, 1H, Ar**H**), 8.57 (d, *J* = 7.6, 1H, Ar**H**), 8.21 (d, *J* = 7.5 Hz, 1H, Ar**H**).

Data in accordance with literature.[2]

2-Bromo dimethylamino NDI (19)

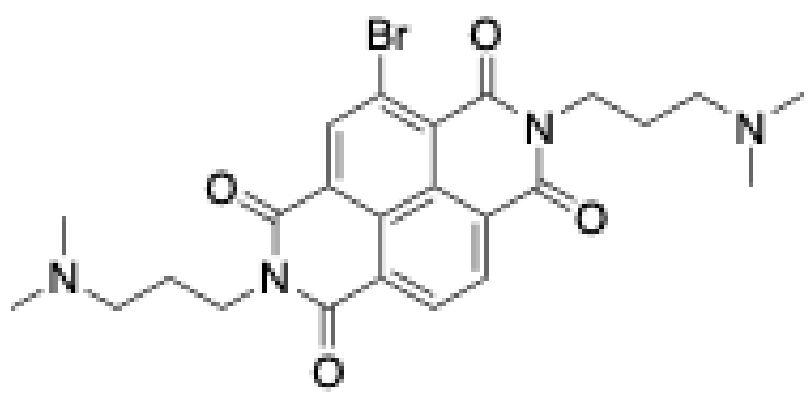

The mixture containing **14** (194 mg, approx. 0.22 mmol **14**) was suspended in AcOH (2 mL) in a microwave vial and sonicated for 20 min. To this was added 3-dimethylaminopropanamine **17** (211 μL, 1.68 mmol) dropwise. The vial was sealed and treated for 30 min at 130 °C in a microwave reactor. The resulting solution was basified with potassium carbonate solution before being extracted into chloroform (6 x 10 mL). The organic layers were combined and dried over $MgSO_4$ before concentrating under reduced pressure. The resulting oily residue was purified by silica gel column chromatography (4% MeOH in $CHCl_3$) to give a mixture of products containing 83% yield of **19**.

^{1}H NMR (500 MHz, $CDCl_3$) δ 8.94 (s, 1H, Ar**H**), 8.82 (d, *J* = 7.6 Hz, 1H, Ar**H**), 8.77 (d, *J* = 7.7 Hz, 1H, Ar**H**), 4.31 – 4.23 (m, 4H, NC**H**$_2$),* 2.49 – 2.36 (m, 4H, C**H**$_2$N),* 2.24 (s, 6H, N(C**H**$_3$)$_2$), 2.23 (s, 6H, N(C**H**$_3$)$_2$), 1.93 (m, 4H, C**H**$_2$).*

Data in accordance with literature.[3]

*Overlapping with signals from other co-products.

2-Propargylamine dimethylamino NDI (22)

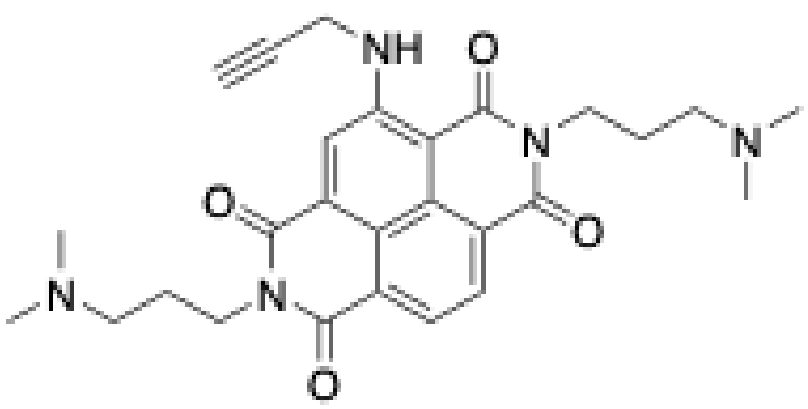

Propargylamine **20** (20 µL, 0.3 mmol) was added to a solution of **19** (25 mg, approx. 0.03 mmol of **19**) in acetonitrile (25 mL) in a screw top flask. The flask was sealed and heated to reflux with stirring for 24 h. On completion of the reaction, solvent was removed and the residue was purified by prep-HPLC (25-60% MeCN in H_2O with 0.1% TFA over 30 min) to give **22** as a red solid (10.1 mg, 75%).

^{1}H NMR (400 MHz, Acetone) δ 10.07 (t, *J* = 5.7 Hz, 1H, N**H**), 8.40 (d, *J* = 7.7 Hz, 1H, Ar**H**), 8.16 (d, *J* = 7.9 Hz, 1H, Ar**H**), 8.13 (s, 1H, Ar**H**), 4.59 (m, 2H, NHC**H**$_2$), 4.24 – 4.16 (m, 6H, NC**H**$_2$), 3.44 – 3.32 (m, 4H, C**H**$_2$N), 3.00 (t, *J* = 2.4 Hz, 1H, C≡C**H**), 2.96 (s, 6H, N(C**H**$_3$)$_2$), 2.95 (s, 6H, N(C**H**$_3$)$_2$), 2.34 – 2.22 (m, 4H, C**H**$_2$);

^{13}C{^{1}H} NMR (126 MHz, Acetone) δ 166.6 (4ry **C**=O), 164.0 (4ry **C**=O), 163.5 (2 x 4ry **C**=O), 152.2 (4ry Ar**C**NH), 131.7 (Ar**C**H), 129.6 (4ry Ar**C**), 128.6 (4ry Ar**C**), 127.0 (4ry Ar**C**), 125.4 (Ar**C**H), 124.2 (4ry Ar**C**), 120.2 (Ar**C**H), 120.1 (4ry Ar**C**), 101.2 (4ry Ar**C**), 80.1 (C≡**C**H), 74.5 (**C**≡CH), 56.0 (N**C**H$_2$), 55.9 (N**C**H$_2$), 42.9 (N(**C**H$_3$)$_2$), 38.7 (**C**H$_2$N), 38.2 (**C**H$_2$N), 33.1 (**C**H$_2$NH), 24.1 (**C**H$_2$).

HRMS (ESI) m/z $[C_{27}H_{32}O_4N_5]^+$ requires [M] 490.2449, found 490.2453.

Data in accordance with literature.[4]

2-Bromo morpholino NDI (18)

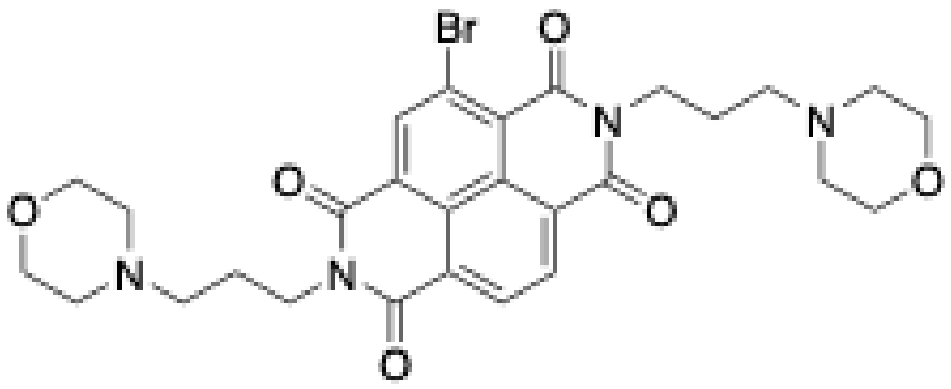

The mixture containing **14** (194 mg, approx. 0.22 mmol **14**) was suspended in AcOH (2 mL) in a microwave vial and sonicated for 20 min. 3-Morpholinopropanamine **16** (245 µL, 1.68 mmol) was then added dropwise. The vial was sealed and treated for 30 min at 130 °C in a microwave reactor. The resulting solution was basified with potassium carbonate solution before being extracted into chloroform (6 x 10 mL). The organic layers were combined and dried over $MgSO_4$ before concentrating under reduced pressure. The resulting oily residue was purified by silica gel column chromatography (4% MeOH in $CHCl_3$) to give a mixture of products containing **18** (154 mg, 66% yield).

^{1}H NMR (500 MHz, $CDCl_3$) δ 8.94 (s, 1H, Ar**H**), 8.82 (d, *J* = 7.6 Hz, 1H, Ar**H**), 8.77 (d, *J* = 7.6 Hz, 1H, Ar**H**), 4.34 – 4.26 (m, 4H, NC**H**$_2$), 3.55 (br s, 8H, morpholino O(C**H**$_2$)$_2$), 2.54 (br s, 4H, C**H**$_2$N), 2.44 (br s, 8H, morpholino N(C**H**$_2$)$_2$), 2.02 – 1.91 (m, 4H, C**H**$_2$).

Data in accordance with literature.[5]

2-Propargylamine morpholino NDI (21)

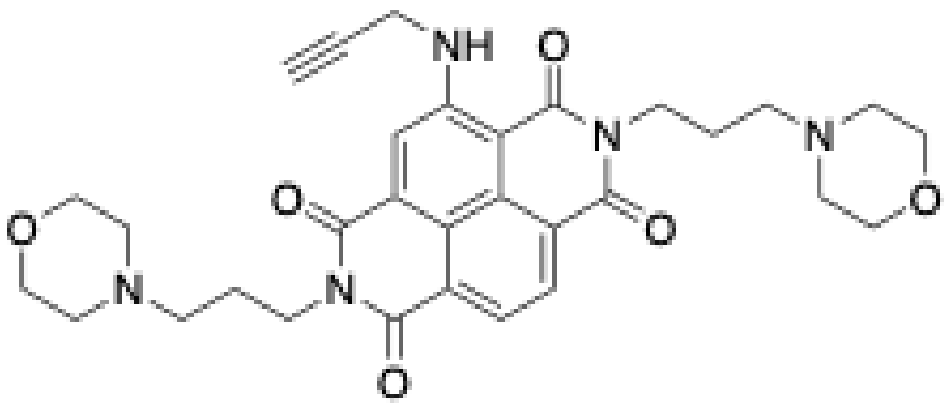

Propargylamine **20** (32 µL, 0.5 mmol) was added to a solution of **18** (50 mg, approx. 0.048 mmol of **18**) in acetonitrile (50 mL) in a screw top flask. The flask was sealed and heated to reflux with stirring for 24 h. On completion of the reaction, solvent was removed and the residue was purified by prep-HPLC (25-60% MeCN in H_2O with 0.1% TFA over 30 min) to give **21** as a red solid (10.1 mg, 75% yield).

^{1}H NMR (500 MHz, MeOD) δ 8.55 (d, *J* = 7.8 Hz, 1H, Ar**H**), 8.30 (s, 1H, Ar**H**), 8.29 (d, *J* = 7.4 Hz, 1H, Ar**H**), 4.49 (d, *J* = 2.6 Hz, 2H, C**H**$_2$NH), 4.30 (t, *J* = 6.8 Hz, 1H, N**C**H$_2$), 4.27 (t, *J* = 6.8 Hz, 1H, NC**H**$_2$), 4.10 – 4.03 (m, 4H, morpholino OC**H**$_2$), 3.77 (t, *J* = 12.0 Hz, 4H, C**H**$_2$N), 3.54 (br d, *J* = 12.3 Hz, 4H, morpholino OC**H**$_2$), 3.43 – 3.25 (m, 4H, morpholino NCH$_2$), 3.22 – 3.12 (m, 4H, morpholino N**C**H$_2$), 2.84 (t, *J* = 2.5 Hz, 1H, C≡C**H**), 2.32 – 2.15 (m, 2H, C**H**$_2$).

^{13}C NMR (126 MHz, MeOD) δ 167.0 (**C**=O), 165.1 (**C**=O), 165.0 (**C**=O), 164.4 (**C**=O), 154.9 (4ry Ar**C**-NH), 131.7 (Ar**C**H), 131.0 (4ry Ar**C**), 127.6 (4ry Ar**C**), 126.4 (Ar**C**H), 125.2 (Ar**C**H), 124.8 (4ry Ar**C**), 121.2 (4ry Ar**C**), 120.3 (4ry Ar**C**), 102.3 (4ry Ar**C**), 85.1 (C≡**C**H), 74.1 (**C**≡CH), 65.1 (morpholine **C**H_2O), 56.4 (N**C**H_2), 56.3 (N**C**H_2), 53.2 (morpholine **C**H_2N), 38.6 (N**C**H_2), 38.1 (N**C**H_2), 33.1 (**C**H_2NH), 23.8 (**C**H_2).

HRMS (ESI) m/z $[C_{31}H_{36}O_6N_5]^+$ requires [M] 574.2660, found 574.2660.

Phosphonium ylide (28)

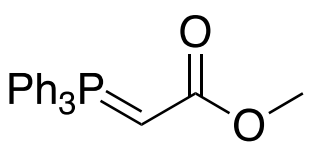

Methyl bromoacetate (1.45 mL, 15.3 mmol) was added to a solution of triphenylphosphine (4.03 g, 15.4 mmol) in toluene (100 mL). The solution was heated to reflux for 2h and left to stir overnight at room temperature. The precipitate was filtered off, washed with toluene (100 mL) and dried to afford the intermediate salt as a white solid. This was subsequently taken up in water (100 mL) and filtered through celite before being extracted with ether (50 mL). The aqueous solution was stirred rapidly and NaOH (0.709 g, 17.7 mmol) was added before being extracted twice with ethyl acetate (2 x 50 mL). The combined organic layers were then washed with water (50 mL) and dried over $MgSO_4$ before being concentrated under vacuum to give **28** as a white crystalline solid (4.38 g, 88%).

^{31}P NMR (202 MHz, $CDCl_3$) δ 17.97.

Data in accordance with the literature.[6]

Methyl pentafluorocinnamate (30)

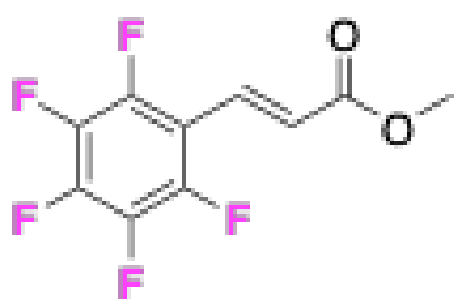

Pentafluorobenzaldehyde **27** (1.99 g, 10.1 mmol) was added to a solution of phosphonium ylide **28** (3.69 g, 11.0 mmol) in dichloromethane (100 mL), and the reaction allowed to stir for 2h at room temperature. The solvent was removed under reduced pressure and resulting mixture was purified by silica gel column chromatography (5% EtOAc in hexane), to afford **30** as a colourless oil (2.24 g, 88%).

^{1}H NMR (400 MHz, $CDCl_3$) δ 7.65 (d, J = 16.5 Hz, 1H, ArCH=C**H**), 6.74 (d, J = 16.5 Hz, 1H, ArC**H**=CH), 3.84 (s, 3H, C$\mathbf{H}_3$);

$^{19}F\{^1H\}$ NMR (376 MHz, $CDCl_3$) δ -139.5 – -139.7 (m), -151.2 (t, J = 20.6 Hz), -161.7 (td, J = 19.8, 6.1 Hz);

$^{13}C\{^1H\}$ NMR (101 MHz, $CDCl_3$) δ 166.5 (**C**=O), 147.4 – 144.0 (m, Ar**C**F), 143.6 – 139.8 (m, Ar**C**F), 139.6 – 136.2 (m, Ar**C**F), 128.5 (ArCH=**C**H), 126.0 (td, J = 8.7, 2.9 Hz ArCH=CH), 110.8 – 109.4 (m, Ar**C**-CH), 52.2 (**C**H_3).

Data in accordance with literature.[7]

Methyl 3-(pentafluorophenyl)propanoate (32)

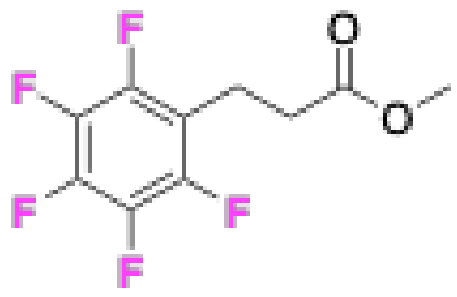

Pd/C 10% by wt. (112 mg) was added to a solution of methyl pentafluorocinnamate **30** (1.90 g, 7.50 mmol) in ethanol (20 mL) under nitrogen atmosphere. The flask was evacuated and purged three times with H_2, and the reaction was allowed to stir under an H_2 atmosphere for 15h at room temperature. The mixture was filtered through celite and washed with ethanol (20 mL). The solvent was subsequently removed under vacuum to afford **32** as a pale-yellow oil (1.76 g, 92%).

^1H NMR (500 MHz, $CDCl_3$) δ 3.69 (s, 3H, C**H**$_3$), 3.03 (t, J = 7.7 Hz, 2H, CH_2C**H**$_2$), 2.62 (t, J = 7.7 Hz, 2H, C**H**$_2CH_2$);

$^{19}F\{^1H\}$ NMR (376 MHz, $CDCl_3$) δ -140.3 – -145.9 (m), -157.1 (t, J = 20.6 Hz), -158.7 – -165.7 (m);

$^{13}C\{^1H\}$ NMR (101 MHz, $CDCl_3$) δ 172.2 (**C**=O), 147.2 – 143.8 (m, Ar**C**F), 141.9 – 135.7 (m, Ar**C**F x 2), 113.8 – 113.2 (m, Ar**C**-CH_2), 52.0 (O**C**H_3), 33.0 (**C**H_2C=O), 18.0 (**C**H_2CH_2).

Data in accordance with literature.[8]

Methyl 3-(all-*cis*-pentafluorocyclohexane)propanoate (35)

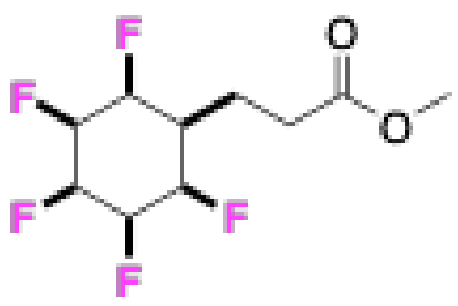

Ester **35** was prepared by General Procedure A from methyl 3-(pentafluorophenyl)propanoate **32** (1.04 g, 4.05 mmol), activated 4 Å molecular sieves (10 g), catalyst **33** (23 mg, 0.04 mmol) in hexane (40 mL) and stirring for 24h. Purification by silica gel column

chromatography (60% dichloromethane in hexane) afforded **35** as a white crystalline solid (357 mg, 35%).

^{1}H NMR (400 MHz, $CDCl_3$) δ 5.47 – 5.19 (m, 1H, C**H**F), 4.95 (d, *J* = 49.6 Hz, 2H, C**H**F), 4.43 (dt, *J* = 42.0, 27.0 Hz, 2H, C**H**F), 3.70 (s, 3H, C**H**$_3$), 2.55 (t, *J* = 6.9 Hz, 1H, C**H**$_2$C=O), 2.19 (q, *J* = 7.1 Hz, 1H, CHC**H**$_2$CH$_2$), 2.01 – 1.72 (m, 1H, C**H**CH$_2$);

^{19}F{^{1}H} NMR (377 MHz, $CDCl_3$) δ -203.4 (br s), -212.1 (d, *J* = 26.7 Hz), -216.7 (td, *J* = 27.1, 13.3 Hz);

^{13}C{^{1}H} NMR (126 MHz, $CDCl_3$) δ 185.6 (**C**=O), 88.6 – 85.1 (m, 5 x **C**HF), 52.0 (O**C**H$_3$), 36.9 (**C**HCH$_2$), 29.9 (**C**H$_2$C=O), 21.3 (CH**C**H$_2$).

HRMS (ESI) $[C_{10}H_{13}F_5O_2Na]^+$ requires [M] 283.0728, found 283.0719.

3-(All-*cis*-pentafluorocyclohexane)propanol (37)

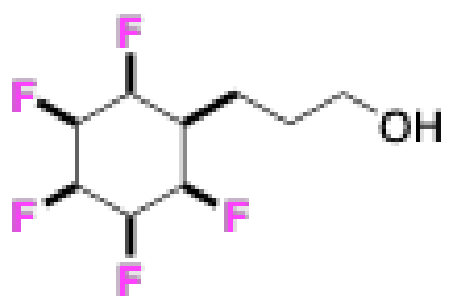

Alcohol **37** was prepared by General Procedure B from ester **35** (136 mg, 0.52 mmol) and DIBAL-H (1M in hexane, 2.3 mL) to afford **37** as a white crystalline solid (105 mg, 86%).

^{1}H NMR (500 MHz, DMSO) δ 5.36 (dt, *J* = 53.9, 7.4 Hz, 1H, C**H**F), 5.05 – 4.69 (m, 4H, 4 x C**H**F), 4.49 (t, *J* = 5.2 Hz, 1H, O**H**), 3.44 (q, *J* = 5.9 Hz, 2H, C**H**$_2$OH), 1.86 (tt, *J* = 36.3, 7.8 Hz, 1H, C**H**CH$_2$), 1.69 (q, *J* = 7.6 Hz, 2H, CHC**H**$_2$), 1.53 (p, *J* = 6.4 Hz, 2H, C**H**$_2$).

^{19}F{^{1}H} NMR (470 MHz, DMSO) δ -203.3 (tt, *J* = 8.3, 4.2 Hz), -212.0 (ddd, *J* = 23.8, 7.6, 4.3 Hz), -216.2 (tt, *J* = 23.8, 11.4 Hz).

^{13}C{^{1}H} NMR (126 MHz, DMSO) δ 89.0 – 85.2 (m, **C**HF), 60.5 (**C**H$_2$OH), 36.64 (tt, *J* = 18.8, 5.6 Hz, **C**HCH$_2$), 29.2 (**C**H$_2$), 22.3 (CH**C**H$_2$).

HRMS (ESI) $[C_9H_{13}OF_5Na]^+$ requires [M] 255.0776, found 255.0776.

3-(All-*cis*-pentafluorocyclohexane)propyl azide (24)

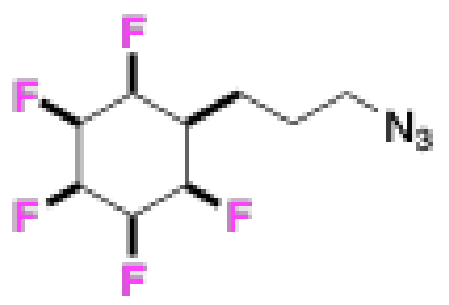

PBr_3 (61 µL, 0.32 mmol) was added dropwise to a solution of alcohol **37** (50 mg, 0.21 mmol) in dry DCM (2 mL) at 0 °C and the mixture was stirred for 1h. Saturated Na_2CO_3 solution was added until no further reaction and the mixture was extracted with DCM (3 x 5 mL). The combined organic layers were dried over $MgSO_4$ and solvent was removed to afford the alkyl bromide without purification. This material was immediately taken up in DMF (1 mL) and NaN_3 (8.8 mg, 0.14 mmol) was added and the solution heated to 70 °C for 16 h. Water (0.5 mL) was then added and the mixture was extracted with EtOAc (3 x 5 mL). The combined organic layers were washed with brine, dried over $MgSO_4$ and concentrated to afford **24** as a white solid (15 mg, 38% over two steps). This product was used directly in the "click" reactions.

^{1}H NMR (400 MHz, Acetone) δ 5.39 (dt, *J* = 53.3, 7.7 Hz, 1H, C**H**F), 5.17 – 4.73 (m, 4H, C**H**F), 3.45 (t, *J* = 6.7 Hz, 1H, C**H**$_2$N), 1.95 – 1.84 (m, 3H, overlapping C**H**CH_2 and CHC**H**$_2$), 1.84 – 1.64 (m, 2H, C**H**$_2$).

^{19}F{^{1}H} NMR (377 MHz, Acetone) δ -204.7 – -205.6 (m), -213.1 – -213.3 (m), -217.5 (ddd, *J* = 36.4, 24.9, 10.7 Hz).

2,4,6-Trifluorobenzaldehyde (26)

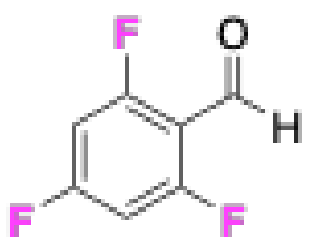

A solution of 1,3,5-trifluorobenzene (2.4 mL, 23 mmol) in dry THF (20 mL) was cooled to -60 °C under nitrogen, following a literature procedure.[9] ^{n}BuLi solution (2.5 M, 9 mL) was added dropwise with stirring, followed by DMF (1.8 mL, 23 mmol) and the reaction allowed to stir for 1h at -60 °C. The mixture was left to warm to room temperature before quenching with 4M HCl solution (30 mL). The organic layer was separated, and the aqueous layer was extracted with Et_2O (3 x 20 mL). The solvent was removed to afford **26** as a yellow solid (3.0 g, 83%).

^{1}H NMR (500 MHz, $CDCl_3$) δ 10.26 (s, 1H, C**H**O), 6.79 – 6.73 (m, 2H, Ar**H**).

^{19}F{^{1}H} NMR (377 MHz, $CDCl_3$) δ -95.5 (t, *J* = 11.4 Hz), -110.7 (d, *J* = 11.4 Hz).

^{13}C{^{1}H} NMR (101 MHz, $CDCl_3$) δ 183.2 (t, *J* = 4.3 Hz, **C**HO), 166.3 (dt, *J* = 260.1, 15.9 Hz, Ar**C**F), 164.3 (ddd, *J* = 264.7, 15.4, 8.5 Hz, Ar**C**F), 101.7 (td, *J* = 25.5, 3.9 Hz, Ar**H**). *

*Quaternary C not visible in ^{13}C NMR. Data in accordance with literature.[10]

Methyl (2,4,6-trifluoro)cinnamate (29)

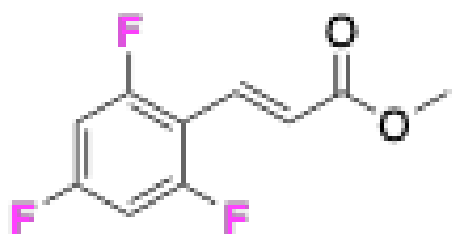

2,4,6-trifluorobenzaldehyde **26** (2.0 g, 12.5 mmol) was added to a solution of phosphonium ylide **28** (4.2 g, 12.5 mmol) in dichloromethane (100 mL), and the reaction was allowed to stir for 2h at room temperature. Solvent was removed under reduced pressure and resulting mixture was purified by silica gel column chromatography (5% EtOAc in hexane) to afford **29** as a white solid (2.0 g, 74%).

^{1}H NMR (500 MHz, $CDCl_3$) δ 7.70 (d, *J* = 16.5 Hz, 1H, ArCH=C**H**), 6.73 (t, *J* = 8.5 Hz, 2H, Ar**H**), 6.68 (d, *J* = 16.3 Hz, 1H, ArC**H**=CH), 3.82 (s, 3H, OC**H**$_3$).

^{19}F{^{1}H} NMR (377 MHz, $CDCl_3$) δ -104.1 (t, *J* = 8.0 Hz), -106.8 (d, *J* = 8.4 Hz).

^{13}C{^{1}H} NMR (101 MHz, $CDCl_3$) δ 167.3 (**C**=O), 163.3 (dt, *J* = 253.7, 16.9 Hz, Ar**C**F), 162.7 (t, *J* = 16.9 Hz, 4ry Ar**C**), 162.2 (ddd, *J* = 256.2, 14.9, 9.7 Hz, Ar**C**F), 130.2 (Ar-CH=**C**H), 123.5 (t, *J* = 7.3 Hz, Ar-**C**H=CH), 101.1 (td, *J* = 27.1, 3.5 Hz, Ar**C**H), 52.0 (**C**H$_3$).

Data in accordance with literature.[11]

Methyl 3-(2,4,6-trifluorphenyl)propanoate (31)

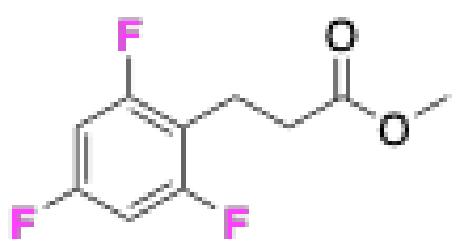

Pd/C (10% by wt., 100 mg) was added to a solution of methyl (2,4,6-trifluoro)cinnamate **29** (1.80 g, 8.3 mmol) in methanol (20 mL) under nitrogen atmosphere. The flask was evacuated and purged three times with H_2, and the reaction was allowed to stir under an H_2 atmosphere for 15h at room temperature. The mixture was filtered through celite and washed with ethanol (20 mL). The solvent was subsequently removed under vacuum to afford **31** as a pale-yellow oil (1.52 g, 84%).

^{1}H NMR (400 MHz, $CDCl_3$) δ 6.74 – 6.54 (m, 2H, Ar**H**), 3.68 (s, 3H, OC**H**$_3$), 2.95 (t, *J* = 7.9 Hz, 2H, Ar-C**H**$_2$), 2.58 (t, *J* = 7.6 Hz, 2H, C**H**$_2$CO).

^{19}F{^{1}H} NMR (377 MHz, $CDCl_3$) δ -110.6 – -110.8 (m), -112.5 – -112.8 (m).

$^{13}C\{^1H\}$ NMR (101 MHz, $CDCl_3$) δ 172.8 (**C**=O), 163.3 – 159.8 (m, overlapping Ar**C**F), 112.1 (t, *J* = 18.4 Hz, 4ry Ar**C**), 100.1 (t, *J* = 27.1 Hz, Ar**C**H), 51.8 (O**C**H_3), 33.5 (**C**H_2CO), 17.7 (ArC-**C**H_2).

HRMS (ESI) m/z $[C_{10}H_{10}F_3O_2]^+$ requires [M] 219.0627, found 219.0629.

Methyl 3-(all-*cis*-2,4,6-trifluorocyclohexane)propanoate (34)

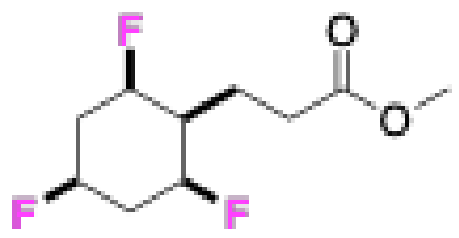

Ester **34** was prepared by General Procedure A from methyl 3-(2,4,6-trifluorophenyl)propanoate **31** (1.0 g, 4.6 mmol), activated 4 Å molecular sieves (10 g), catalyst **33** (41 mg, 0.05 mmol) in hexane (40 mL) and stirring for 120h. The product was purified by silica gel column chromatography (60% dichloromethane in hexane) to afford **34** as a white crystalline solid (348 mg, 34%).

^1H NMR (500 MHz, DMSO) δ 4.93 – 4.69 (m, 3H, overlapping 3 x C**H**F), 3.60 (s, 3H, OC**H**$_3$), 2.46 – 2.41 (m, 2H, CHC**H**$_2$), 2.40 – 2.29 (m, 2H, 2 x cyclohexane C**H**H), 1.91 (dt, *J* = 15.2, 3.0 Hz, 1H, C**H**CH$_2$), 1.83 (q, *J* = 7.0 Hz, 2H, C**H**$_2$CO), 1.85 – 1.75 (m, 2H, 2 x cyclohexane CH**H**).

$^{19}F\{^1H\}$ NMR (470 MHz, DMSO) δ -175.4 (t, *J* = 12.9 Hz), -190.6 (d, *J* = 13.4 Hz).

$^{13}C\{^1H\}$ NMR (126 MHz, DMSO) δ 173.2 (**C**=O), 87.4 (dd, *J* = 178.0, 4.1 Hz, **C**HF), 85.1 (d, *J* = 171.7 Hz, **C**HF), 51.3 (O**C**H_3), 40.0 (t, *J* = 19.8 Hz, **C**HCH$_2$), 33.6 (ddd, *J* = 20.4, 13.2, 7.3 Hz, cyclohexane **C**H_2), 30.4 (CH**C**H_2), 22.32 (t, *J* = 3.9 Hz, **C**H_3).

HRMS (ESI) m/z $[C_{10}H_{15}O_2F_3Na]^+$ requires [M] 247.0916, found 247.0915.

3-(all-*cis*-2,4,6-Trifluorocyclohexane)propanol (36)

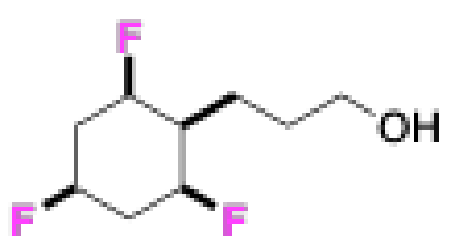

Alcohol **36** was prepared according to General Procedure B from ester **34** (80 mg, 0.36 mmol) and DIBAL-H (1M in hexane, 1.4 mL) to afford **36** as a white crystalline solid (64 mg, 93%).

^1H NMR (400 MHz, Acetone) δ 4.87 (dp, *J* = 47.4, 3.7 Hz, 1H, C**H**F), 4.80 (dq, *J* = 46.9, 3.4 Hz, 2H, C**H**F), 3.57 (t, *J* = 6.3 Hz, 2H, C**H**$_2$OH), 2.48 – 2.34 (m, 2H, 2 x cyclohexane C**H**H),

2.09 – 2.06 (m, 2H, 2 x cyclohexane CH**H**), 1.77 –1.60 (m, 5H, overlapping C**H**CH_2 and C$\mathbf{H}_2$).

$^{19}F\{^1H\}$ NMR (377 MHz, acetone) δ -177.3 (t, *J* = 12.1 Hz), -192.0 (d, *J* = 11.4 Hz).

$^{13}C\{^1H\}$ NMR (126 MHz, Acetone) δ 88.6 (ddd, *J* = 179.5, 4.4, 2.3 Hz, **C**HF), 86.1 (dt, *J* = 173.3, 2.5 Hz, **C**HF), 62.5 (**C**H_2OH), 42.6 (t, *J* = 19.3 Hz, cyclohexane **C**H_2), 35.1 (ddd, *J* = 20.7, 13.7, 6.9 Hz, **C**HCH_2), 31.0 (**C**H_2), 24.33 (t, *J* = 3.6 Hz, CH**C**H_2).

HRMS (ESI) m/z $[C_9H_{15}OF_3Na]^+$ requires [M] 219.0967, found 219.0965.

3-(all-*cis*-2,4,6-Trifluorocyclohexane)propyl azide (23)

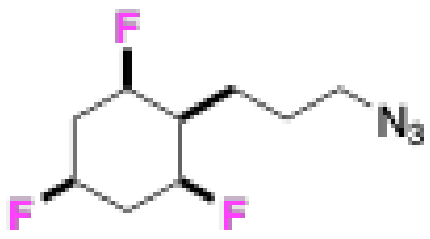

PBr_3 (73 µL, 0.38 mmol) was added dropwise to a solution of alcohol **36** (50 mg, 0.25 mmol) in dry DCM (2 mL) at 0 °C and the mixture was stirred for 1h. Saturated Na_2CO_3 solution was added until no further reaction and the mixture was extracted with DCM (3 x 5 mL). The combined organic layers were dried over $MgSO_4$ and the solvent was removed to afford the alkyl bromide without purification. This material was taken up directly in DMF (1 mL) and NaN_3 (10 mg, 0.15 mmol) was added. The solution was heated to 70 °C for 15h. Upon completion, water (0.5 mL) was added and the mixture was extracted with EtOAc (3 x 5 mL). The combined organic layers were washed with brine, dried over $MgSO_4$ and concentrated to afford **23** as a white solid (15 mg, 38% over two steps), and this was used immediately in the "click" reactions.

^1H NMR (400 MHz, $CDCl_3$) δ 4.99 – 4.68 (m, 3H, C**H**F), 3.35 (t, *J* = 6.5 Hz, 2H, C$\mathbf{H}_2$N), 2.70 – 2.47 (m, 2H, 2 x cyclohexane C**H**H), 1.88 – 1.69 (m, 6H, overlapping C$\mathbf{H}_2$).

$^{19}F\{^1H\}$ NMR (377 MHz, $CDCl_3$) δ -178.2 (t, *J* = 12.6 Hz), -191.7 (d, *J* = 12.7 Hz).

Methyl 2-(pentafluorophenoxy)acetate (40)

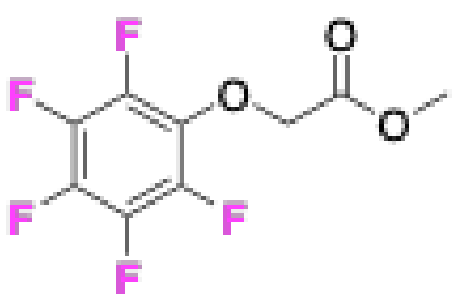

Solid K_2CO_3 (1.52 g, 11.0 mmol), followed by methyl 2-bromoacetate **39** (0.76 mL, 8.25 mmol) was added to a solution of pentafluorophenol **38** (1.0 g, 5.5 mmol) in DMF (10 mL) at room temperature and the resulting solution was heated at 70 °C for 15h. On completion, the

mixture was diluted with water (20 mL) and extracted with EtOAc (3 x 10 mL). The combined organic layers were washed with brine, dried over $MgSO_4$ and concentrated. The residue was purified by silica gel column chromatography (0-5% EtOAc in hexane) to give **40** as a colourless oil (840 mg, 63%).

^{1}H NMR ($CDCl_3$, 400 MHz) δ 4.77 (s, 2H, ArOC**H**$_2$), 3.77 (s, 3H, OC**H**$_3$);

^{19}F{^{1}H} NMR (CDCl3, 377 MHz) δ -156.7 – -156.3 (m), -163.7 – -162.8 (m).

^{13}C{^{1}H} NMR (126 MHz, $CDCl_3$) δ 168.3 (**C**=O), 141.4 (ddq, J = 248.8, 12.6, 4.0 Hz, Ar**C**F), 139.8 – 131.9 (m, Ar**C**F), 69.8 (t, J = 3.6 Hz, **C**H$_2$O), 52.6 (O**C**H$_3$).

HRMS (ESI) m/z $[C_9H_5O_3F_5Na]^+$ requires [M] 279.0051, found 279.0050.

Methyl 2-(all-*cis*-pentafluorocyclohexane)acetate (42)

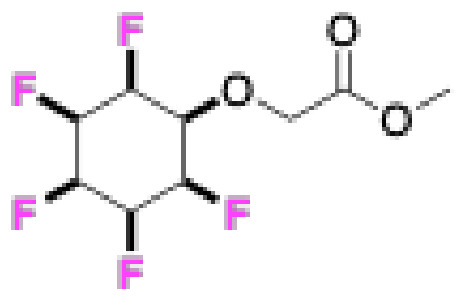

Ester **42** was prepared by General Procedure A from methyl 2-(pentafluorophenoxy)acetate **40** (900 mg, 3.5 mmol), activated 4 Å molecular sieves (7 g), catalyst **33** (20 mg, 0.04 mmol) in hexane (40 mL) and stirring for 24h. Purification by silica gel column chromatography (0-20% methanol in dichloromethane) afforded **41** as a white crystalline solid (315 mg, 34%).

^{1}H NMR (400 MHz, Acetone) δ 5.56 – 5.26 (m, 3H, C**H**F), 5.11 – 4.76 (m, 2H, C**H**F), 4.40 (s, 2H, OC**H**$_2$), 3.96 (br t, J = 29.2 Hz, 1H, C**H**O), 3.70 (s, 3H, OC**H**$_3$).

^{19}F{^{1}H} NMR (377 MHz, Acetone) δ -211.5 (dt, J = 10.7, 5.3 Hz), -217.0 (dt, J = 24.4, 5.3 Hz), -217.8 (tt, J = 25.2, 10.3 Hz).

^{13}C{^{1}H} NMR (126 MHz, Acetone) δ 171.3 (**C**=O), 90.0 – 84.7 (m, **C**HF), 73.8 – 73.1 (m, **C**HO), 66.9 (O**C**H$_2$), 52.1 (O**C**H$_3$).

HRMS (ESI) m/z $[C_9H_{11}O_3F_5Na]^+$ requires [M] 285.0521, found 285.0519.

2-(All-*cis*-pentafluorocyclohexane-oxy)ethanol (42)

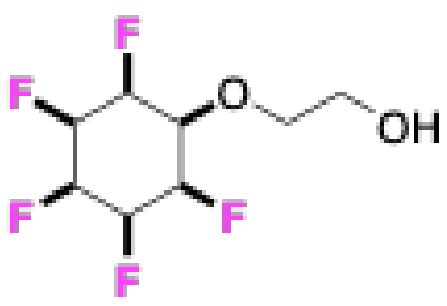

Alcohol **42** was prepared according to General Procedure B from ester **41** (300 mg, 1.14 mmol) and DIBAL-H (1M in hexane, 4.56 mL). The product was purified by silica gel column chromatography (0-100% EtOAc in hexane) to afford **42** as a white crystalline solid (61 mg, 23%).

^{1}H NMR (400 MHz, Acetone) δ 5.34 (dd, *J* = 49.9, 28.7 Hz, 3H, C**H**F), 4.95 (dt, *J* = 40.5, 27.0 Hz, 2H, C**H**F), 3.97 – 3.78 (m, 1H, C**H**O), 3.77 (t, *J* = 4.4 Hz, 2H, OC**H**$_2$), 3.70 (t, *J* = 4.4 Hz, 2H, C**H**$_2$OH).

^{19}F{^{1}H} NMR (377 MHz, Acetone) δ -211.3 (t, *J* = 9.3 Hz), -216.7 (dt, *J* = 25.6, 5.7 Hz), -217.6 – -217.8 (m).

^{13}C{^{1}H} NMR (126 MHz, Acetone) δ 90.3 – 85.1 (m, **C**HF), 74.2 – 73.4 (m, **C**HO), 72.23 (O**C**H$_2$), 62.1 (**C**H$_2$OH).

HRMS (ESI) m/z $[C_8H_{11}O_2F_5Na]^+$ requires [M] 257.0571, found 257.0569.

1-(2-Bromoethoxy)-all-*cis*-pentafluorocyclohexane (43)

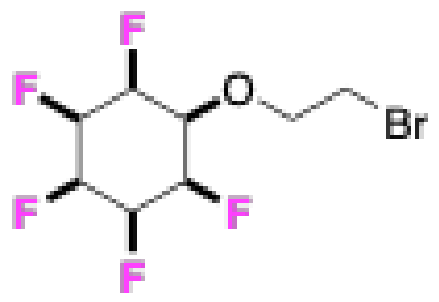

Triphenylphosphine (47 mg, 0.18 mmol) followed by tetrabromomethane (60 mg, 0.18 mmol) was added to a solution of alcohol **42** (20 mg, 0.09 mmol) in MeCN (1.5 mL) under nitrogen atmosphere and the reaction was allowed to stir at room temperature for 15h. On completion, the mixture was concentrated, and the residue purified by silica gel column chromatography (0-50% EtOAc in hexane) to give **43** as an off-white solid (13 mg, 50%).

^{1}H NMR (400 MHz, Acetone) δ 5.35 (dd, *J* = 52.0, 25.4 Hz, 3H, C**H**F), 4.96 (dt, *J* = 41.3, 26.7 Hz, 2H, C**H**F), 4.04 (t, *J* = 6.1 Hz, 2H, OC**H**$_2$), 3.92 (t, *J* = 29.8 Hz, 1H, C**H**O), 3.62 (t, *J* = 6.1 Hz, 2H, C**H**$_2$Br).

^{19}F{^{1}H} NMR (377 MHz, Acetone) δ -211.4, -216.7 (d, *J* = 25.1 Hz), -217.8 (tt, *J* = 24.7, 10.2 Hz).

^{13}C{^{1}H} NMR (101 MHz, Acetone) δ 90.3 – 87.8 (m, **C**HF), 88.0 – 86.0 (m, **C**HF), 86.0 – 84.0 (m, **C**HF), 75.2 – 72.3 (m, **C**HO), 70.4 (O**C**H$_2$), 31.3 (**C**H$_2$Br).

HRMS (ESI) m/z $[C_8H_{10}O^{79}BrF_5Na]^+$ requires [M] 318.9727, found 318.9728.

1-(2-Azidoethoxy)-all-*cis*-pentafluorocyclohexane (25)

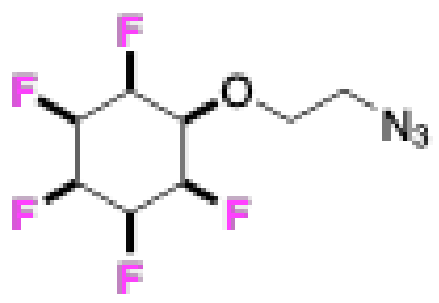

NaN_3 (4 mg, 0.07 mmol) was added to a solution of bromide **147** (10 mg, 0.03 mmol) in DMF (2 mL) and heated to 70 °C and stirred for 15h. On completion, the mixture was diluted with water and extracted with EtOAc (3 x 10 mL). The combined organic layers were washed with brine, dried over $MgSO_4$ and concentrated to afford **25** as a white solid (7 mg, 82%) which was used immediately in the 'click' reactions.

^{1}H NMR (CD_3OD, 500 MHz) δ 5.26 – 5.08 (3H, m, C**H**F), 4.78 – 4.54 (2H, m, C**H**F), 3.88 – 3.84 (3H, m, C**H**O and OC**H**$_2$), 3.48 – 3.42 (2H, m, C**H**$_2$N)

^{19}F{^{1}H} NMR (CD_3OD, 470 MHz) -212.2 (s), -217.4 (d, J = 25.1 Hz), -218.3 (dt, J = 25.1, 15.2 Hz).

Propyl trifluoro-Janus-face morpholino-NDI (11a)

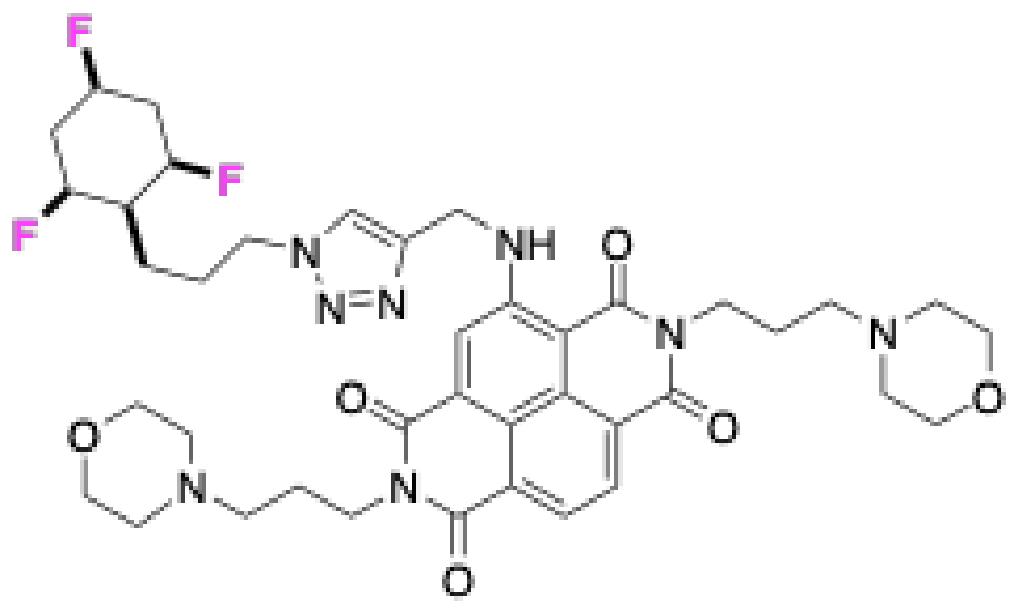

Triazole **11a** was prepared according to general procedure C from morpholino-NDI **21** (10 mg, 0.017 mmol), azide **23** (5.8 mg, 0.03 mmol), $CuSO_4.5H_2O$ (0.7 mg, 0.003 mmol) and sodium ascorbate (3.5 mg, 0.017 mmol). The product was purified by column chromatography (1-5% MeOH in DCM) to give **11a** as a red semi-solid (3.2 mg, 23%).

^{1}H NMR (500 MHz, Acetone) δ 10.56 (t, *J* = 5.8 Hz, 1H, N**H**), 8.61 (d, *J* = 7.8 Hz, 1H, Ar**H**), 8.46 (s, 1H, Ar**H**), 8.32 (d, *J* = 7.8 Hz, 1H, Ar**H**), 8.11 (s, 1H, triazole **H**), 5.06 (d, *J* = 5.8 Hz, 2H, C**H**$_2$NH), 4.85 (dq, *J* = 32.5, 3.6 Hz, 2H, C**H**F), 4.82 – 4.69 (m, 1H, C**H**F), 4.49 (t, *J* = 7.1 Hz, 2H, C**H**$_2$N-triazole), 4.30 – 4.16 (m, 4H, NDI-NC**H**$_2$), 3.43 (br s, 8H, morpholine OC**H**$_2$), 2.50 (br s, 4H, C**H**$_2$) 2.40 – 2.33 (m, 8H, morpholine NC**H**$_2$), 1.77 – 1.74 (m, 1H C**H**CH$_2$), 1.68 (q, *J* = 7.5 Hz, 2H, CHC**H**$_2$), 1.40 – 1.37 (m, 2H, C**H**$_2$).*

*CH$_2$ peak obscured by acetone satellite.

^{19}F{^{1}H} NMR (470 MHz, Acetone) δ -177.5 (t, *J* = 12.1 Hz), -191.9 (d, *J* = 12.4 Hz).

HRMS (ESI) m/z $[C_{40}H_{50}F_3N_8O_6]^+$ requires [M] 795.3800, found 795.3799.

Ethyl Janus-face morpholino-NDI (11b)

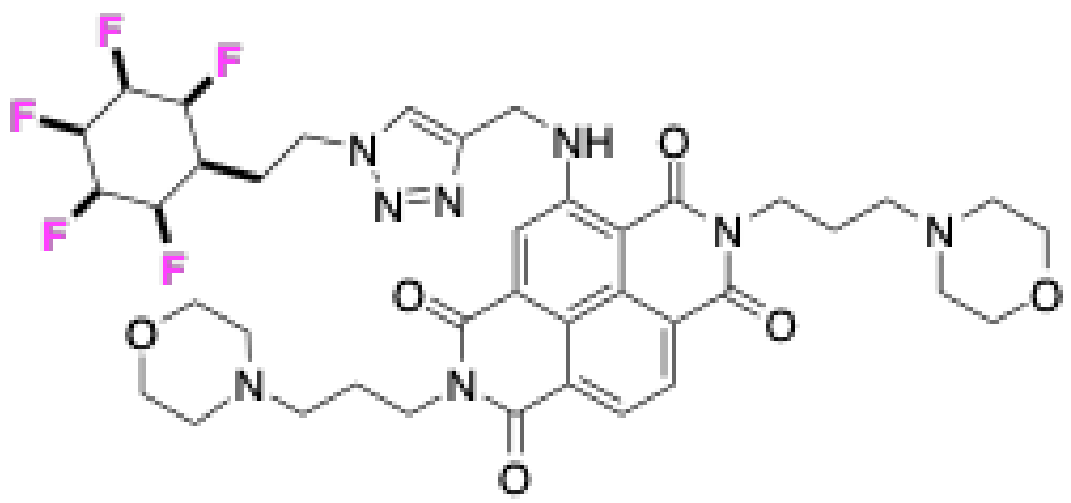

Thriazole **11b** was prepared according to General Procedure C from morpholino-NDI **21** (10 mg, 0.017 mmol), azide **26** (6.3 mg, 0.01 mmol), $CuSO_4.5H_2O$ (0.7 mg, 0.003 mmol) and sodium ascorbate (3.5 mg, 0.017 mmol). The product was purified by semi-prep HPLC (10-50% MeCN in H_2O with 0.1% TFA over 30 min, 2.5 mL/min, Synergi C18 250x10 mm, detection 254 nm, R_t 24.76 min) to give **11b** as a red semi-solid (2.0 mg, 20%).

^{1}H NMR (700 MHz, Acetone) δ 10.58 (t, *J* = 5.8 Hz, 1H, N**H**), 8.63 (d, *J* = 7.7 Hz, 1H, Ar**H**), 8.47 (s, 1H, Ar**H**), 8.34 (d, *J* = 7.7 Hz, 1H, Ar**H**), 8.20 (s, 1H, triazole **H**), 5.40 (d, *J* = 54.2 Hz, 2H, C**H**F), 5.13 (d, *J* = 49.9 Hz, 1H, C**H**F), 5.07 (d, *J* = 5.6 Hz, 2H, C**H**$_2$NH), 5.02 – 4.83 (m, 2H, C**H**F), 4.70 (t, *J* = 7.0 Hz, 2H, C**H**$_2$N-triazole), 4.25 (t, *J* = 7.2 Hz, 2H, NDI-NC**H**$_2$), 4.23 (t, *J* = 7.3 Hz, 2H, NDI-NC**H**$_2$), 3.44 – 3.36 (m, 8H, morpholine OC**H**$_2$), 2.46 (t, *J* = 6.5 Hz, 2H, C**H**$_2$N), 2.46 (t, *J* = 6.7 Hz, 2H, C**H**$_2$N), 2.43 (t, *J* = 7.3 Hz, 2H, CHC**H**$_2$), 2.33 (br s, 8H, morpholine C**H**$_2$N), 1.93 – 1.89 (m, 4H, C**H**$_2$), 1.81 – 1.74 (m, 1H, C**H**CH$_2$).

^{19}F{^{1}H} NMR (377 MHz, DMSO) δ -203.6 (t, *J* = 12.3 Hz), -211.6 (dt, *J* = 24.5, 6.1 Hz), -216.3 (tt, *J* = 25.1, 11.8 Hz).

HRMS (ESI) m/z $[C_{39}H_{46}F_5N_8O_6]^+$ requires [M] 817.3455, found 817.3463.

Ethoxy Janus-face dimethylamino-NDI (12a)

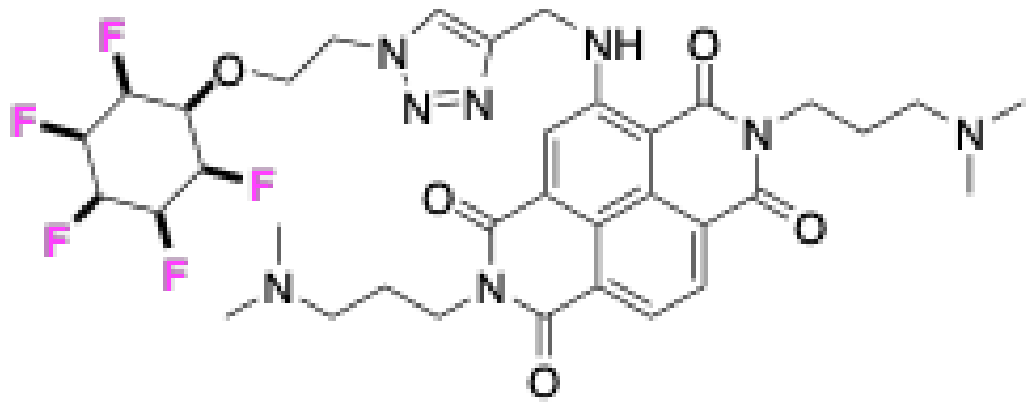

Triazole **12a** was prepared according to General Procedure C from dimethylamino-NDI **22** (3.7 mg, 0.005 mmol), azide **25** (2.6 mg, 0.01 mmol), $CuSO_4.5H_2O$ (0.2 mg, 0.001 mmol) and sodium ascorbate (1.0 mg, 0.005 mmol). The product was purified by semi-prep HPLC (25-60% MeCN in H_2O with 0.1% TFA over 30 min, Synergi C18 250x10 mm, 2.5 mL/min, detection 254 nm, R_t 13.45 min) to give **12a** as a red semi-solid (3.9 mg, 78%).

^{1}H NMR (700 MHz, MeOD) δ 8.65 (d, *J* = 7.6 Hz, 1H, Ar**H**), 8.40 (s, 1H, Ar**H**), 8.37 (d, *J* = 7.8 Hz, 1H, Ar**H**), 8.03 (s, 1H, triazole **H**), 5.11 – 4.99 (m, 2H, C**H**F), 5.03 (s, 2H, C**H**$_2$NH), 4.82 – 4.74 (m, 1H, C**H**F), 4.64 (br s, 2H, C**H**$_2$N-triazole), 4.56 – 4.36 (m, 2H, C**H**F), 4.32 (t, *J* = 6.7 Hz, 2H, OC**H**$_2$), 4.25 (t, *J* = 6.8 Hz, 2H, NDI-NC**H**$_2$), 3.98 (dd, *J* = 4.8, 4.8 Hz, 2H, NDI-NC**H**$_2$), 3.52 – 3.41 (m, 1H, C**H**O), 3.25 (t, *J* = 7.6 Hz, 4H, C**H**$_2$N), 2.93 (s, 6H, N(C**H**$_3$)$_2$), 2.92 (s, 6H, N(C**H**$_3$)$_2$), 2.23 – 2.16 (m, 4H, C**H**$_2$).

$^{19}F\{^{1}H\}$ NMR (377 MHz, MeOD) δ -212.2 – -212.3 (m), -216.7 – -216.9 (m), -217.8 – -218.1 (m).

HRMS (ESI) m/z $[C_{35}H_{42}F_5N_8O_5]^+$ requires [M] 749.3193, found 749.3196.

Ethyl Janus-face dimethylamine-NDI (12b)

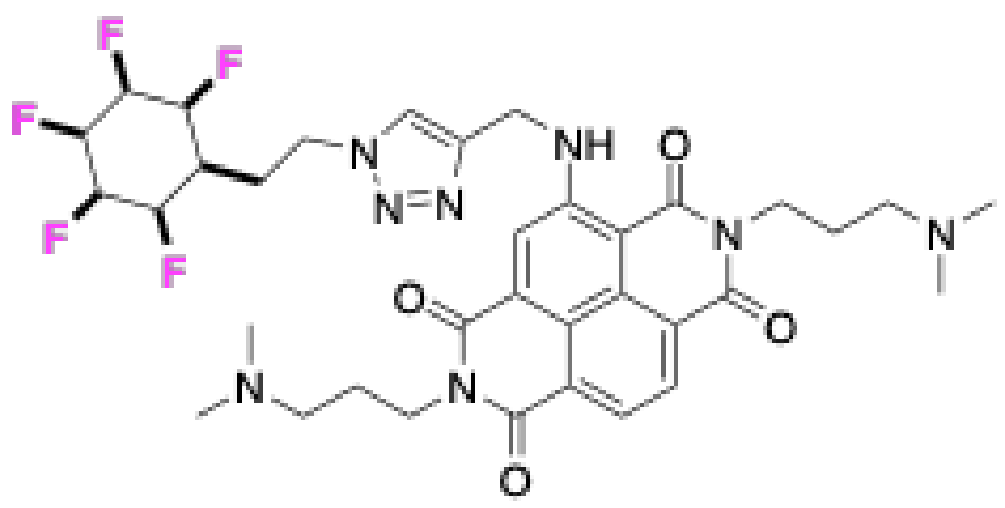

Triazole **12b** was prepared according to General Procedure C from dimethylamino-NDI **22** (9.7 mg, 0.014 mmol), azide **26** (4.9 mg, 0.020 mmol), $CuSO_4.5H_2O$ (0.7 mg, 0.002 mmol) and sodium ascorbate (3.5 mg, 0.014 mmol). The product was purified by prep HPLC (25-60% MeCN in H_2O with 0.1% TFA over 30 min, Synergi RP prep-HPLC column, 10 mL/min, detection 254 nm, R_t 18.28 min) to give **12b** as a red semi-solid (4.5 mg, 35%).

^{1}H NMR (700 MHz, MeOD) δ 8.67 (d, *J* = 7.8 Hz, 1H, Ar**H**), 8.40 (s, 1H, Ar**H**), 8.38 (d, *J* = 7.8 Hz, 1H, Ar**H**), 8.17 (s, 1H, triazole **H**), 5.22 (dt, *J* = 53.1, 8.4 Hz, 2H, C**H**F), 5.01 (s, 2H, C**H**$_2$NH), 4.63 (t, *J* = 7.2 Hz, 2H, C**H**$_2$N-triazole), 4.71 – 4.48 (m, 3H, C**H**F), 4.32 (t, *J* = 6.7 Hz, 2H, NDI-NC**H**$_2$), 4.25 (t, *J* = 6.8 Hz, 2H, NDI-NC**H**$_2$), 3.29 – 3.23 (m, 4H, C**H**$_2$N), 2.93 (s, 6H, N(C**H**$_3$)$_2$), 2.92 (s, 6H, N(C**H**$_3$)$_2$), 2.38 (q, *J* = 7.2 Hz, 1H, CHC**H**$_2$), 2.24 – 2.13 (m, 4H, C**H**$_2$), 1.97 – 1.83 (m, 1H, C**H**CH$_2$).

$^{19}F\{^{1}H\}$ NMR (377 MHz, D_2O) δ -204.8 (t, *J* = 10.4 Hz), -211.1 (dt, *J* = 27.2, 6.7 Hz), -216.9 – -217.2 (m).

HRMS (ESI) m/z $[C_{35}H_{42}F_5N_8O_4]^+$ requires [M] 733.3243, found 733.3249.

Propyl Janus-face dimethylamino-NDI (12c)

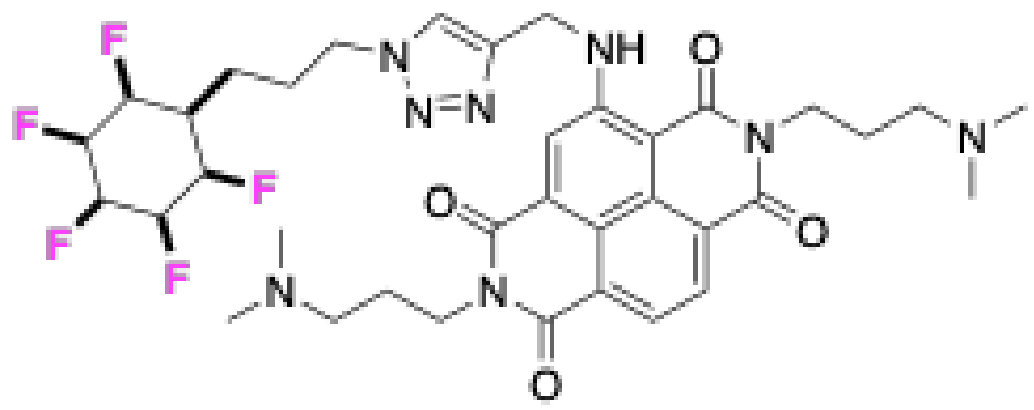

Triazole **12c** was prepared according to General Procedure C from dimethylamino-NDI **22** (9.7 mg, 0.014 mmol), azide **24** (5.2 mg, 0.020 mmol), $CuSO_4.5H_2O$ (0.7 mg, 0.002 mmol) and sodium ascorbate (3.5 mg, 0.014 mmol). The product was purified by prep HPLC (25-60% MeCN in H_2O with 0.1% TFA over 30 min, Synergi RP prep-HPLC column, 10 mL/min, detection 254 nm, R_t 17.77 min) to give **12c** as a red semi-solid (3.4 mg, 26%).

^{1}H NMR (400 MHz, D_2O) δ 8.34 (d, *J* = 7.8 Hz, 1H, Ar**H**), 8.08 (d, *J* = 7.9 Hz, 1H, Ar**H**), 8.02 (s, 1H, Ar**H**), 7.77 (s, 1H, triazole **H**), 5.20 – 5.06 (m, 2H, C**H**F), 4.95 (s, C**H**$_2$NH), 4.53 – 4.46 (m, 2H, C**H**$_2$N-triazole), 4.47 – 4.34 (m, 3H, C**H**F), 4.21 (t, *J* = 7.1 Hz, 2H, NDI-NC**H**$_2$), 4.11 (t, *J* = 7.0 Hz, 2H, NDI-NC**H**$_2$), 3.33 – 3.25 (m, 4H, C**H**$_2$N), 2.20 –2.10 (m, 4H, C**H**$_2$), 1.88 – 1.80 (m, 2H, C**H**$_2$), 1.65 (dt, *J* = 35.7, 7.5 Hz, 1H, C**H**CH$_2$), 1.14 – 1.05 (m, 2H, C**H**$_2$).

^{19}F{^{1}H} NMR (377 MHz, D_2O) δ -204.8 (t, *J* = 9.0 Hz), -213.1 – -213.3 (m), -217.0 (ddd, *J* = 39.1, 27.1, 11.8 Hz).

HRMS (ESI) m/z $[C_{36}H_{44}F_5N_8O_4]^+$ requires [M] 747.3400, found 747.3411.

NMR Spectra of final products

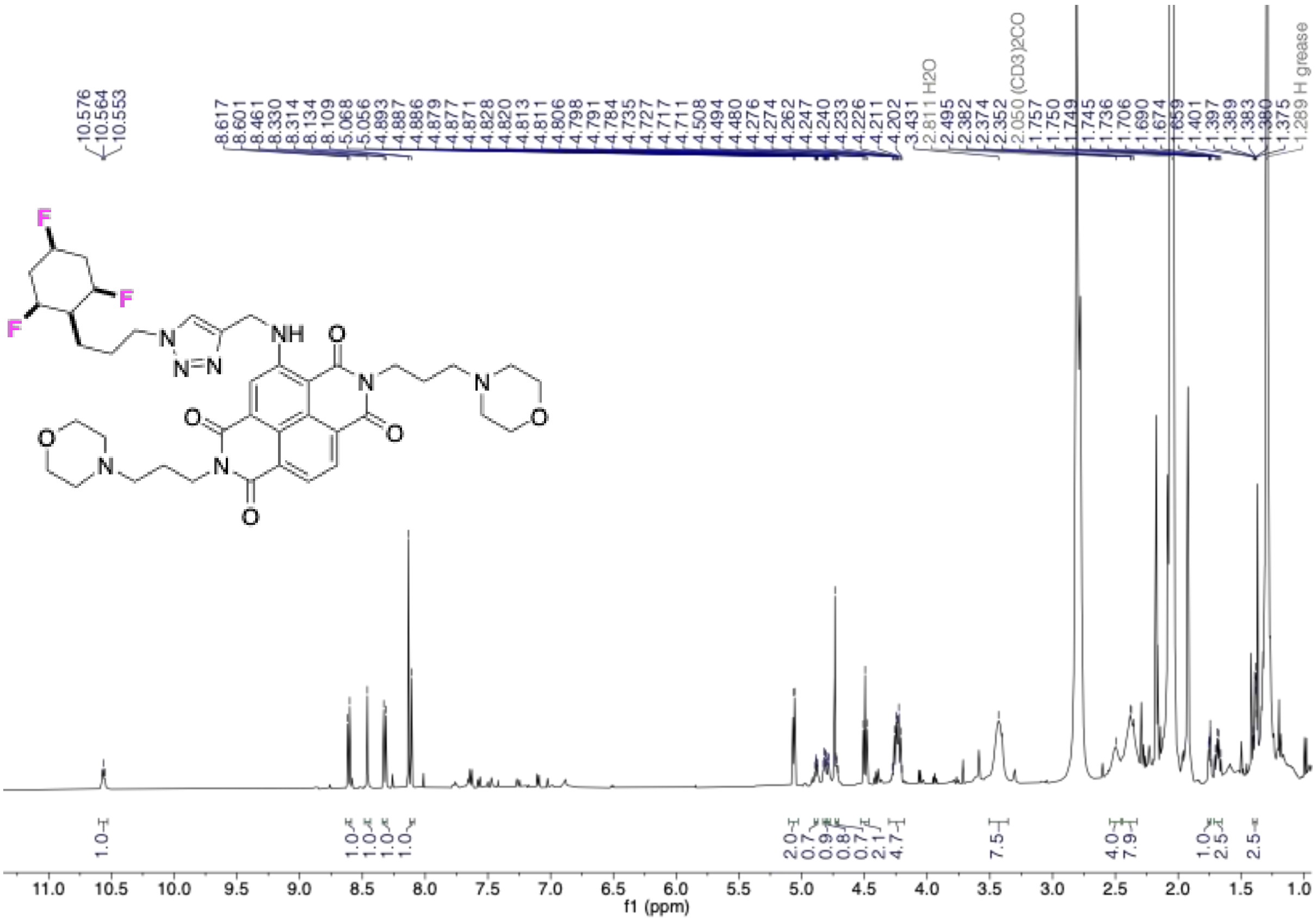

Compound 11a ^{1}H NMR (500 MHz, Acetone)

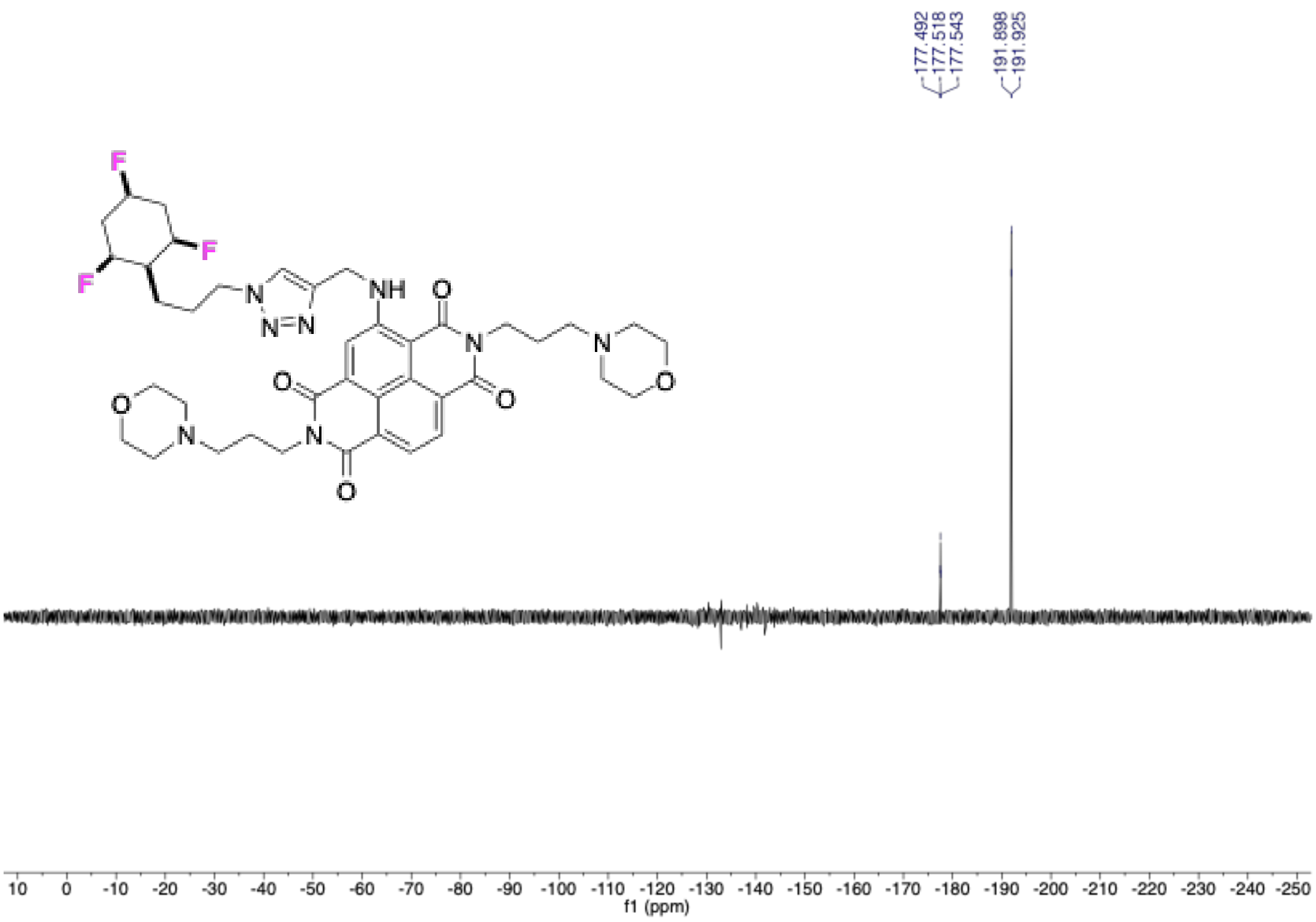

Compound 11a $^{19}F\{^{1}H\}$ NMR (470 MHz, Acetone)

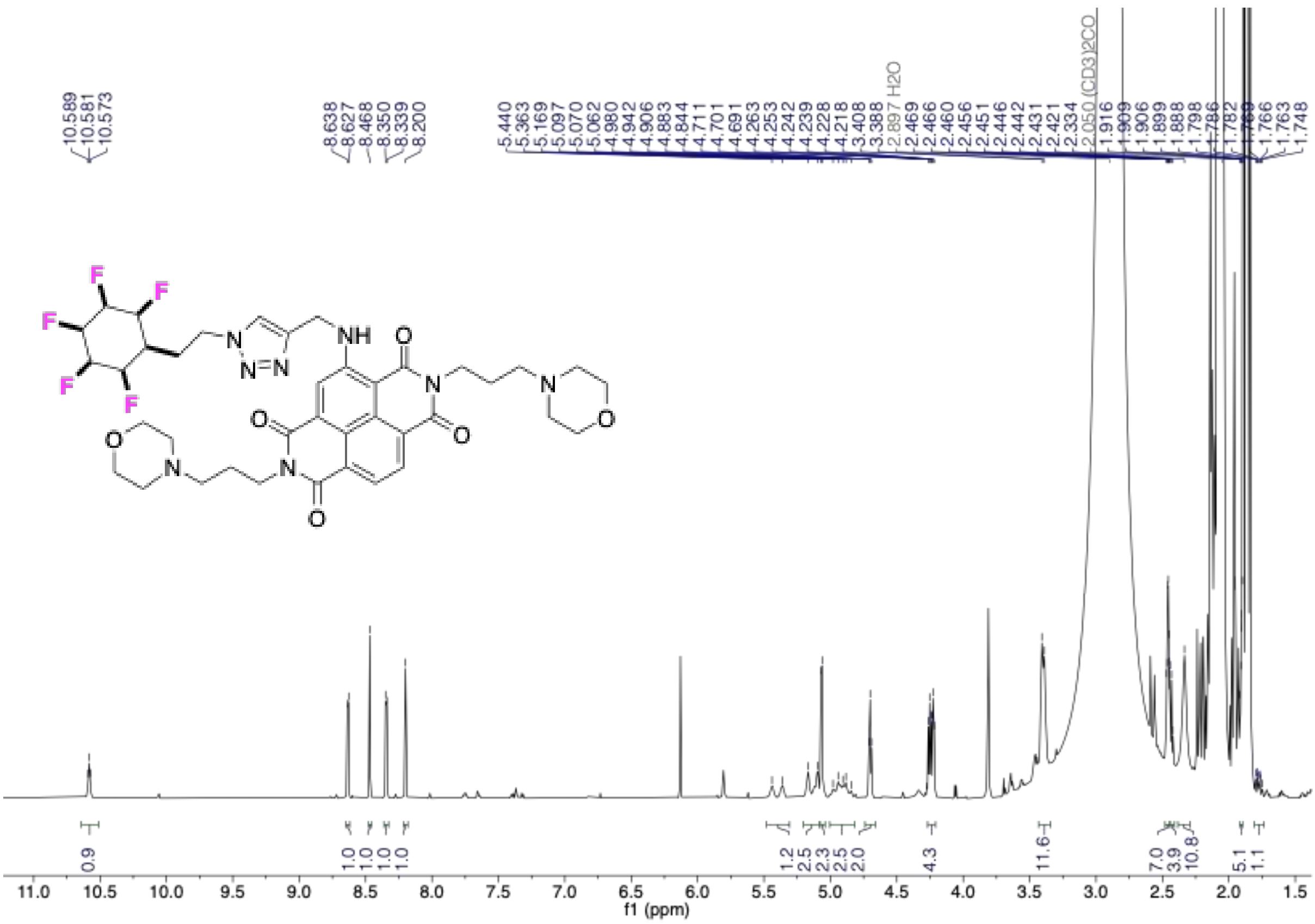

Compound 11b ^{1}H NMR (700 MHz, Acetone)

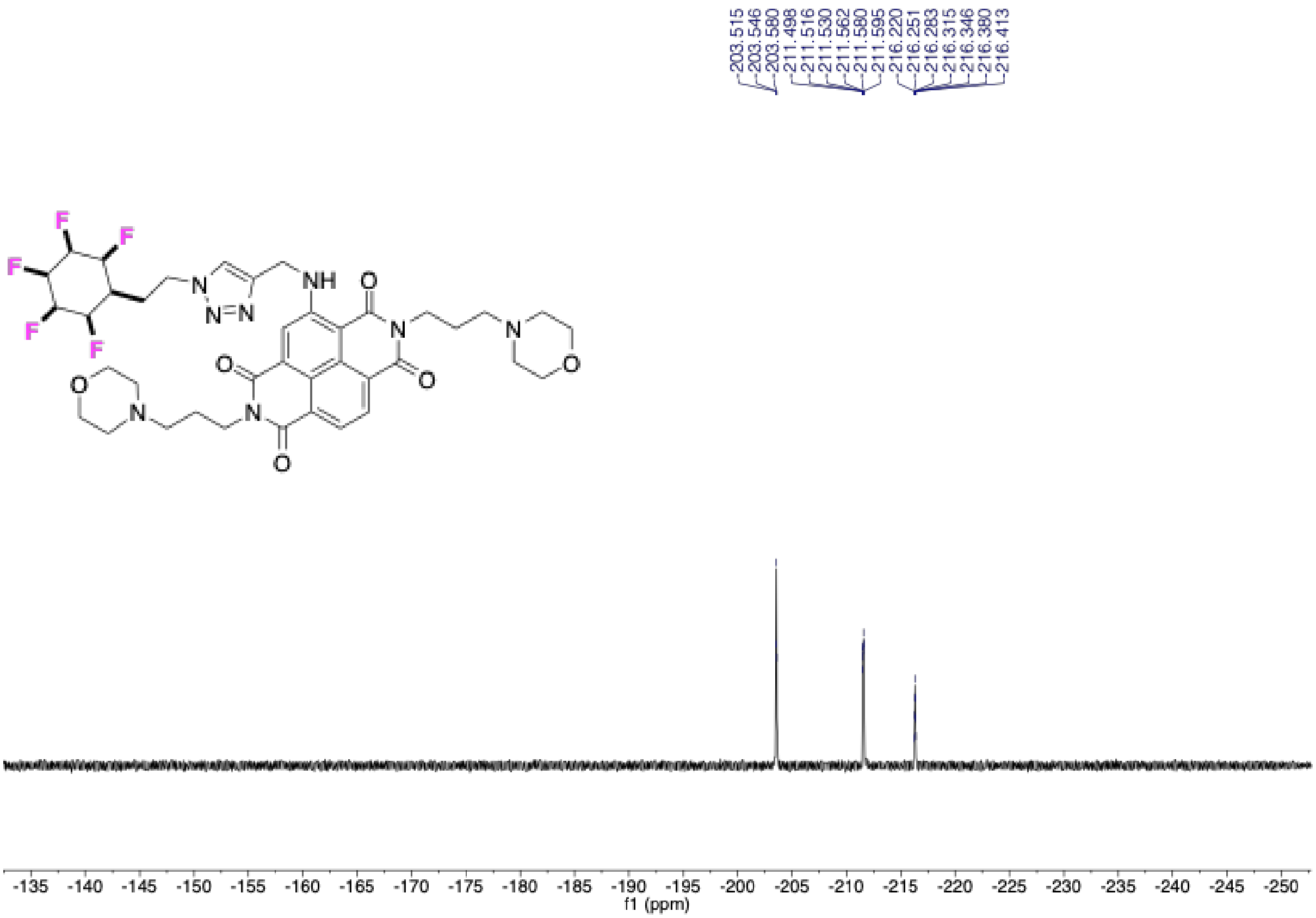

Compound 11b ^{19}F{^{1}H} NMR (377 MHz, DMSO)

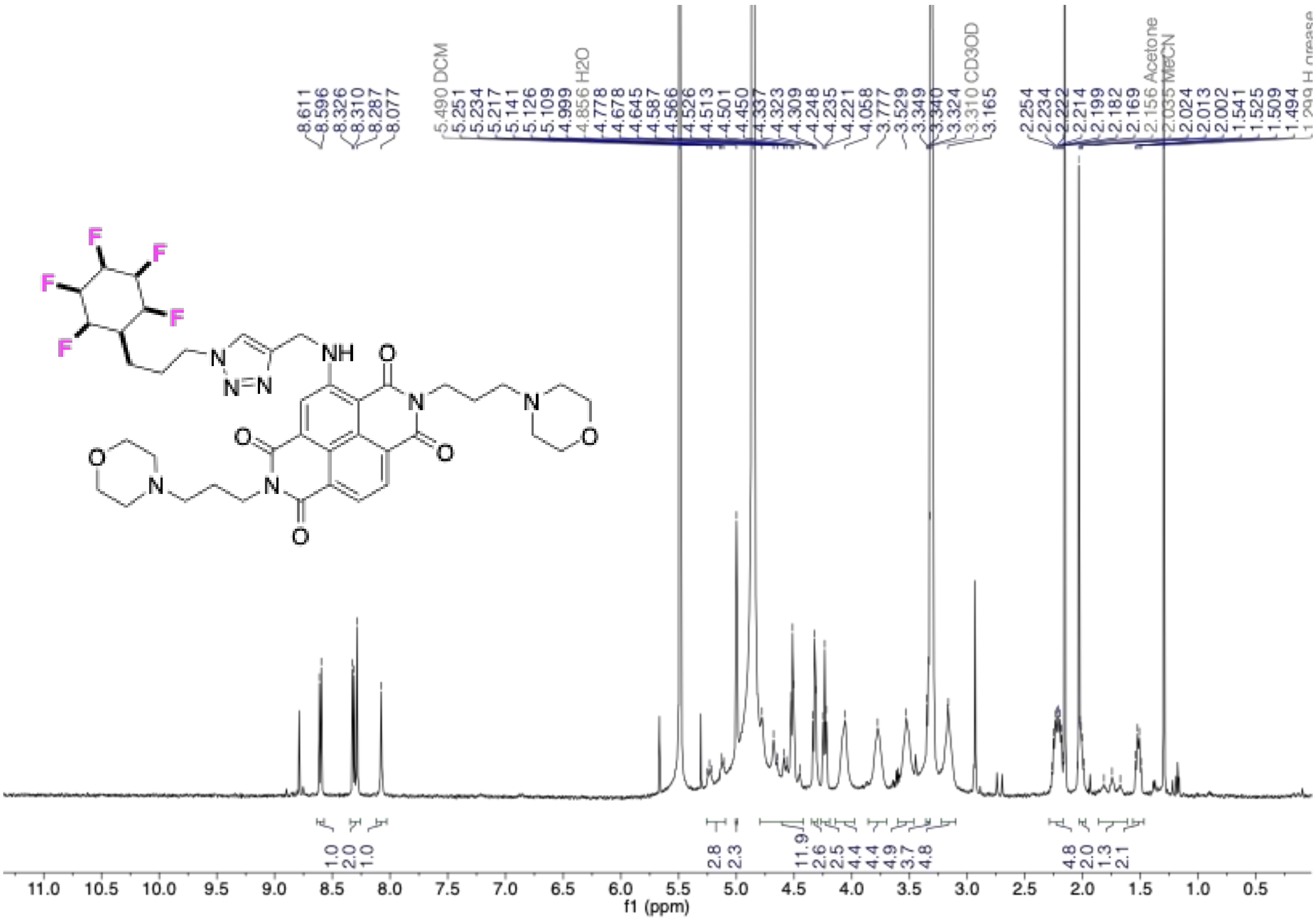

Compound 11c ^{1}H NMR (500 MHz, MeOD)

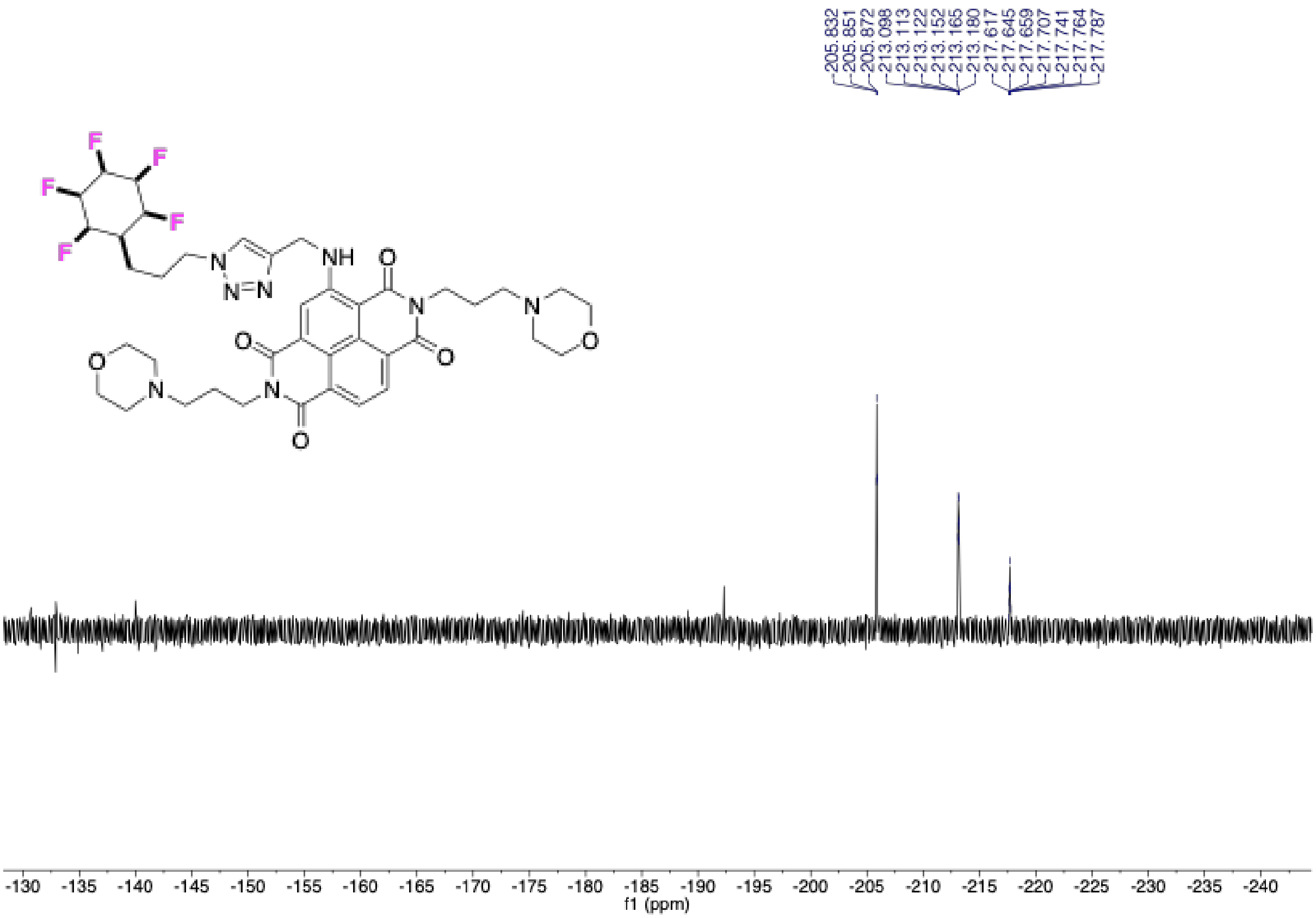

Compound 11c ^{19}F{^{1}H} NMR (470 MHz, MeOD)

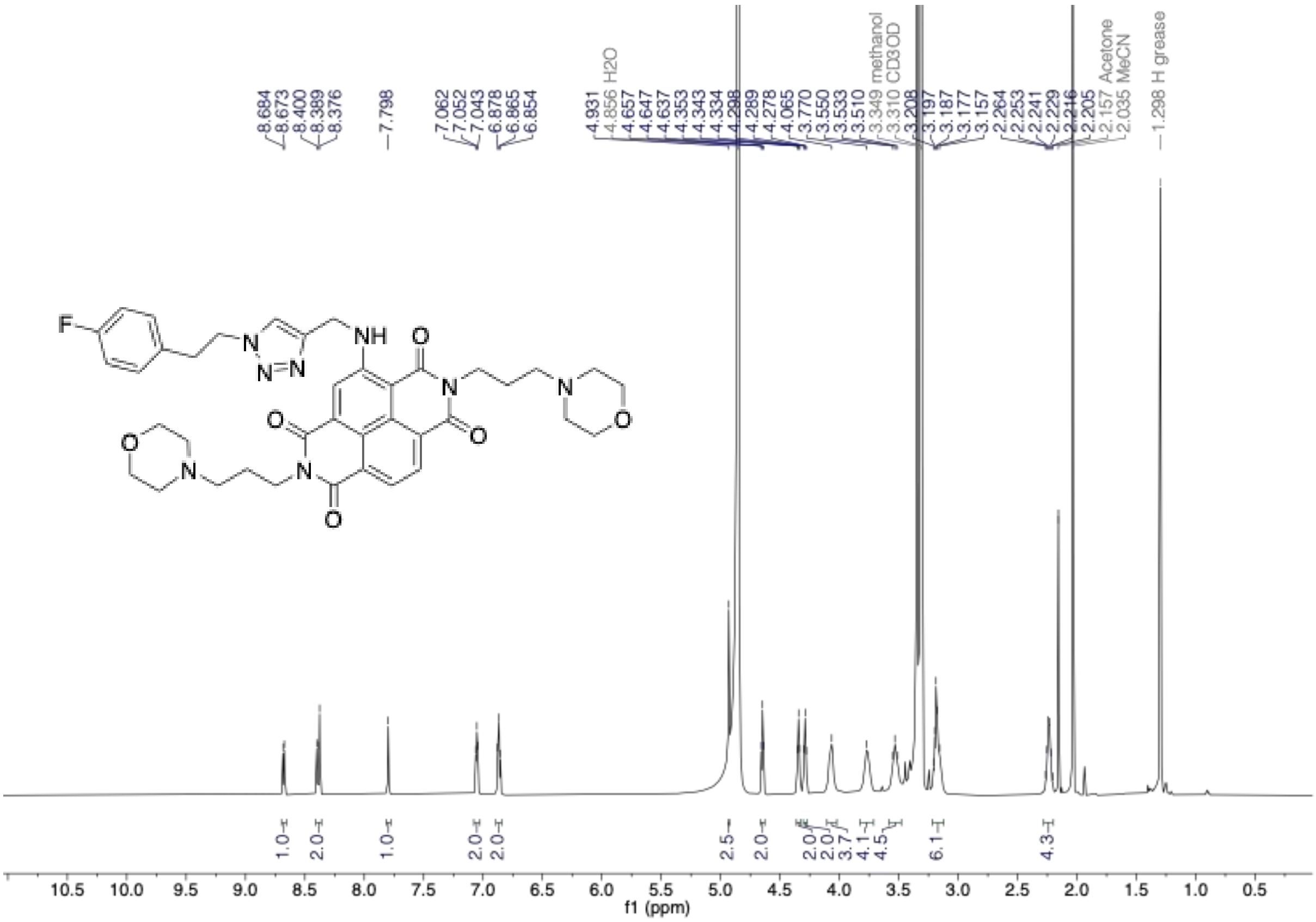

Compound 11d ^{1}H NMR (700 MHz, MeOD)

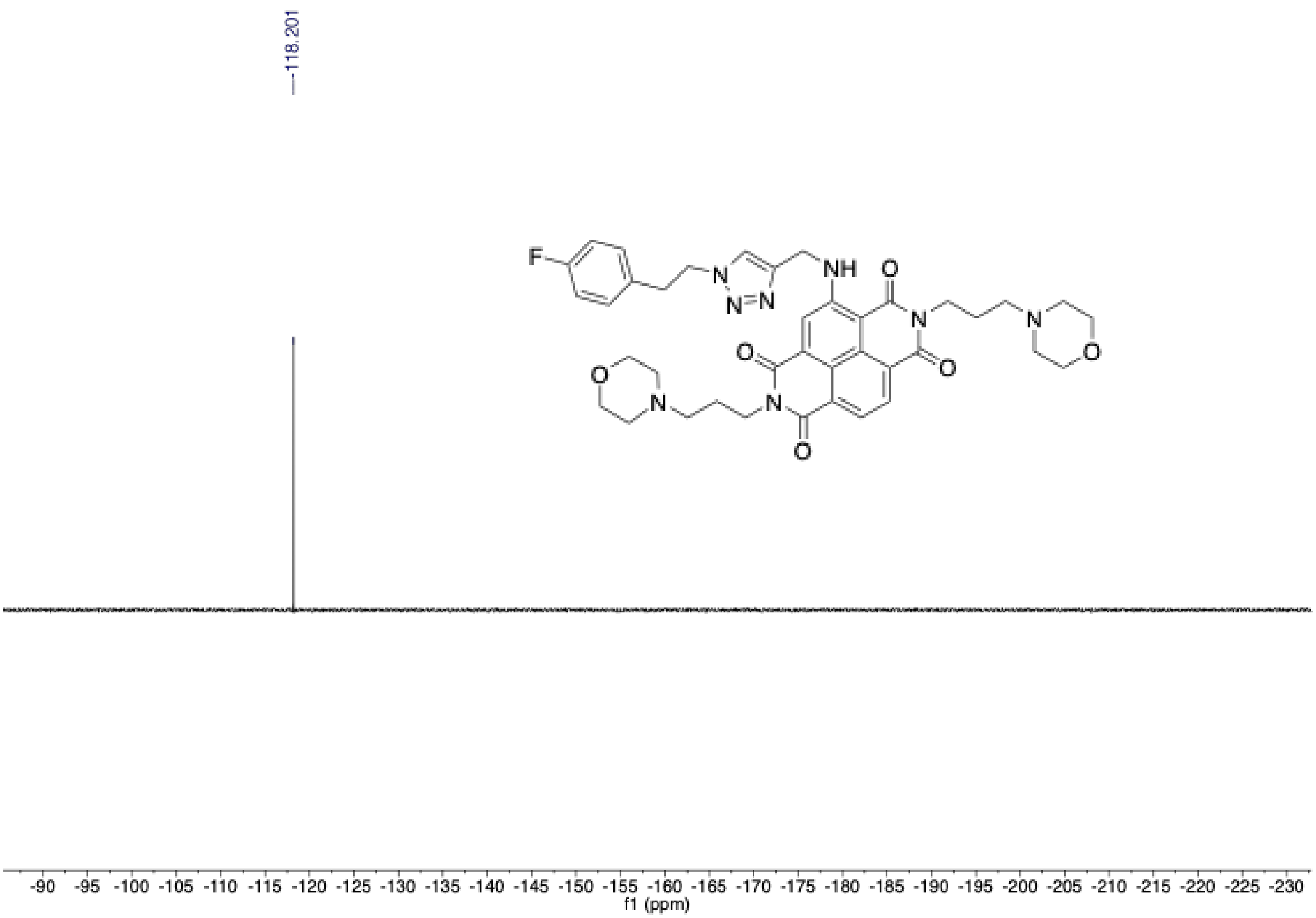

Compound 11d ^{19}F{^{1}H} NMR (377 MHz, MeOD)

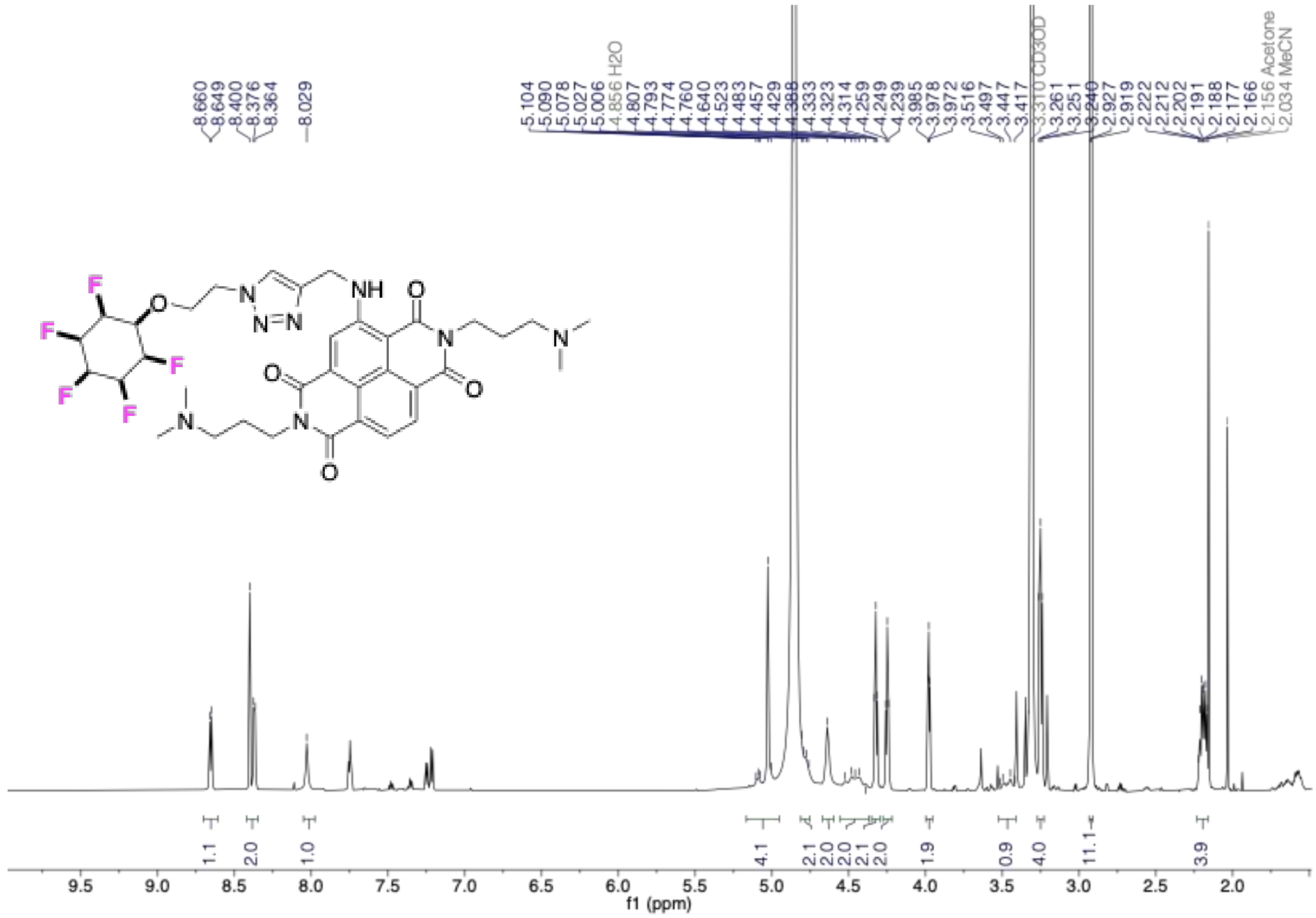

Compound 12a ^{1}H NMR (700 MHz, MeOD)

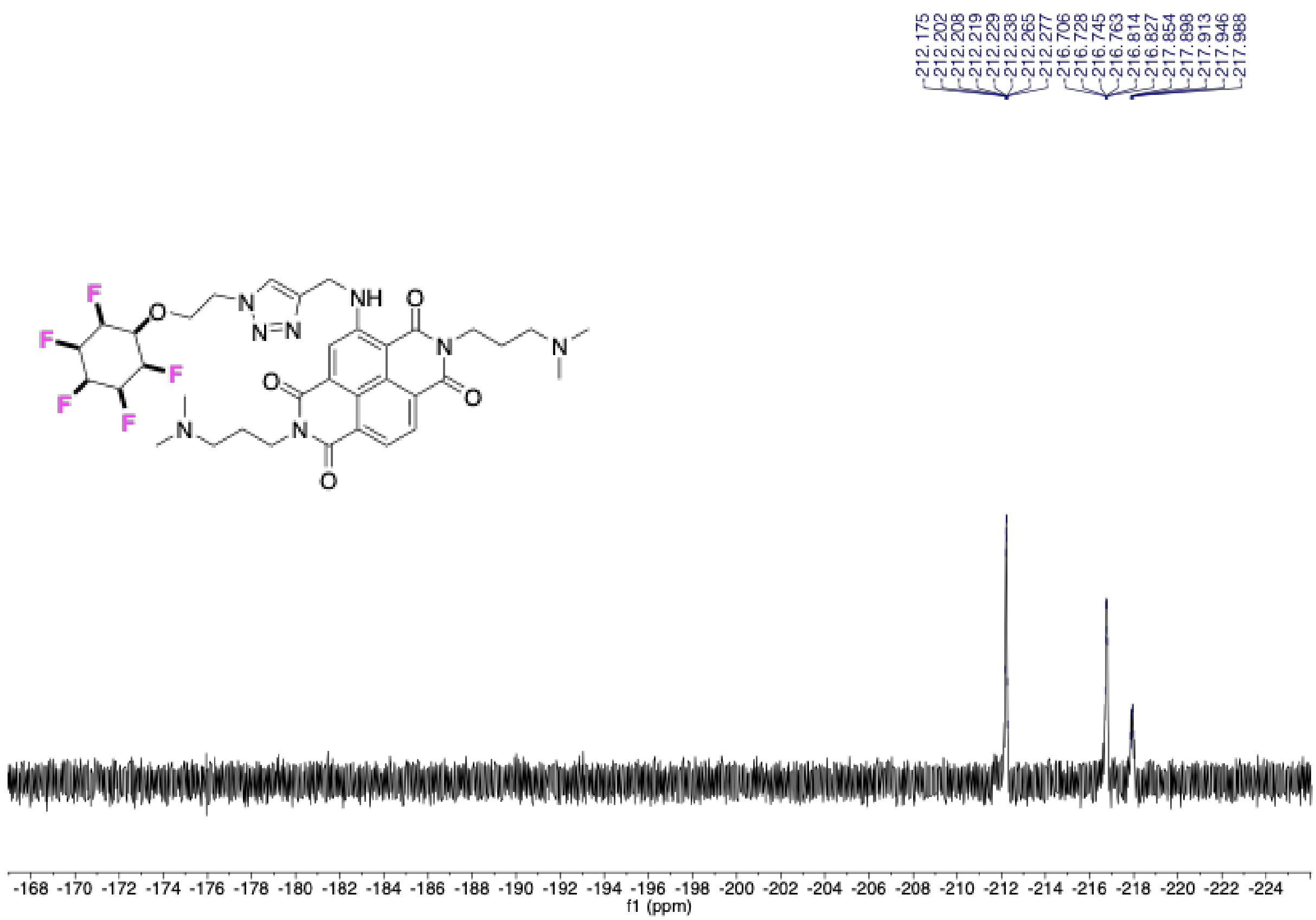

Compound 12a ^{19}F{^{1}H} NMR (377 MHz, MeOD)

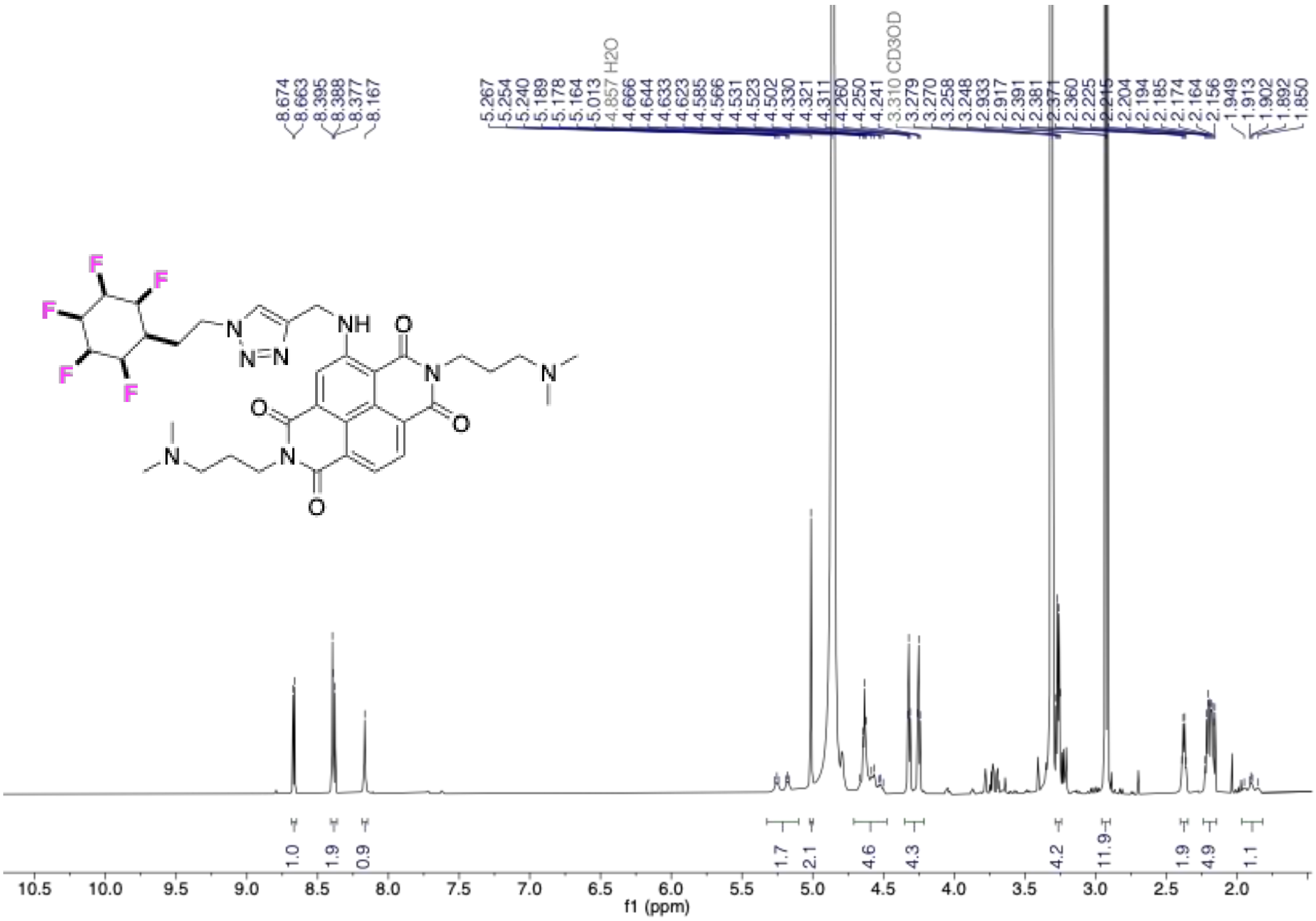

Compound 12b ^{1}H NMR (700 MHz, MeOD)

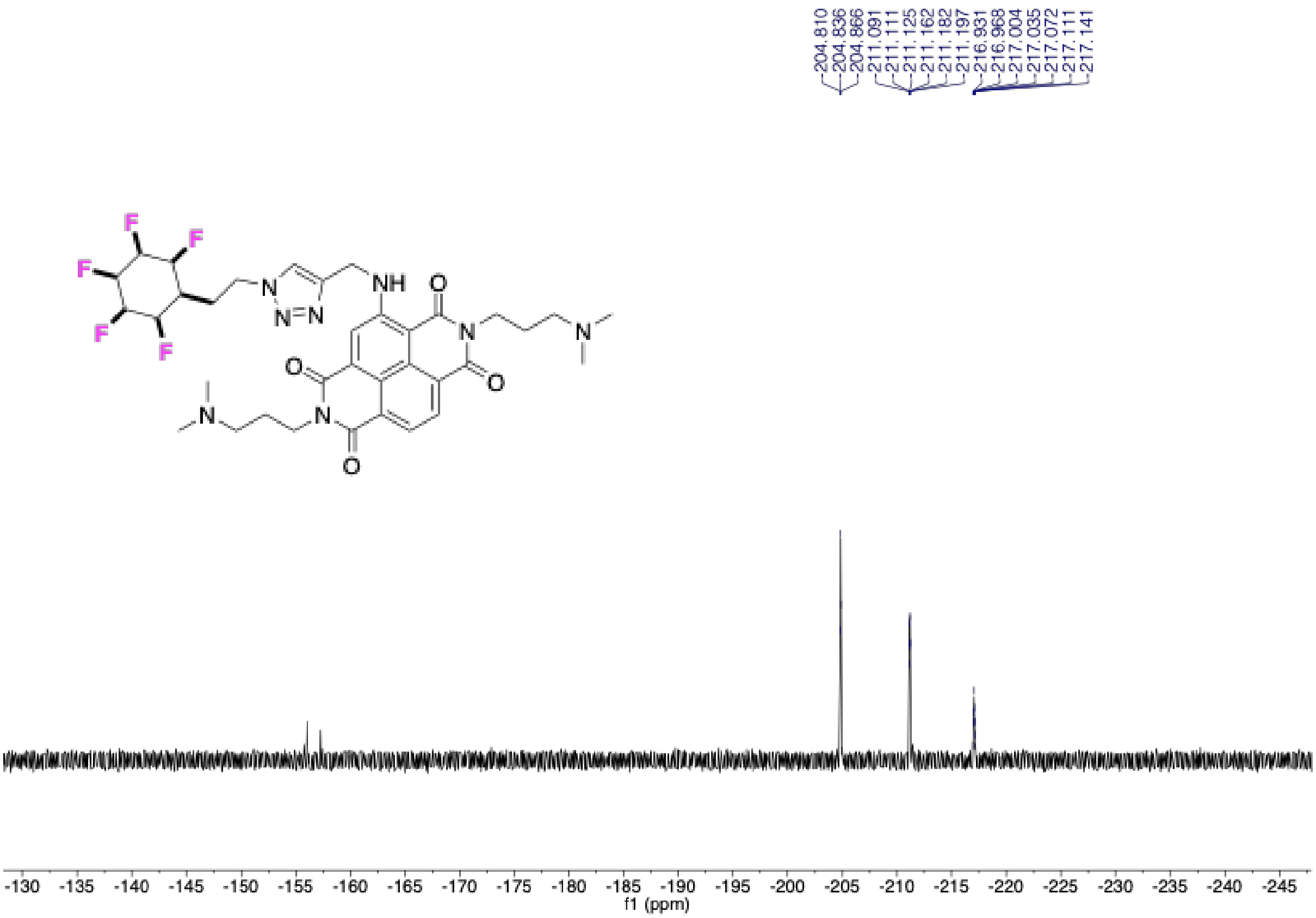

Compound 12b $^{19}F\{^{1}H\}$ NMR (377 MHz, D_2O)

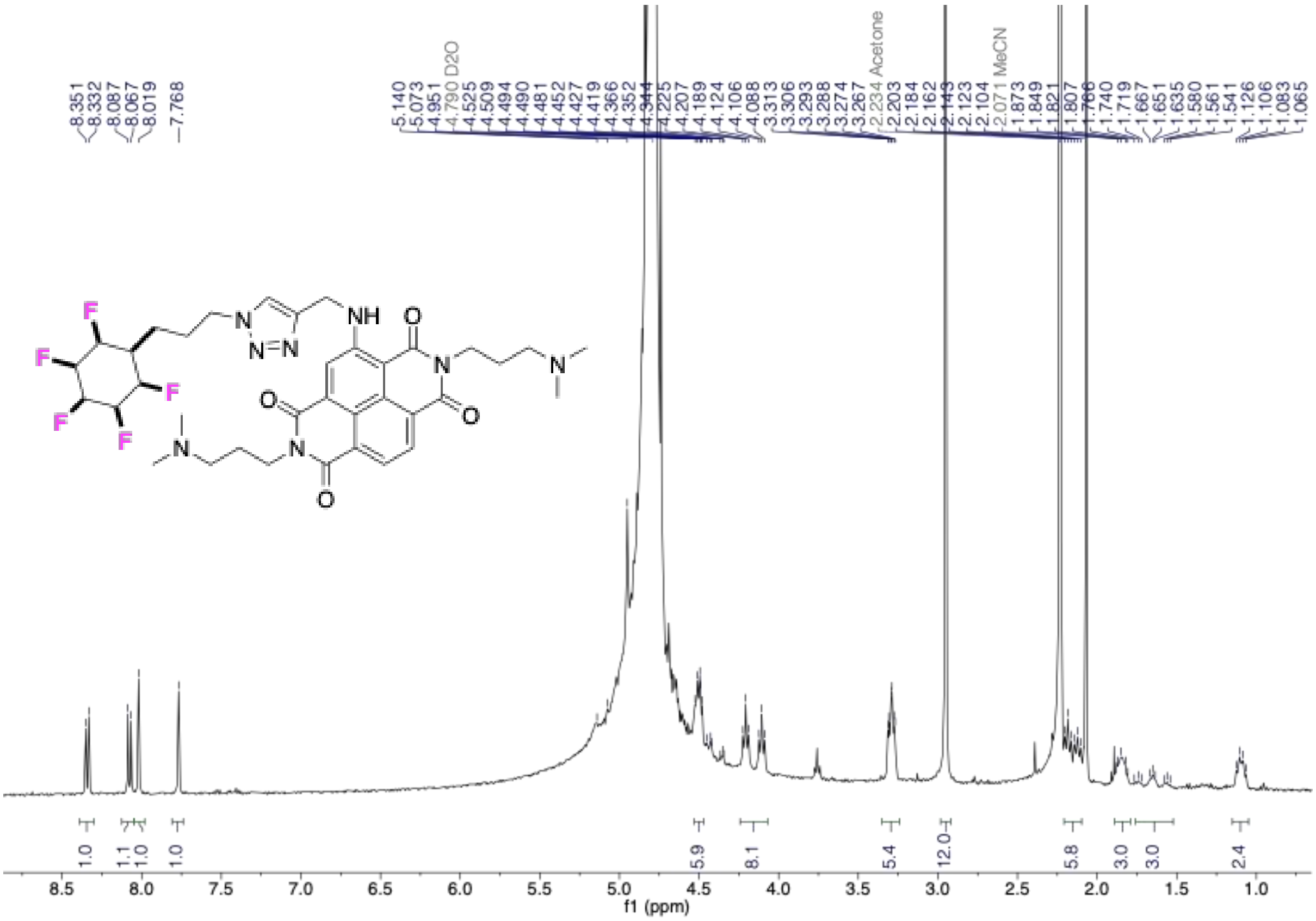

Compound 12c ^{1}H NMR (400 MHz, D_2O)

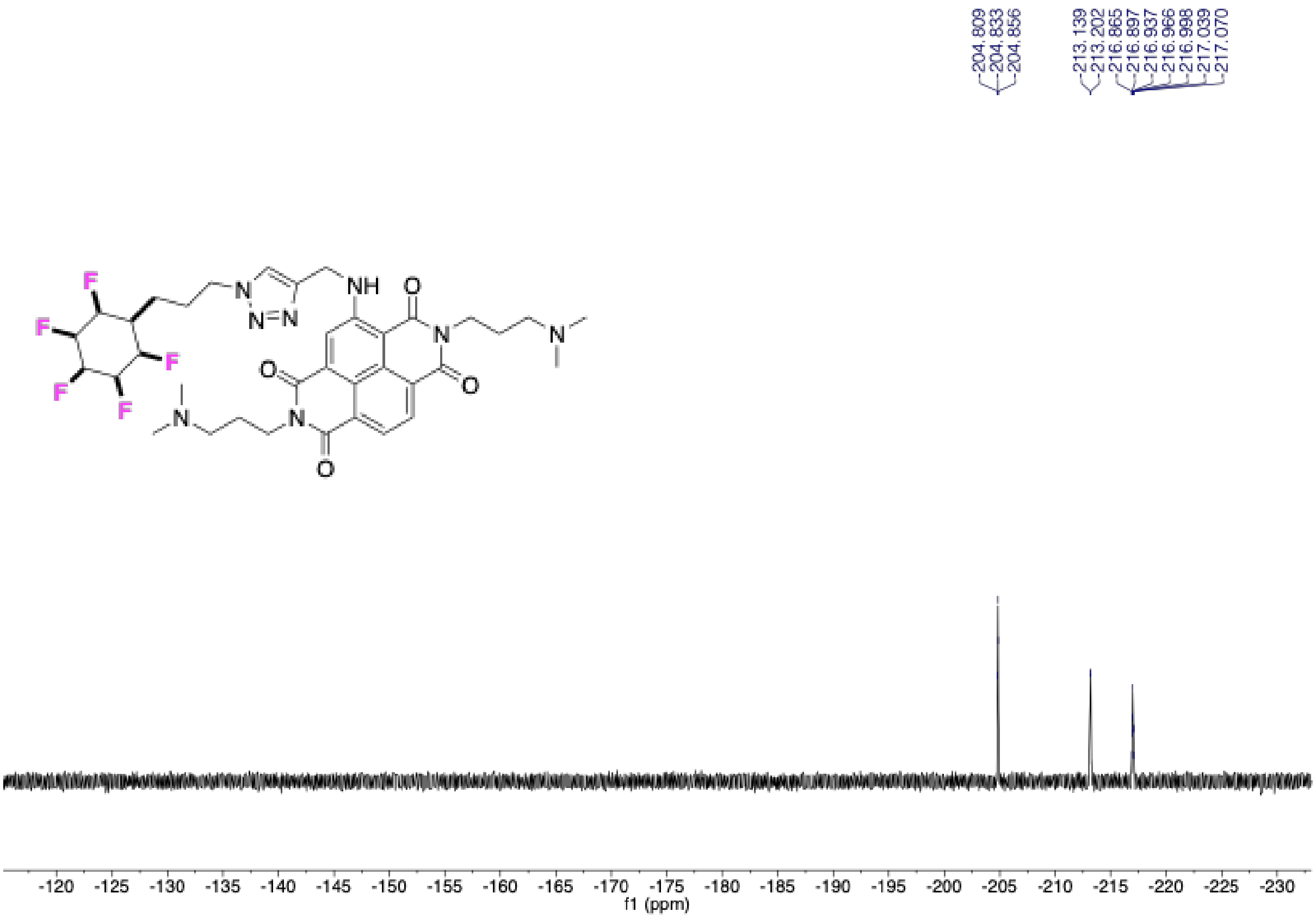

Compound 12c $^{19}F\{^{1}H\}$ NMR (377 MHz, D_2O)

Oligonucleotides

Oligonucleotides were purchased from Eurogentec and were HPLC purified. Solid DNA samples were initially dissolved as stock solutions in purified water (100 μM for labelled oligonucleotides); further dilutions were carried out in 10 mM sodium cacodylate buffer containing 10 mM KCl at pH 7.4. Samples were thermally annealed in a heat block at 95 °C for 5 minutes and cooled slowly to room temperature overnight. Stock solutions of the ligands were prepared at 10 mM in DMSO and were stored at −20 °C; Subsequent ligand dilutions were prepared in DMSO to the required concentrations.

FRET Melting Assay

Assessment of the ligand-induced change in melting temperature was performed using a fluorescence resonance energy transfer (FRET) DNA melting-based assay.[12]

The labelled oligonucleotides with a donor fluorophore FAM (6-carboxyfluorescein) and acceptor fluorophore TAMRA (6-carboxytetramethylrhodamine) were prepared as a 220 nM solution in 10 mM sodium cacodylate buffer containing 10 mM KCl at pH 7.4 and then thermally annealed. Plates were prepared by combining 18 μL of DNA solution with the respective compounds to give a final DNA concentration of 0.2 μM and final compound concentrations of 1, 5, 10, 20 and 50 μM. Control samples for each run were prepared with the same quantity of DMSO with the DNA in buffer.

Fluorescence melting curves were determined using an Applied Biosystems 7500 Real-Time PCR System, using a total volume of 20 μL per well. Samples were held at 25 °C for 5 minutes and then ramped to 95 °C in increments of 1 °C, holding the temperature at each step for 1 minute. Measurements were made with excitation at 470 nm and detection at 510 nm. The DNA sequences used were the G-quadruplex-forming sequence from the human telomere, hTeloGFRET (5′-FAM-d[GGG-TTA-GGG-TTA-GGG-TTA-GGG]-TAMRA-3′) and double helical DNA, DSFRET FAM-d(TAT-AGC-TAT-A-HEG(18)-TAT-AGC-TAT-A)-TAMRA-3′), all of which were measured in 10 mM sodium cacodylate buffer containing 10 mM KCl at pH 7.4. Final analysis of the data was carried out using Excel and Origin. Tm values were determined using the first derivative of the melting curves.

FRET melting experiments

FRET melting experiments were performed for compounds **11a–b** and **12a–c** against the human telomeric G-quadruplex (hTeloG) and a double-stranded DNA control over a ligand concentration range of 1–50 µM. Each experiment was performed independently twice. The individual melting temperatures are presented below. The values reported in Tables 1 and 2 of the main manuscript correspond to measurements obtained at 20 µM ligand concentration and are presented as the mean ± standard deviation of two independent experiments.

Table S1. FRET melting temperatures of hTeloG and dsDNA in the presence of increasing concentrations of compound 11a.

**Experiment**	**DNA (0.2 µM)**	**Transition (°C)**	**Control**	**Compound 11a concentration (µM)**				
				**1**	**5**	**10**	**20**	**50**
1	dsDNA	T_m	51	51	51	51	51	51
	hTeloG	T_m	51	49	50	50	51	54
2	dsDNA	T_m	50	51	51	51	51	51
	hTeloG	T_m	48	48	49	49	49	51

Figure S1. Representative FRET melting analysis of dsDNA and hTeloG in the presence of increasing concentrations of compound 11a. (A) Fluorescence melting curves of dsDNA. (B) Corresponding first-derivative curves of dsDNA. (C) Fluorescence melting curves of hTeloG. (D) Corresponding first-derivative curves of hTeloG shown over the full y-axis range.

A)

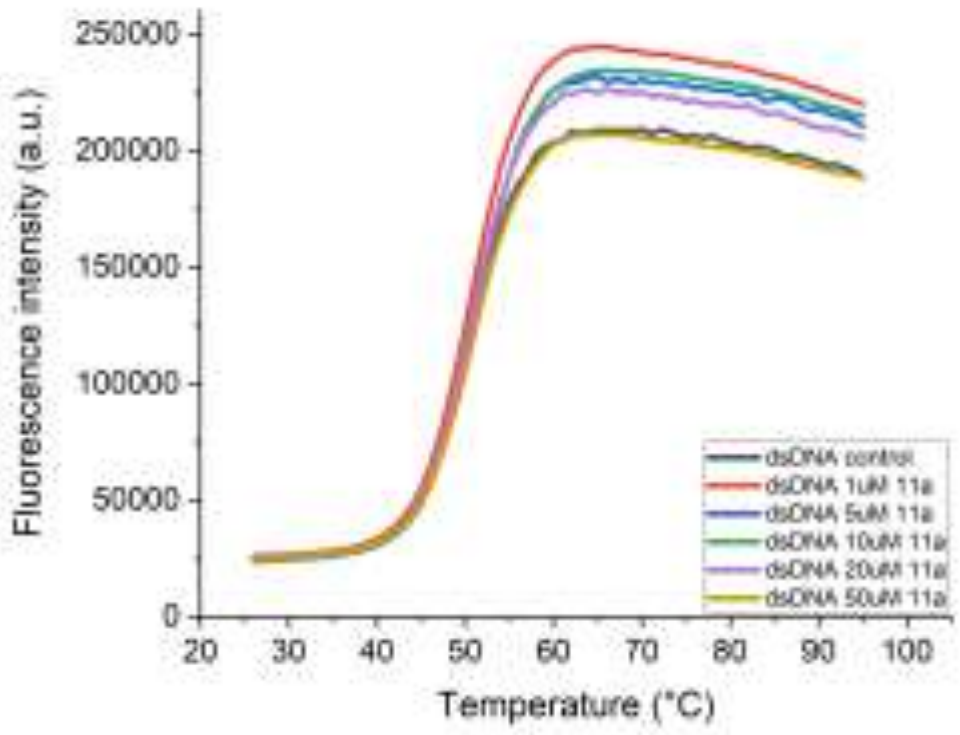

B)

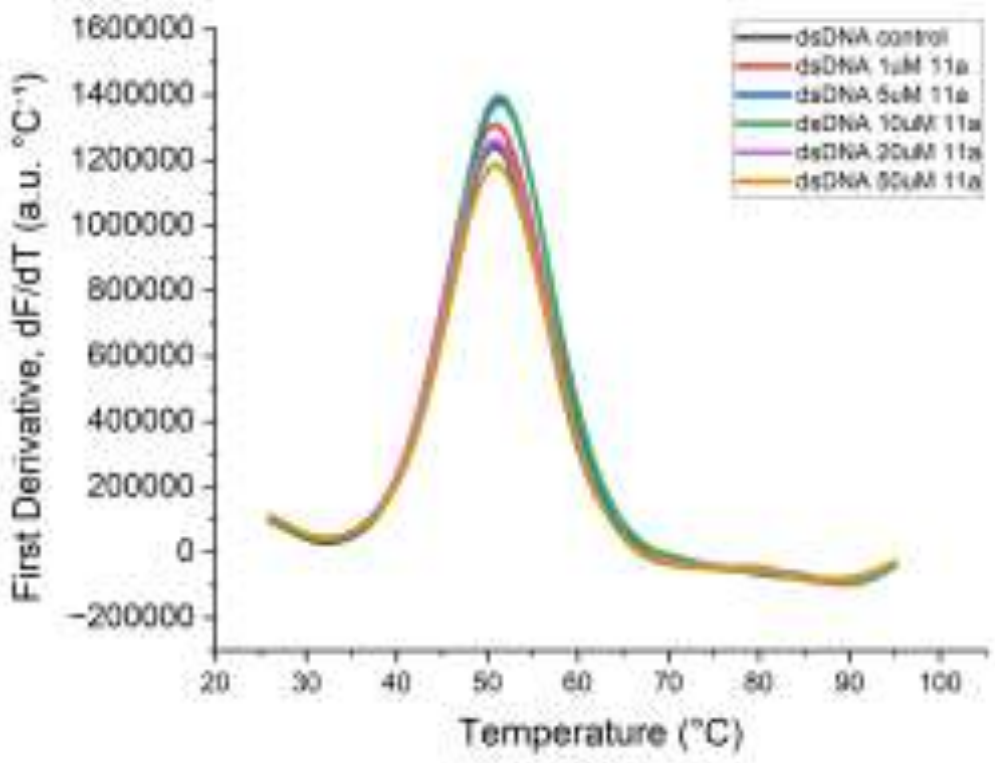

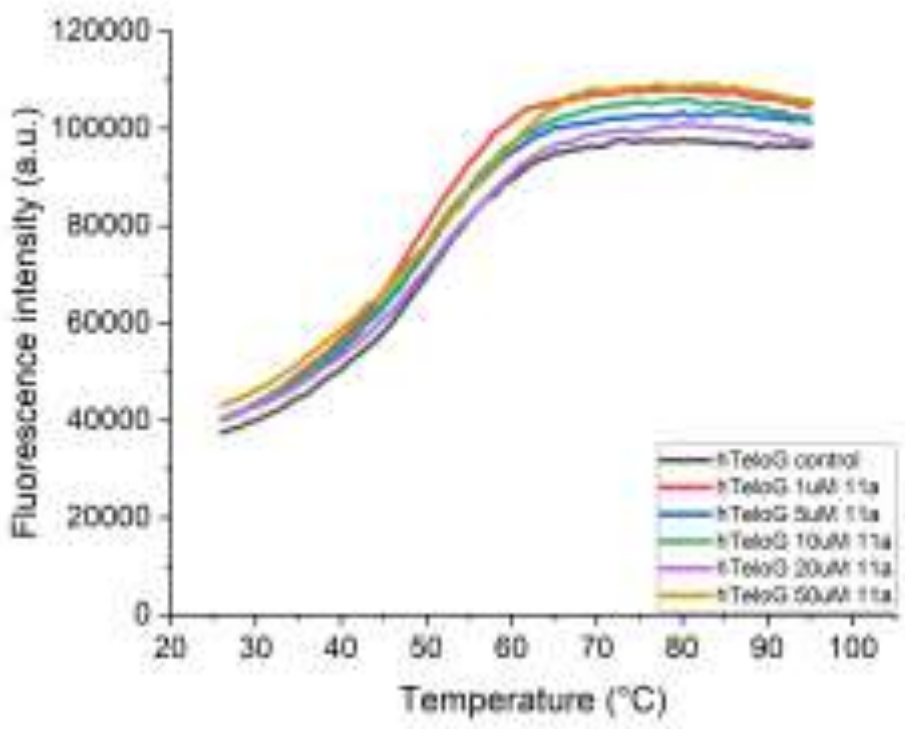

C)

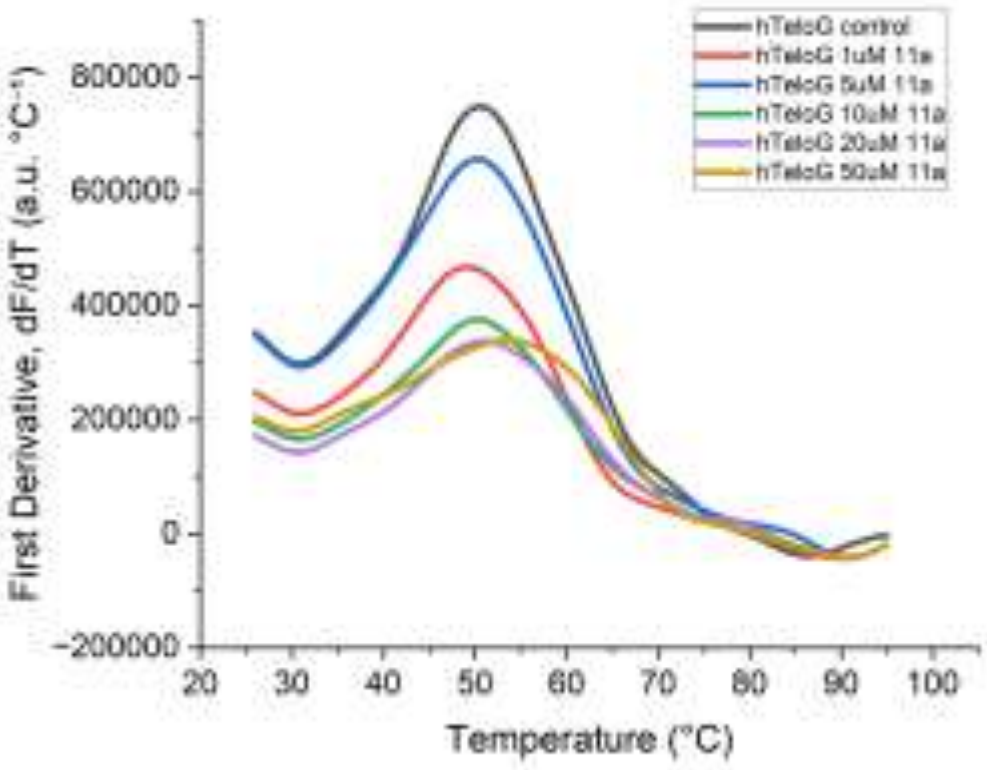

D)

Table S2. FRET melting temperatures of hTeloG and dsDNA in the presence of increasing concentrations of compound 11b.

Experiment	DNA (0.2 µM)	Transition (°C)	Control	Compound 11b concentration (µM)				
				1	5	10	20	50
1	dsDNA	T_m	50	51	51	51	53	54
	hTeloG	T_m1	50	N/A	N/A	N/A	N/A	43
		T_m2		50	52	52	55	60
2	dsDNA	T_m	50	50	51	51	51	51
	hTeloG	T_m1	49	N/A	N/A	N/A	N/A	42
		T_m2		49	50	49	54	59

Figure S2. Representative FRET melting analysis of dsDNA and hTeloG in the presence of increasing concentrations of compound 11b. (A) Fluorescence melting curves of dsDNA. (B) Corresponding first-derivative curves of dsDNA. (C) Fluorescence melting curves of hTeloG. (D) Corresponding first-derivative curves of hTeloG shown over the full y-axis range. (E) Enlarged view of the hTeloG first-derivative curves using a reduced y-axis scale to visualise the lower-intensity transitions. Compound concentrations were 0, 1, 5, 10, 20, and 50 µM. Representative data from one of two independent experiments are shown.

A)

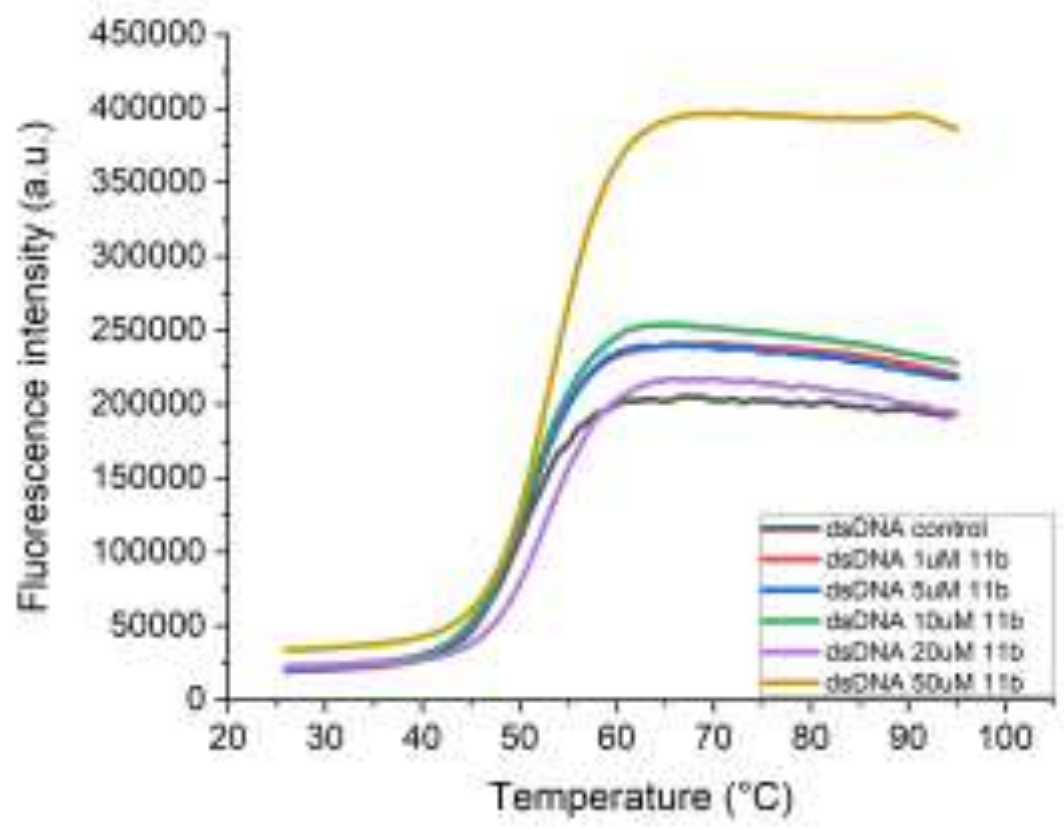

B)

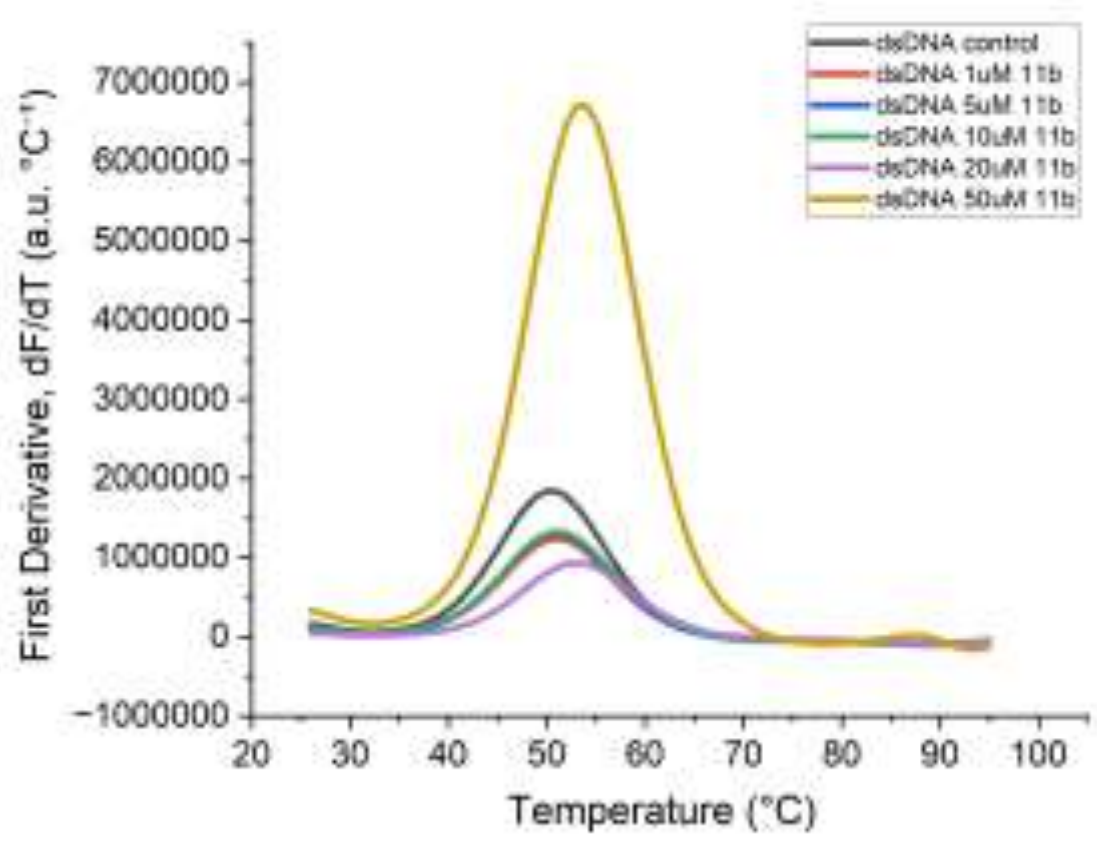

C)

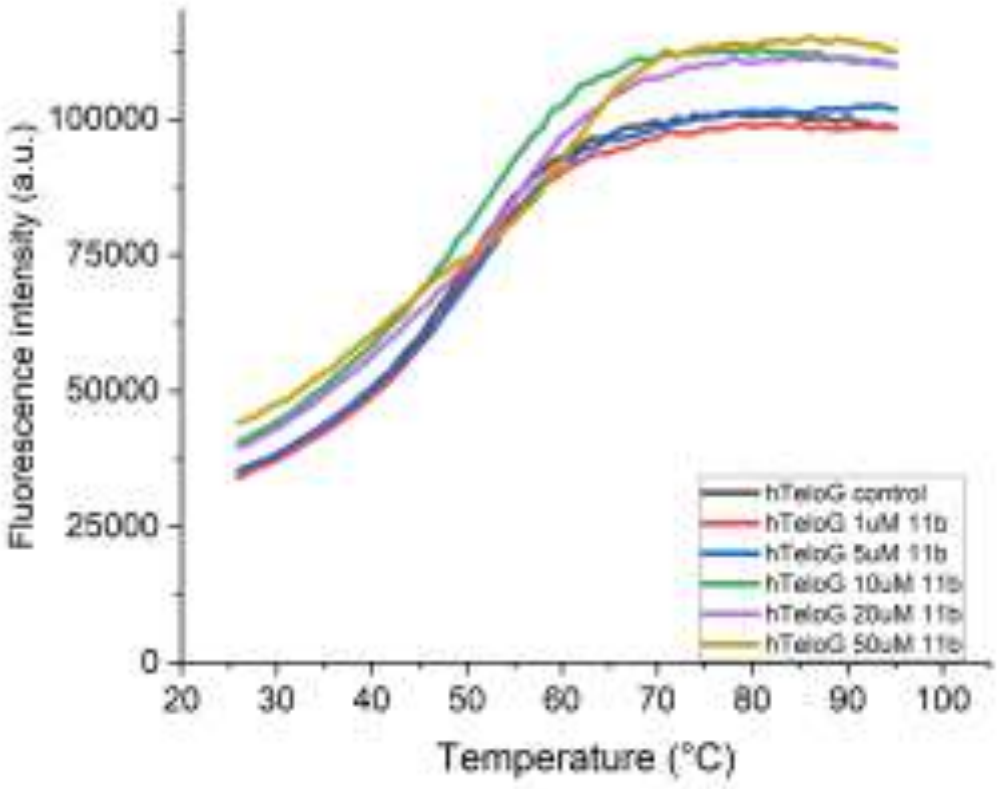
Fluorescence intensity (a.u.)
Temperature (°C)
hTeloG control
hTeloG 1uM 11b
hTeloG 5uM 11b
hTeloG 10uM 11b
hTeloG 20uM 11b
hTeloG 50uM 11b

D)

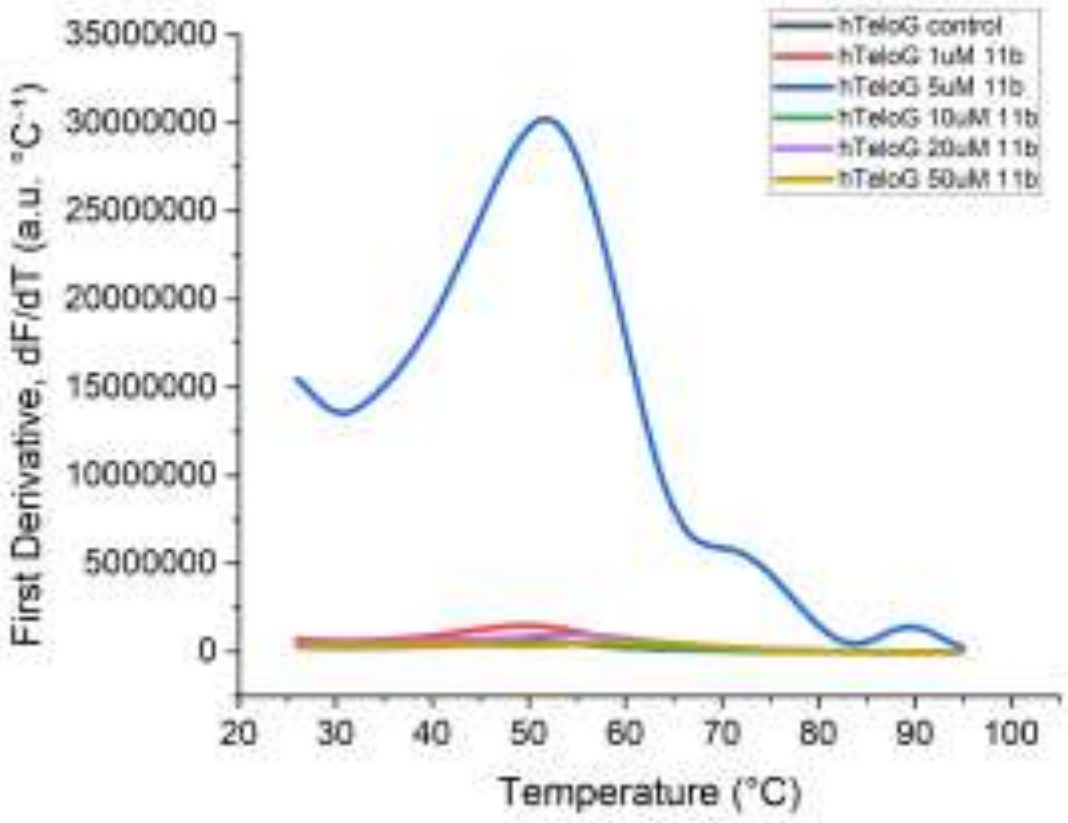
First Derivative, dF/dT (a.u. °C⁻¹)
Temperature (°C)
hTeloG control
hTeloG 1uM 11b
hTeloG 5uM 11b
hTeloG 10uM 11b
hTeloG 20uM 11b
hTeloG 50uM 11b

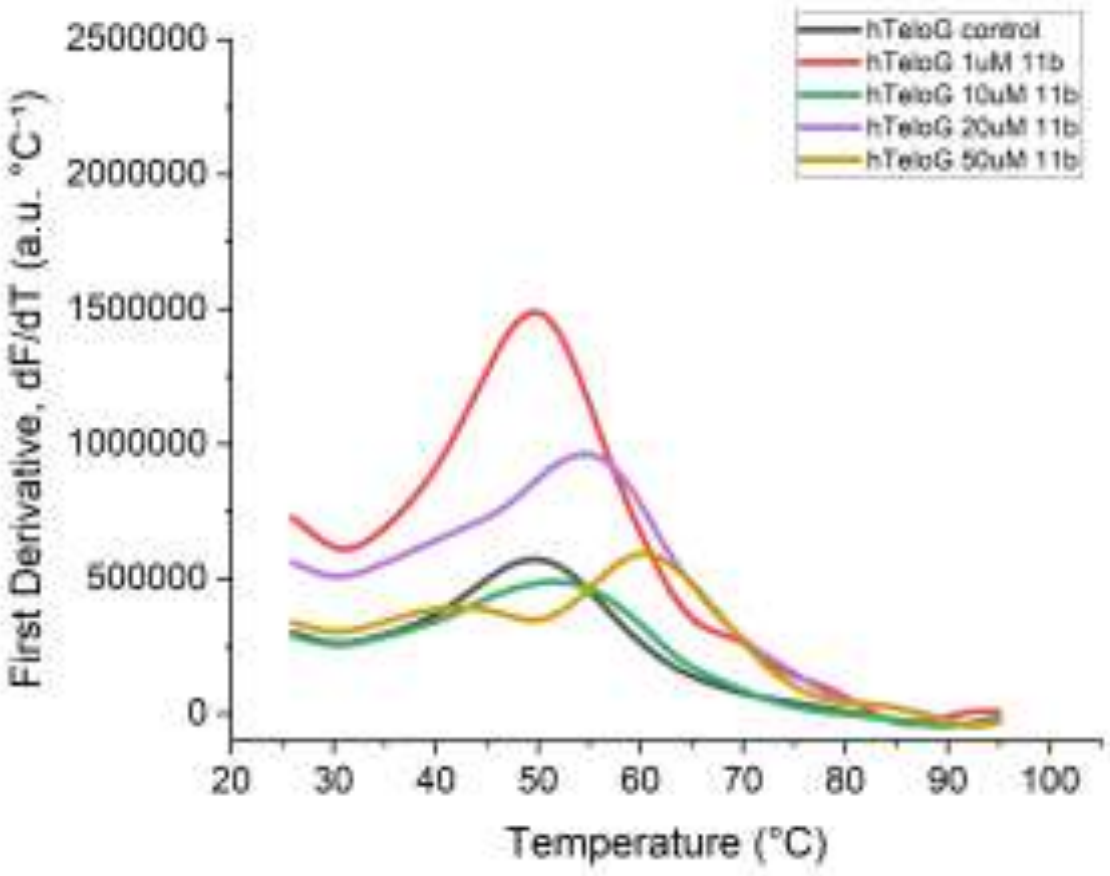
First Derivative, dF/dT (a.u. °C⁻¹)
Temperature (°C)
hTeloG control
hTeloG 1uM 11b
hTeloG 10uM 11b
hTeloG 20uM 11b
hTeloG 50uM 11b

E)

Table S3. FRET melting temperatures of hTeloG and dsDNA in the presence of increasing concentrations of compound 12a.

Experiment	DNA (0.2 µM)	Transition (°C)	Control	Compound 12a concentration (µM)				
				**1**	**5**	**10**	**20**	**50**
1	dsDNA	T_m	50	51	51	52	54	66
	hTeloG	T_m1	50	N/A	N/A	44	45	45
		T_m2		N/A	44	80	84	90
2	dsDNA	T_m	51	51	52	52	55	67
	hTeloG	T_m1	51	36	35	38	43	53
		T_m2		N/A	69	81	84	90

Figure S3. Representative FRET melting analysis of dsDNA and hTeloG in the presence of increasing concentrations of compound 12a. (A) Fluorescence melting curves of dsDNA. (B) Corresponding first-derivative curves of dsDNA. (C) Fluorescence melting curves of hTeloG. (D) Corresponding first-derivative curves of hTeloG shown over the full y-axis range. (E) Enlarged view of the hTeloG first-derivative curves using a reduced y-axis scale to visualise the lower-intensity transitions. Compound concentrations were 0, 1, 5, 10, 20, and 50 µM. Representative data from one of two independent experiments are shown.

A)

B)

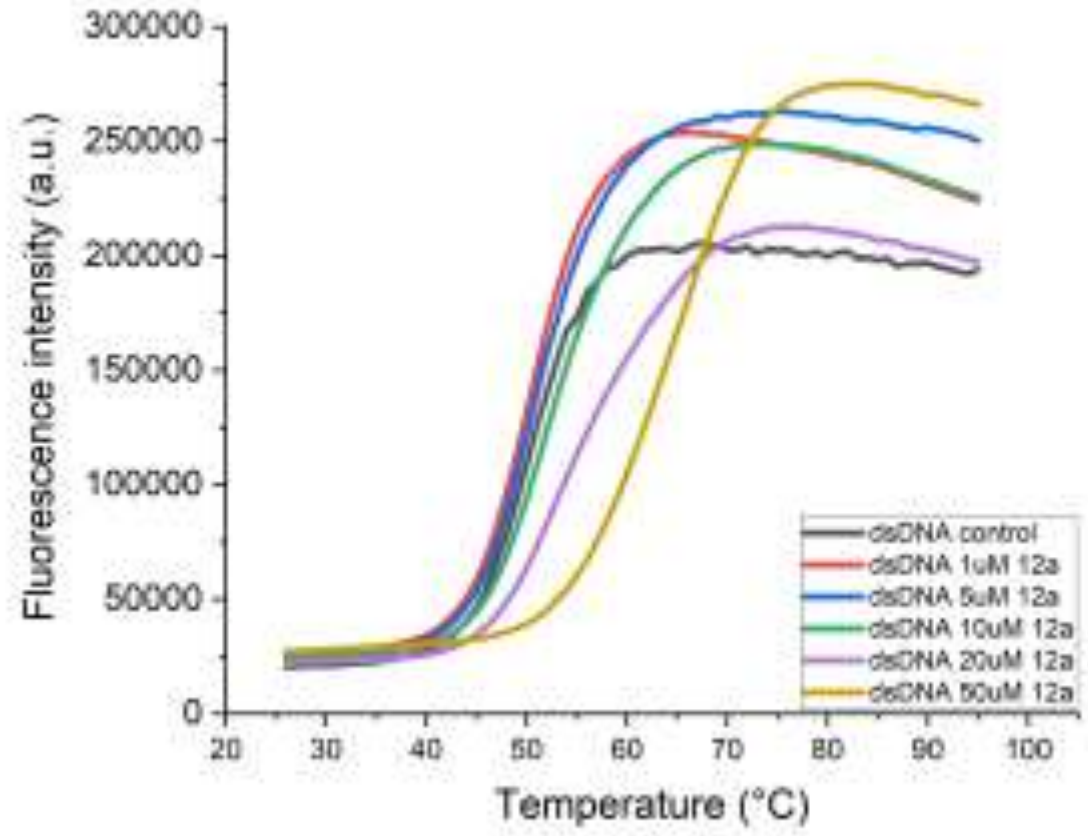
Fluorescence intensity (a.u.)
Temperature (°C)
dsDNA control
dsDNA 1uM 12a
dsDNA 5uM 12a
dsDNA 10uM 12a
dsDNA 20uM 12a
dsDNA 50uM 12a

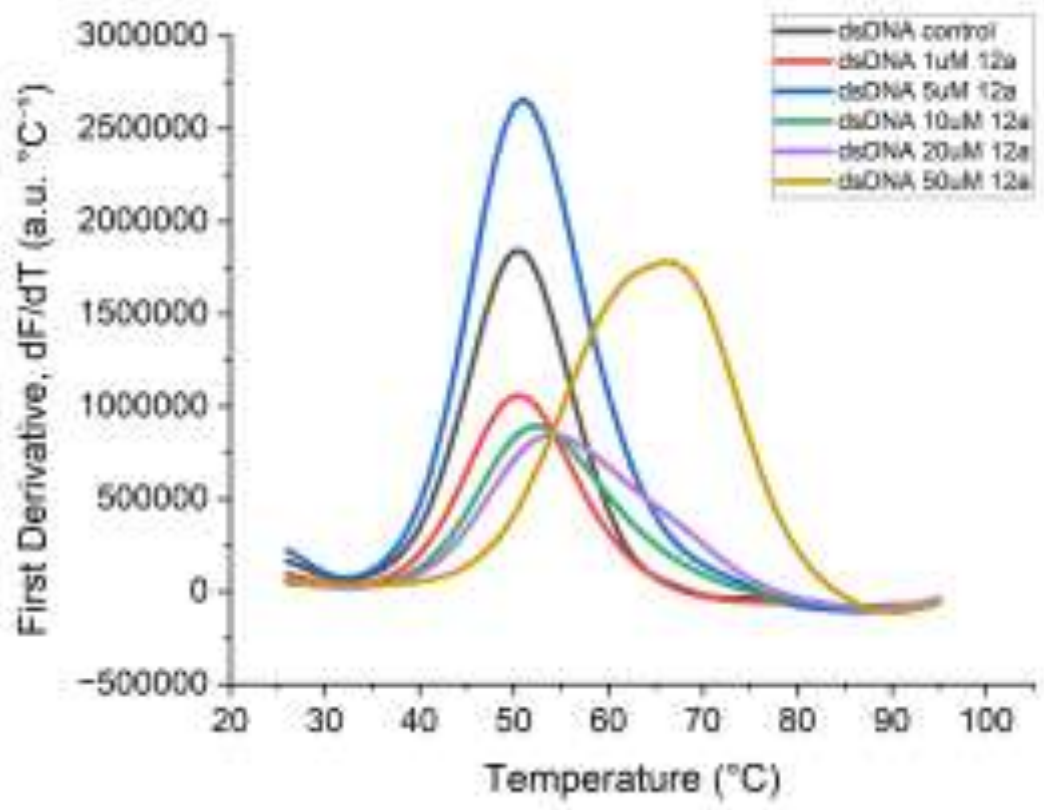
First Derivative, dF/dT (a.u. °C⁻¹)
Temperature (°C)
dsDNA control
dsDNA 1uM 12a
dsDNA 5uM 12a
dsDNA 10uM 12a
dsDNA 20uM 12a
dsDNA 50uM 12a

C)

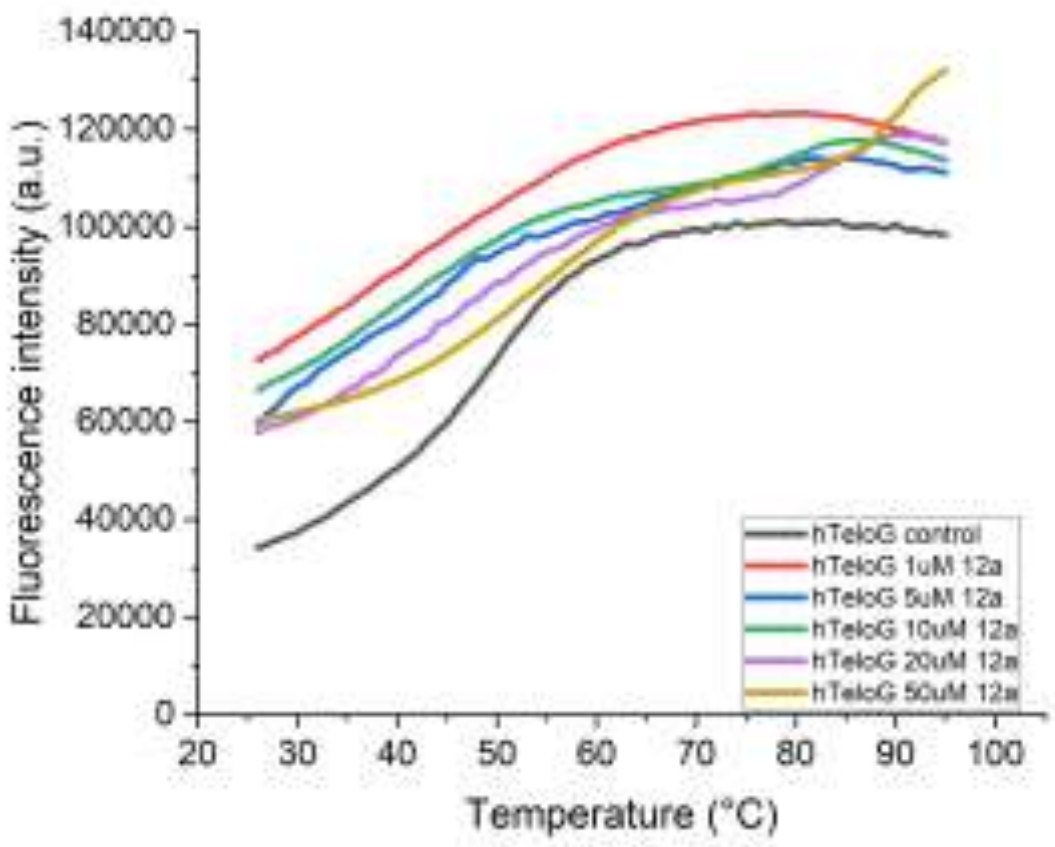
Fluorescence intensity (a.u.)
Temperature (°C)
hTeloG control
hTeloG 1uM 12a
hTeloG 5uM 12a
hTeloG 10uM 12a
hTeloG 20uM 12a
hTeloG 50uM 12a

D)

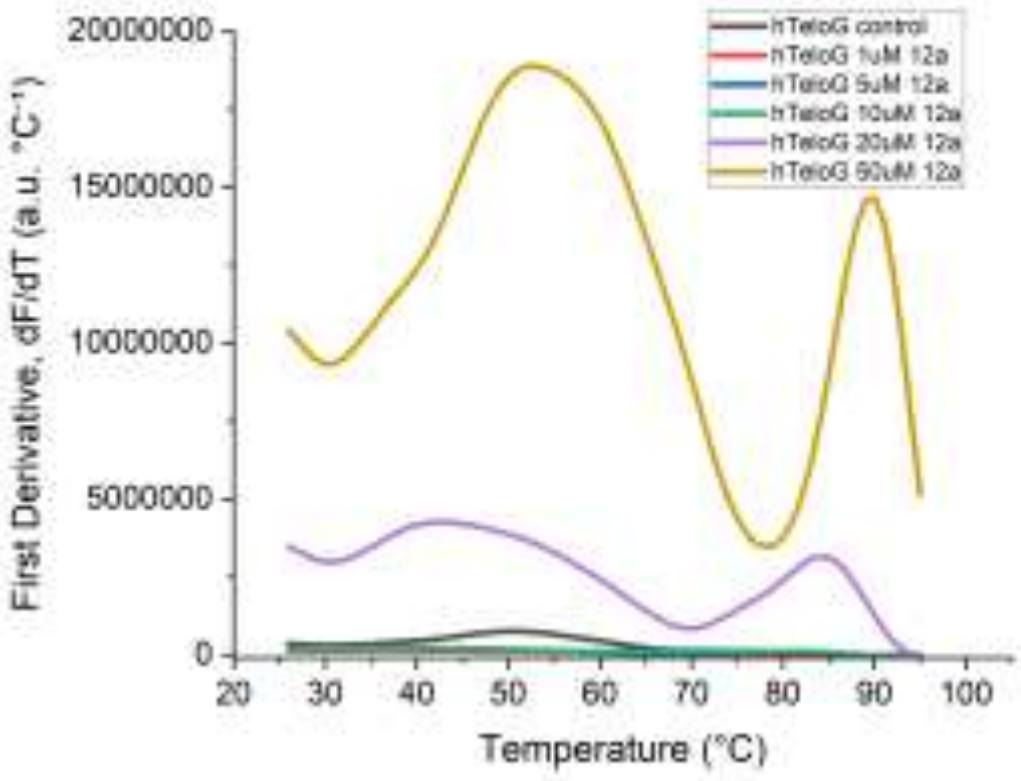
First Derivative, dF/dT (a.u. °C⁻¹)
Temperature (°C)
hTeloG control
hTeloG 1uM 12a
hTeloG 5uM 12a
hTeloG 10uM 12a
hTeloG 20uM 12a
hTeloG 50uM 12a

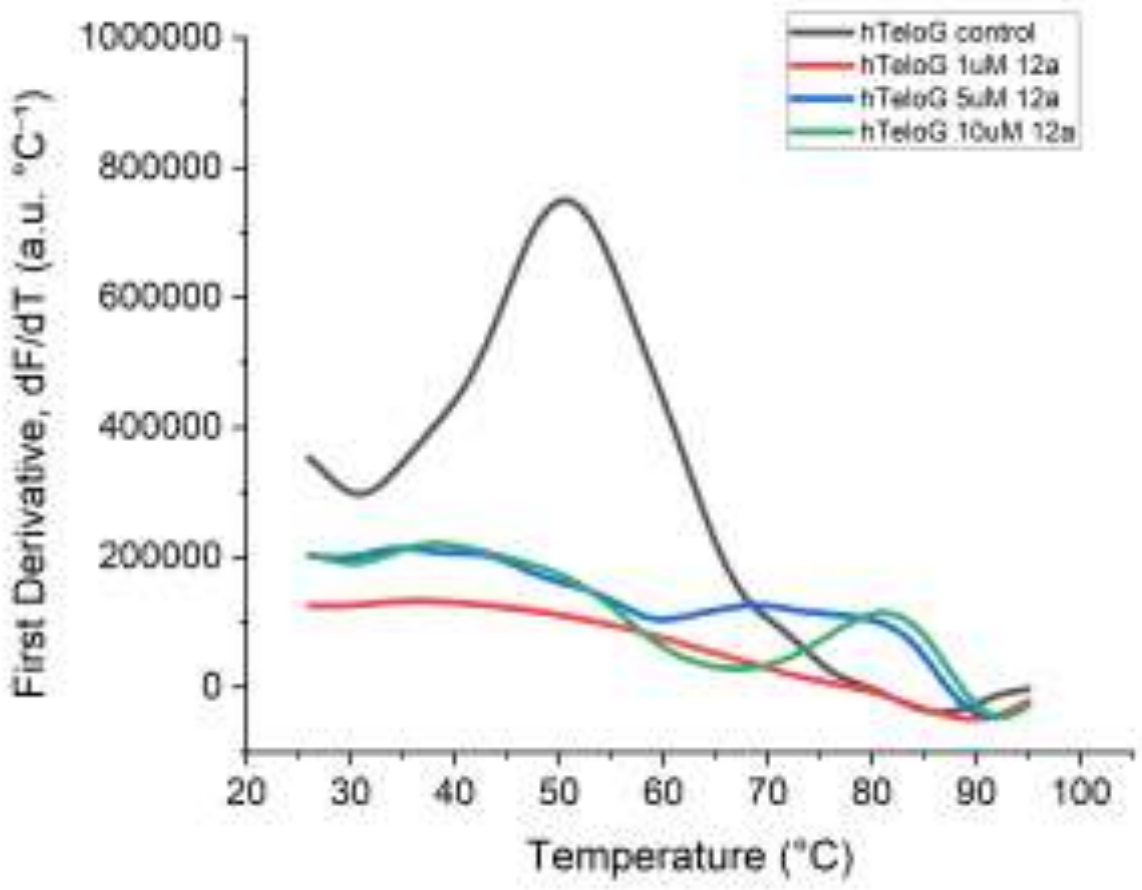

hTeloG control
hTeloG 1uM 12a
hTeloG 5uM 12a
hTeloG 10uM 12a
First Derivative, dF/dT (a.u. °C⁻¹)
Temperature (°C)

E)

Table S4. FRET melting temperatures of hTeloG and dsDNA in the presence of increasing concentrations of compound 12b.

Experiment	DNA (0.2 µM)	Transition (°C)	Control	Compound 12b concentration (µM)				
				1	5	10	20	50
1	dsDNA	T_m	51	51	53	53	56	65
	hTeloG	T_m1		N/A	N/A	44	45	45
		T_m2	49	52	59	70	74	83
2	dsDNA	T_m	50	51	52	53	54	63
	hTeloG	T_m1	49			45	46	49
		T_m2		48	50	62	71	84

Figure S4. Representative FRET melting analysis of dsDNA and hTeloG in the presence of increasing concentrations of compound 12b. (A) Fluorescence melting curves of dsDNA. (B) Corresponding first-derivative curves of dsDNA. (C) Fluorescence melting curves of hTeloG. (D) Corresponding first-derivative curves of hTeloG shown over the full y-axis range. (E) Enlarged view of the hTeloG first-derivative curves using a reduced y-axis scale to visualise the lower-intensity transitions. Compound concentrations were 0, 1, 5, 10, 20, and 50 µM. Representative data from one of two independent experiments are shown.

A)

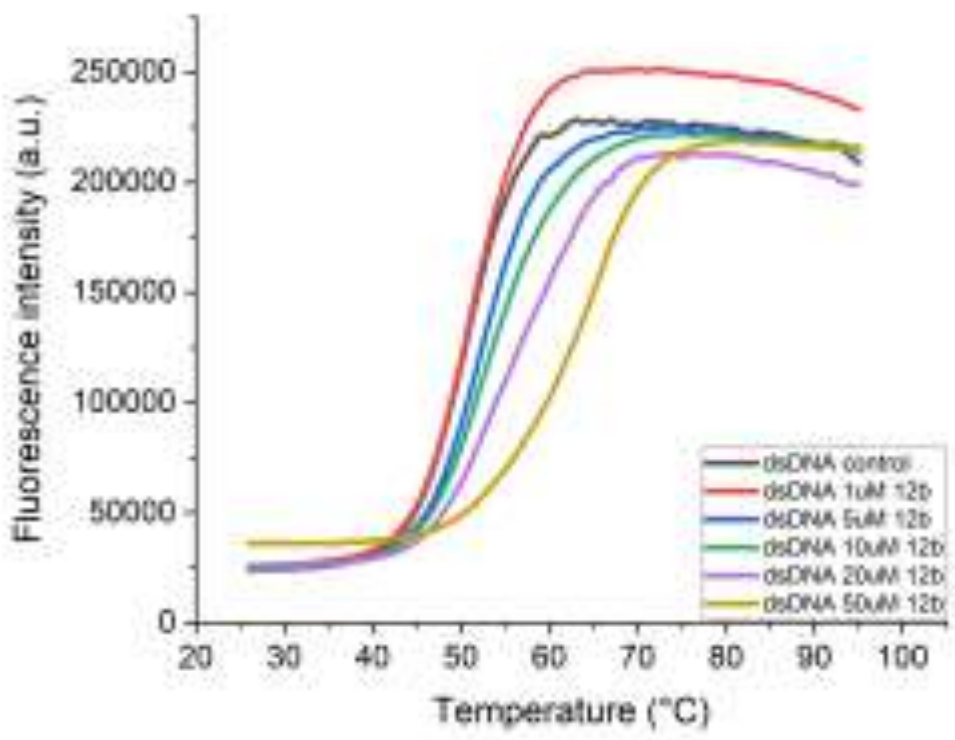

B)

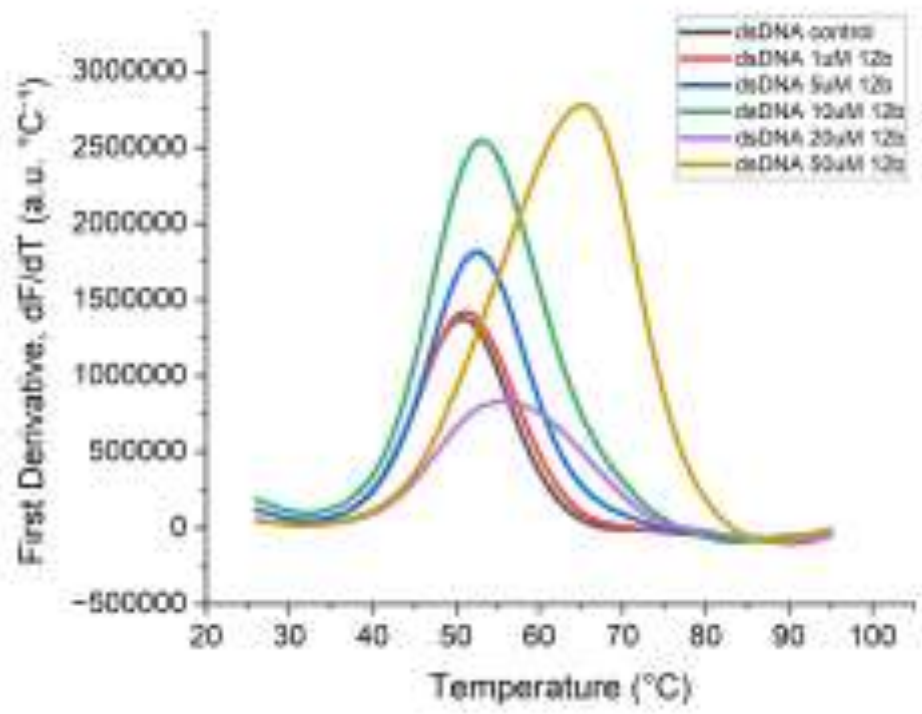

C)

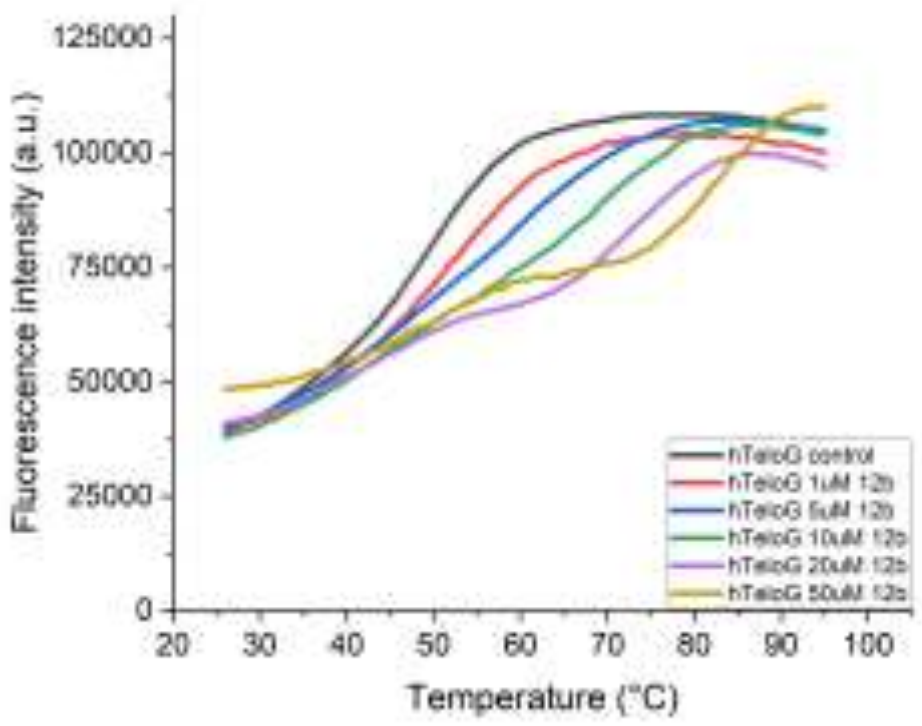

125000
100000
75000
50000
25000
0
Fluorescence intensity (a.u.)
20 30 40 50 60 70 80 90 100
Temperature (°C)
hTeloG control
hTeloG 1uM 12b
hTeloG 5uM 12b
hTeloG 10uM 12b
hTeloG 20uM 12b
hTeloG 50uM 12b

D)

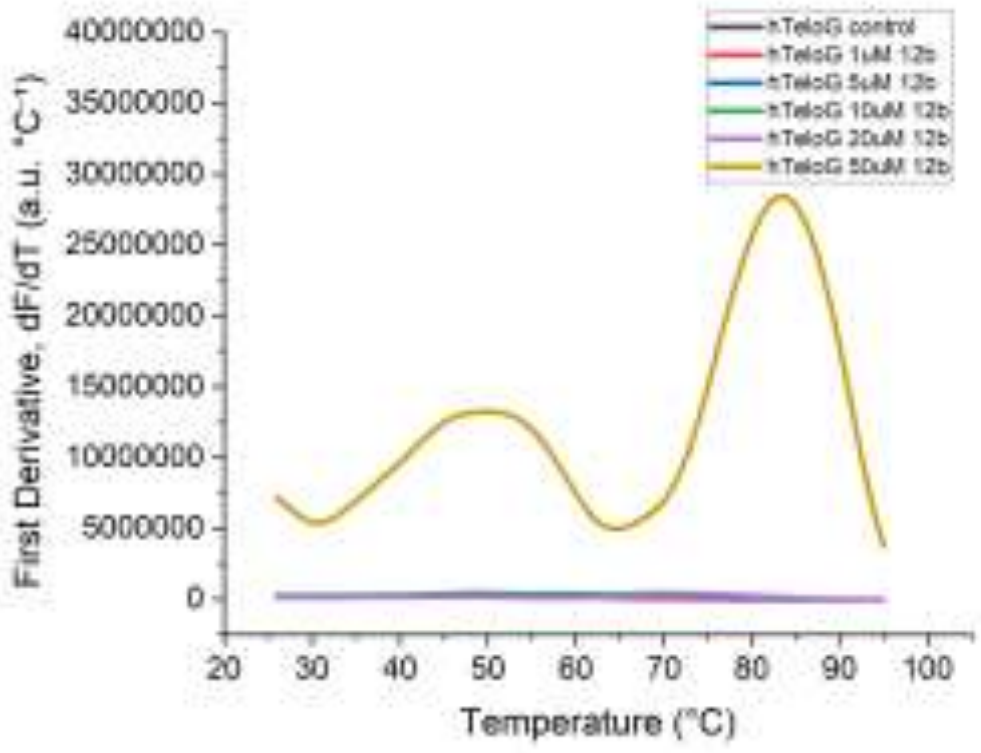

40000000
35000000
30000000
25000000
20000000
15000000
10000000
5000000
0
First Derivative, dF/dT (a.u. °C⁻¹)
20 30 40 50 60 70 80 90 100
Temperature (°C)
hTeloG control
hTeloG 1uM 12b
hTeloG 5uM 12b
hTeloG 10uM 12b
hTeloG 20uM 12b
hTeloG 50uM 12b

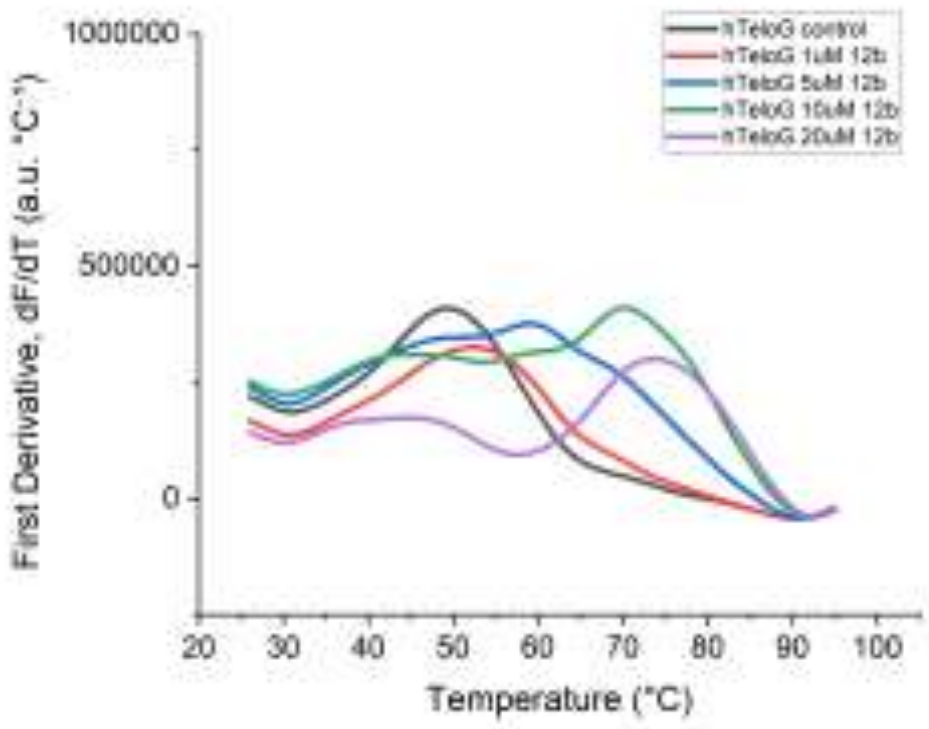

1000000
500000
0
First Derivative, dF/dT (a.u. °C⁻¹)
20 30 40 50 60 70 80 90 100
Temperature (°C)
hTeloG control
hTeloG 1uM 12b
hTeloG 5uM 12b
hTeloG 10uM 12b
hTeloG 20uM 12b

E)

Table S5. FRET melting temperatures of hTeloG and dsDNA in the presence of increasing concentrations of compound 12c.

Experiment	DNA (0.2 µM)	Transition (°C)	Control	Compound 12c concentration (µM)				
				1	5	10	20	50
1	dsDNA	T_m	51	51	52	53	57	66
	hTeloG	T_m1	49	N/A	N/A	45	42	48
		T_m2		50	60	68	74	83
2	dsDNA	T_m	50	51	52	52	56	67
	hTeloG	T_m1	49	N/A	N/A	43	46	51
		T_m2		49	52	65	73	86

Figure S5. Representative FRET melting analysis of dsDNA and hTeloG in the presence of increasing concentrations of compound 12c. (A) Fluorescence melting curves of dsDNA. (B) Corresponding first-derivative curves of dsDNA. (C) Fluorescence melting curves of hTeloG. (D) Corresponding first-derivative curves of hTeloG shown over the full y-axis range. (E) Enlarged view of the hTeloG first-derivative curves using a reduced y-axis scale to visualise the lower-intensity transitions. Compound concentrations were 0, 1, 5, 10, 20, and 50 µM. Representative data from one of two independent experiments are shown.

A)

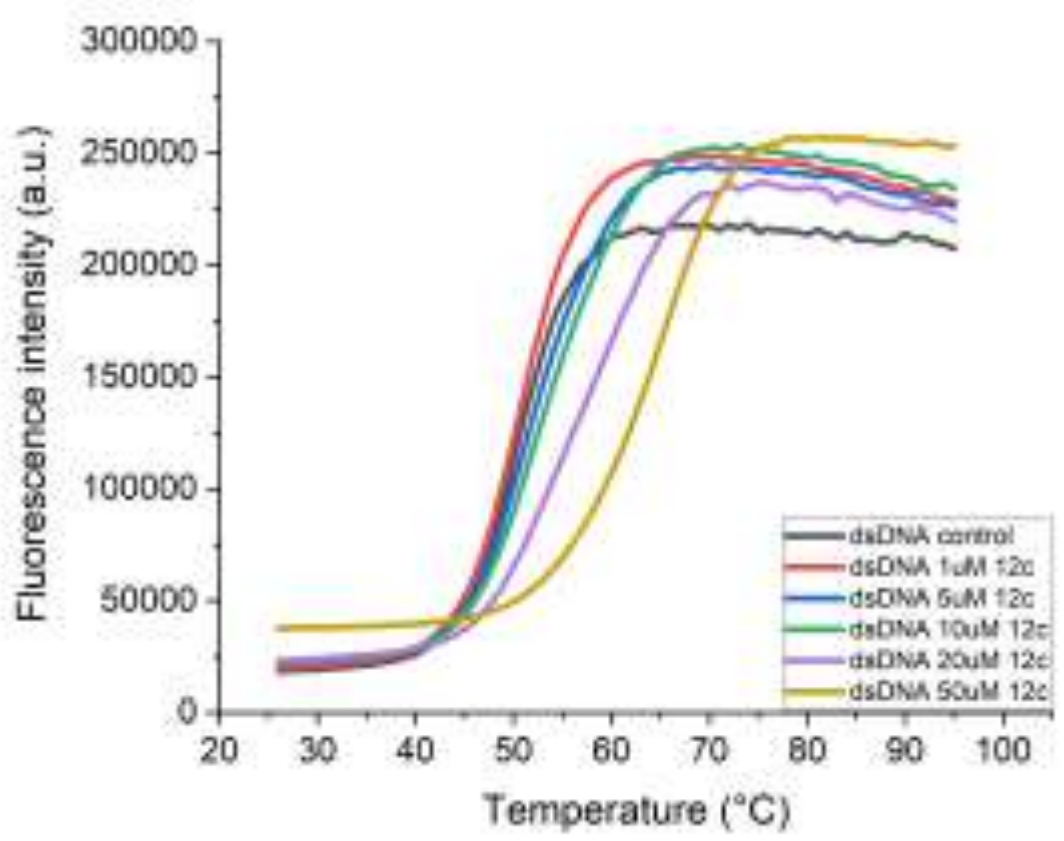

B)

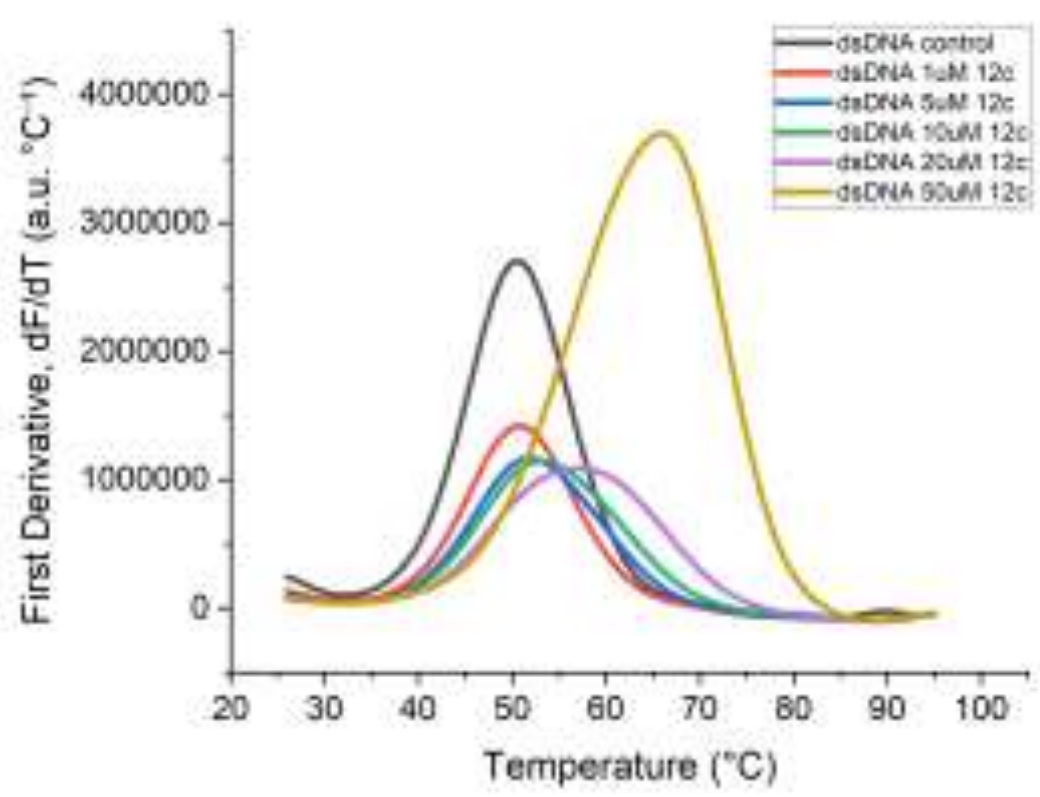

C)

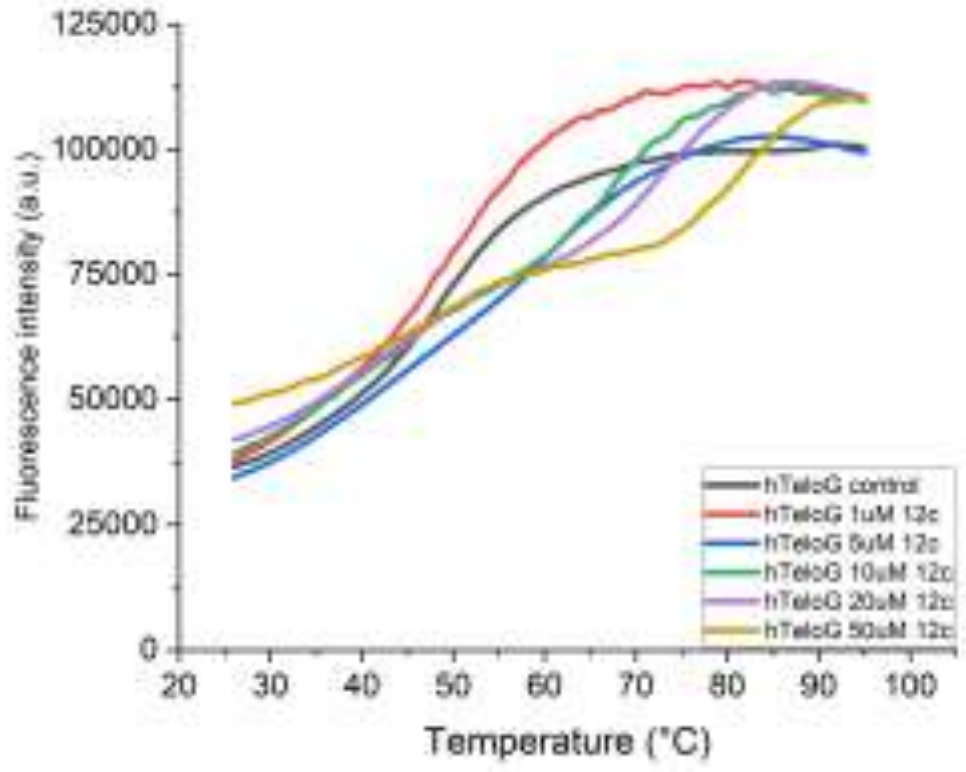

Fluorescence intensity (a.u.)
Temperature (°C)
hTeloG control
hTeloG 1uM 12c
hTeloG 5uM 12c
hTeloG 10uM 12c
hTeloG 20uM 12c
hTeloG 50uM 12c

D)

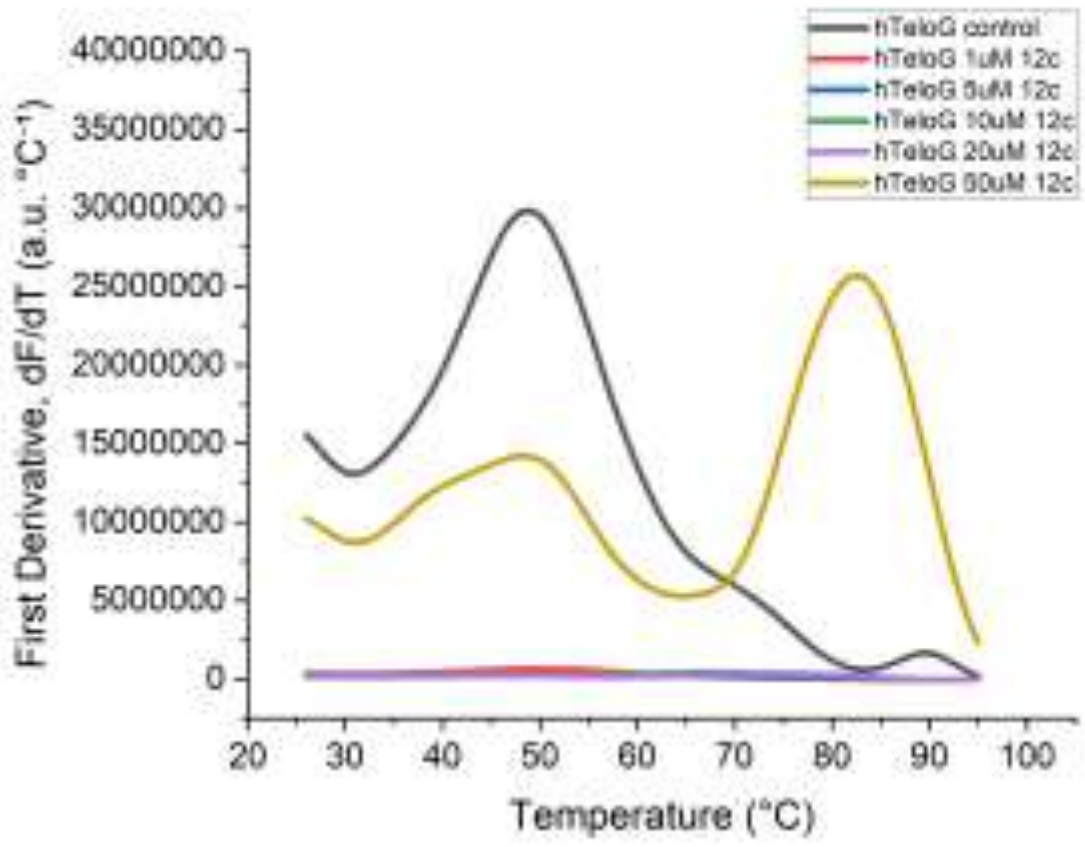

First Derivative, dF/dT (a.u. °C⁻¹)
Temperature (°C)
hTeloG control
hTeloG 1uM 12c
hTeloG 5uM 12c
hTeloG 10uM 12c
hTeloG 20uM 12c
hTeloG 50uM 12c

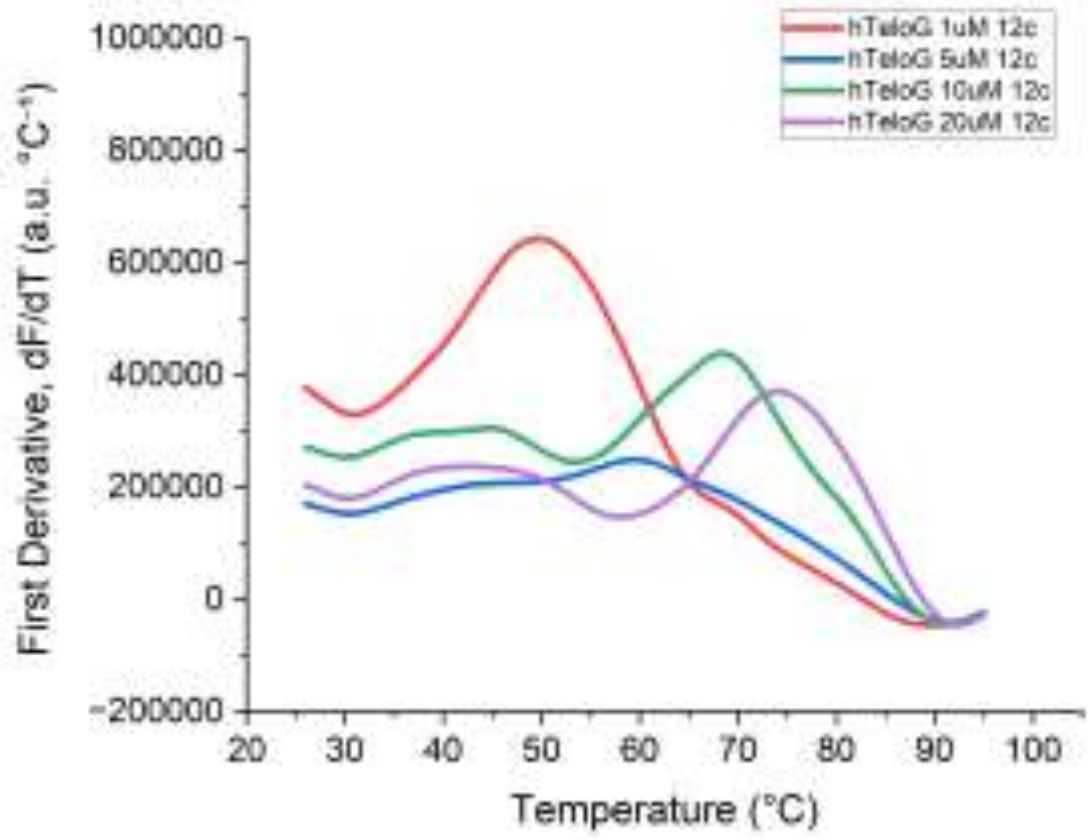

First Derivative, dF/dT (a.u. °C⁻¹)
Temperature (°C)
hTeloG 1uM 12c
hTeloG 5uM 12c
hTeloG 10uM 12c
hTeloG 20uM 12c

E)

Anti-parasite assays (*T. brucei*)

T. brucei parasites were grown in HMI-11 medium with 10% heat-inactivated foetal bovine serum with compounds **11a**, **11b** and **12a-c** added[13] Parasites were counted using CASY TT Cell Counter. Fluorescence was recorded using a FLx 800 plate reader (BioTek) with an excitation wavelength 530–535 nm and emission wavelength of 590–610 nm using Gen5 Reader Control 2.0 Software (BioTek). EC_{50} values were determined using a 4-parameter (4 replicates) non-linear logistic regression equation using GraFit 5.0 (Erithacus Software). SD values were calculated based on curve fitting to n biological replicates performed in parallel.

References for Supplementary Information

1. J. L. Clark, R. M. Neyyappadath, C. Yu, A. M. Z. Slawin, D. B. Cordes, D. O'Hagan, *Chem. Eur. J.*, **2021**, *27*, 16000 - 16005.
2. Y. V. Suseela, M. Sasikumar, T. Govindaraju, *Tetrahedron Lett.*, **2013**, *54*, 6314 - 6318.
3. F. Doria, I. Manet, V. Grande, S. Monti, M. Freccero, *J. Org. Chem.*, **2013**, *78*, 8065 - 8073.
4. M. Arévalo-Ruiz, F. Doria, E. Belmonte-Reche, A. De Rache, J. Campos-Salinas, R. Lucas, E. Falomir, M. Carda, J. M. Pérez-Victoria, J. Mergny, M. Freccero, J. C. Morales, *Chem. Eur. J.*, **2017**, *23*, 2157 - 2164.
5. C. Marchetti, K. G. Zyner, S. A. Ohnmacht, M. Robson, S. M. Haider, J. P. Morton, G. Marsico, T. Vo, S. Laughlin-Toth, A. A. Ahmed, G. Di Vita, I. Pazitna, M. Gunaratnam, R. J. Besser, A. C. G. Andrade, S. Diocou, J. A. Pike, D. Tannahill, R. B. Pedley, T. R. J. Evans, W. D. Wilson, S. Balasubramanian, S. Neidle, *J. Med. Chem.*, **2018**, *61*, 2500 - 2517.
6. L. S. Vogl, P. Mayer, R. Robiette and M. Waser, *Angew. Chem. Int. Ed.*, **2023**, *63*, e202315345.
7. A. Monopoli, M. Casiello, P. Cotugno, A. Milella, F. Palumbo, F. Fracassi, A. Nacci, *Molecules*, **2021**, *26*, 5592.
8. J. Yang, J. Liu, Y. Ge, W. Huang, C. Schneider, R. Dühren, R. Franke, H. Neumann, R. Jackstell, M. Beller, *Chemical Commun.*, **2020**, *56*, 5235 - 5238.
9. A. S. Cantrell, P. Engelhardt, M. Högberg, S. R. Jaskunas, N. G. Johansson, C. L. Jordan, J. Kangasmetsä, M. D. Kinnick, P. Lind, J. M. Morin, M. A. Muesing, R. Noreén, B. Öberg, P. Pranc, C. Sahlberg, R. J. Ternansky, R. T. Vasileff, L. Vrang, S. J. West, H. Zhang, *J. Med. Chem.*, **1996**, *39*, 4261 - 4274.
10. Y.-J. Chen, P. Xie, L. Zhang, A. Shi, Y. Qiu, Q.-L. Zhou, *J. Am. Chem. Soc.*, **2025**, *147*, 43737 - 43744.
11. Z. Fu, S. Huang, W. Su, M. Hong, *Org. Lett.*, **2010**, *12*, 4992 - 4995.
12. E. P. Wright, H. A. Day, A. M. Ibrahim, J. Kumar, L. J. E. Boswell, C. Huguin, C. E. M. Stevenson, K. Pors, Z. A. E. Waller, *Sci. Rep.*, **2016**, *6*, 1-7. doi:10.1038/srep39456.
13. P. E. Cockram, E. A. Dickie, M. P. Barrett, T. K. Smith, *PLOS NTDs*, **2020**, *14*, e0008928.

[END]
